# Supplementary material for: MinD-RNase E interplay controls localization of polar mRNAs in E. coli
Source: EMBO J. 2024 Jan 19;43(4):8. doi: 10.1038/s44318-023-00026-9 (PMC10897333; doi:10.1038/s44318-023-00026-9)
Supplement: Supplementary file 1 — Table EV1 [file 44318_2023_26_MOESM1_ESM.pdf]

Table EV1. Strains used in this study

Strain	Parental strain	Genotype	Source
MG1655	-	*Escherichia coli* K-12 MG1655 (F-, λ-, *rph-1*)	Lab collection
MG1655*Δhfq*	MG1655	*Δhfq::Kan*	(Kannaiah, Livny et al. 2019)
BL21(DE3)	-	*Escherichia coli* BL21(DE3)	(Studier; Moffatt, 1986)
BTH101	-	*F', cya-99*, *araD139*, *galE15*, *galK16*, *rpsL1 (Str^R)*, *hsdR2*, *mcrA1*, *mcrB1*, *relA1*	(Karimova; Gauliard; Davi; Ouellette *et al.*, 2017)
FB76	-	mreB-RFP^{sw} *yhd<>cat*	(Bendezú; Hale; Bernhardt; De Boer, 2009)
W3899	-	F$^-$, *glnX44*(AS), *nadB7*	(Romantsov; Helbig; Culham; Gill *Et Al.*, 2007)
WC3899	W3899	*cls::Tn10*	(Romantsov; Helbig; Culham; Gill *Et Al.*, 2007)
PB103		*dadR trpE trpA tna*	(Raskin; De Boer, 1999b)
PB114	PB103	*ΔminCDE::kan*	(Raskin; De Boer, 1999b)
MG1655*ΔminCDE*	MG1655	*ΔminCDE::kan*	This work
MG1655*ΔminC*	MG1655	*ΔminC*	This work
Kti162	-	*rne-mCherry*	(Strahl; Turlan; Khalid; Bond *Et Al.*, 2015)
N3433	-	*Hfr(PO1)*, *lacZ43(Fs)*, *λ$^-$*,*relA1*,*spoT1*, *thiE1*	(Goldblum; Apririon, 1981)
N3431	N3433	*rne3071(ts)*	(Goldblum; Apririon, 1981)
MG1655*Δpnp*	MG1655	*Δpnp::kan*	This work
MG1655*ΔrhlB*	MG1655	*ΔrhlB::kan*	This work

K10	-	Hfr(PO2A), *garB10*, *fhuA22*, *ompF627*(T2R), *fadL701*(T2R), *rel1*, *pitA10*, *spoT1*, *rrnB-2*, *mcrB1*, *rob-1*, *creC510*	(Hillman; Fraenkel, 1975)
DF261	K10	*eno-2*	(Hillman; Fraenkel, 1975)
χ1488	-	F-, *purE41, glnV42(AS), λ-, serC53, minB-2, his-53, xyl-14, metB65, cycA1, hsdR2, tte-1, cycB2, ilv-277*	(Lai; Nair; Phadke; Maddock, 2004)
Kti162 *ΔminD*	Kti162	*rne-mCherry, ΔminD*	This work
MG1655 CheA-YFP	MG1655	cheA(1-146)-myfp-cheA(147-end)	(Koler; Peretz; Aditya; Shimizu *et al.*, 2018)

References

1. Bendezú FO, Hale CA, Bernhardt TG, de Boer PA. 2009. Rodz (yfga) is required for proper assembly of the mreb actin cytoskeleton and cell shape in e. Coli. Embo j. 28(3):193-204.
2. Goldblum K, Apririon D. 1981. Inactivation of the ribonucleic acid-processing enzyme ribonuclease e blocks cell division. J Bacteriol. 146(1):128-132.
3. Hillman JD, Fraenkel DG. 1975. Glyceraldehyde 3-phosphate dehydrogenase mutants of escherichia coli. J Bacteriol. 122(3):1175-1179.
4. Kannaiah, S., Livny, J., and Amster-Choder, O. (2019). Spatiotemporal Organization of the E. coli Transcriptome: Translation Independence and Engagement in Regulation. Molecular Cell 76, 574-589.e7. 10.1016/j.molcel.2019.08.013.
5. Karimova G, Gauliard E, Davi M, Ouellette SP, Ladant D. 2017. Protein-protein interaction: Bacterial two-hybrid. Methods in molecular biology. 1615:159-176.
6. Koler M, Peretz E, Aditya C, Shimizu TS, Vaknin A. 2018. Long-term positioning and polar preference of chemoreceptor clusters in e. Coli. Nat Commun. 9(1):4444.
7. Lai EM, Nair U, Phadke ND, Maddock JR. 2004. Proteomic screening and identification of differentially distributed membrane proteins in escherichia coli. Mol Microbiol. 52(4):1029-1044.
8. Raskin DM, de Boer PA. 1999. Rapid pole-to-pole oscillation of a protein required for directing division to the middle of escherichia coli. Proc Natl Acad Sci U S A. 96(9):4971-4976.
9. Romantsov T, Helbig S, Culham DE, Gill C, Stalker L, Wood JM. 2007. Cardiolipin promotes polar localization of osmosensory transporter prop in escherichia coli. Mol Microbiol. 64(6):1455-1465.

10. Strahl H, Turlan C, Khalid S, Bond PJ, Kebalo JM, Peyron P, Poljak L, Bouvier M, Hamoen L, Luisi BF et al. 2015. Membrane recognition and dynamics of the rna degradosome. PLoS Genet. 11(2):e1004961.
11. Studier FW, Moffatt BA. 1986. Use of bacteriophage t7 rna polymerase to direct selective high-level expression of cloned genes. J Mol Biol. 189(1):113-130.
