# Supplementary material for: MinD-RNase E interplay controls localization of polar mRNAs in E. coli
Source: EMBO J. 2024 Jan 19;43(4):8. doi: 10.1038/s44318-023-00026-9 (PMC10897333; doi:10.1038/s44318-023-00026-9)
Supplement: Supplementary file 2 — Table EV2 [file 44318_2023_26_MOESM2_ESM.pdf]

Table EV2. Plasmids used in this study

Resource	Parental plasmid	Ori	Resistance	Overexpression	Source	Identifier
pCP20		pSC101	Amp, Cm		(Cherepanov; Wackernagel, 1995)	λpR–FLP λcI857^{+} RepA101(*ts*) *bla cat*
pKD46		R101	Amp		(Datsenko; Wanner, 2000)	
pKD4		R6K γ	Amp, Kan		(Datsenko; Wanner, 2000)	
pZA25-GFP		p15A	Kan	MS2-GFP	(Nevo-Dinur; Nussbaum-Shochat; Ben-Yehuda; Amster-Choder, 2011)	MS2-GFP
pEL8	pCP20	pSC101	Amp		(Libby; Ekici; Goulian, 2010)	λpR–FLP λcI857^{+} Rep^{ts} *bla*
pGSmGFP-MinD		ColE1	Amp	mGFP-MinD	Lab collection	mGFP-MinD
pVK207		pSC101	Spec	*rne-yfp*	(Khemici; Poljak; Luisi; Carpousis, 2008)	*rne-yfp*
pVK221		pSC101	Spec	*rneΔmts-yfp*	(Khemici; Poljak; Luisi; Carpousis, 2008)	*rneΔmts-yfp*
pZE12-*bglG*$_{6xbs}$	pZE12-*6xbs*	ColE1	Amp	*bglG*$_{6xbs}$	(Kannaiah, Livny et al. 2019)	*bglG*$_{6xbs}$
pZE12-*cheA*$_{6xbs}$	pZE12-*6xbs*	ColE1	Amp	*bglG*$_{6xbs}$	(Kannaiah, Livny et al. 2019)	*cheA*$_{6xbs}$
pZE12-*motA*$_{6xbs}$	pZE12-*6xbs*	ColE1	Amp	*cheA*$_{6xbs}$	(Kannaiah, Livny et al. 2019)	*motA*$_{6xbs}$
pZE12-*motB*$_{6xbs}$	pZE12-*6xbs*	ColE1	Amp	*motA*$_{6xbs}$	(Kannaiah, Livny et al. 2019)	*motB*$_{6xbs}$

pZE12-*oppC*$_{6xbs}$	pZE12-*6xbs*	ColE1	Amp	*motB*$_{6xbs}$	(Kannaiah, Livny et al. 2019)	*oppC*$_{6xbs}$
pZE12-*oppD*$_{6xbs}$	pZE12-*6xbs*	ColE1	Amp	*oppC*$_{6xbs}$	(Kannaiah, Livny et al. 2019)	*oppD*$_{6xbs}$
pZE12-*oppF*$_{6xbs}$	pZE12-*6xbs*	ColE1	Amp	*oppD*$_{6xbs}$	(Kannaiah, Livny et al. 2019)	*oppF*$_{6xbs}$
pZE12-*cspG*$_{6xbs}$	pZE12-*6xbs*	ColE1	Amp	*oppF*$_{6xbs}$	(Kannaiah, Livny et al. 2019)	*cspG*$_{6xb}$
pZE12-*proP*$_{6xbs}$	pZE12-*6xbs*	ColE1	Amp	*cspG*$_{6xb}$	(Kannaiah, Livny et al. 2019)	*proP*$_{6xbs}$
pZE12-*6xbs*	pZE12-luc	ColE1	Amp	*proP*$_{6xbs}$	(Kannaiah, Livny et al. 2019)	*6xbs*
pET15b	-	pMB1	Amp	*6xbs*	Novagen	Derivative of pET11a with N-terminal his-tag
pET15b-FLAGminD	pET15b	pMB1	Amp	FLAG-MinD	This work	
pET15b-rne$^{378-659}$	pET15b	pMB1	Amp	RNase E$^{378-659}$	This work	RNE’
pET15b-rne$^{378-659}$ΔA	pET15b	pMB1	Amp	RNase E$^{378-659}$ΔA	This work	RNE’ΔA
pKT25	-	p15A	Kan	-	(Karimova; Gauliard; Davi; Ouellette *et al.*, 2017)	T25
pUT18C	-	ColE1	Amp	-	(Karimova; Gauliard; Davi; Ouellette *et al.*, 2017)	T18
pKT25-zip	pKT25	p15A	Kan	Zip	(Karimova; Gauliard; Davi; Ouellette *et al.*, 2017)	T25-Zip

pUT18C-zip	pUT18C	ColE1	Amp	Zip	(Karimova; Gauliard; Davi; Ouellette *et al.*, 2017)	T18-Zip
pKT25-minD	pKT25	p15A	Kan	MinD	This work	25-MinD
pUT18C-rne$^{378\text{-}659}$	pUT18C	ColE1	Amp	RNase E$^{378\text{-}659}$	This work	T18-RNE’
pUT18C-rne$^{378\text{-}659}\Delta$A	pUT18C	ColE1	Amp	RNase E$^{378\text{-}659}\Delta$A	This work	T18-RNE’ΔA
pKT25-minD^{L48R}	pKT25	p15A	Kan	MinD^{L48R}	This work	T25-MinD^{L48R}
pKT25-minD^{V147R}	pKT25	p15A	Kan	MinD^{V147R}	This work	T25-MinD^{V147R}
pKT25-minD^{L194R}	pKT25	p15A	Kan	MinD^{L194R}	This work	T25-MinD^{L194R}
pDR150	-	ColE1	Amp	MinD^{WT}	(Raskin; De Boer, 1999a)	pMinD^{WT}
pDR186	-	ColE1	Amp	MinD^{K16Q}	(Raskin; De Boer, 1999a)	pMinD^{K16Q}
pGP12-Tar-mYFP	pKG116		Amp	Tar-mYFP	(Piñas; Desantis; Parkinson, 2018)	Tar-mYFP

References

1. Cherepanov PP, Wackernagel W. 1995. Gene disruption in escherichia coli: Tcr and kmr cassettes with the option of flp-catalyzed excision of the antibiotic-resistance determinant. Gene. 158(1):9-14.
2. Datsenko KA, Wanner BL. 2000. One-step inactivation of chromosomal genes in escherichia coli k-12 using pcr products. Proc Natl Acad Sci U S A. 97(12):6640-6645.
3. Kannaiah, S., Livny, J., and Amster-Choder, O. (2019). Spatiotemporal Organization of the E. coli Transcriptome: Translation Independence and Engagement in Regulation. Molecular Cell 76, 574-589.e7. 10.1016/j.molcel.2019.08.013.
4. Karimova G, Gauliard E, Davi M, Ouellette SP, Ladant D. 2017. Protein-protein interaction: Bacterial two-hybrid. Methods in molecular biology. 1615:159-176.
5. Khemici V, Poljak L, Luisi BF, Carpousis AJ. 2008. The rnase e of escherichia coli is a membrane-binding protein. Mol Microbiol. 70(4):799-813.

6. Libby EA, Ekici S, Goulian M. 2010. Imaging ompr binding to native chromosomal loci in escherichia coli. J Bacteriol. 192(15):4045-4053.
7. Nevo-Dinur K, Nussbaum-Shochat A, Ben-Yehuda S, Amster-Choder O. 2011. Translation-independent localization of mrna in e. Coli. Science. 331(6020):1081-1084.
8. Piñas GE, DeSantis MD, Parkinson JS. 2018. Noncritical signaling role of a kinase-receptor interaction surface in the escherichia coli chemosensory core complex. J Mol Biol. 430(7):1051-1064.
9. Raskin DM, de Boer PA. 1999. Minde-dependent pole-to-pole oscillation of division inhibitor minc in escherichia coli. J Bacteriol. 181(20):6419-6424.
