# Supplementary material for: MinD-RNase E interplay controls localization of polar mRNAs in E. coli
Source: EMBO J. 2024 Jan 19;43(4):8. doi: 10.1038/s44318-023-00026-9 (PMC10897333; doi:10.1038/s44318-023-00026-9)
Supplement: Supplementary file 3 — Table EV3 [file 44318_2023_26_MOESM3_ESM.pdf]

Table EV3. Oligonucleotides used in this study

A. Oligonucleotides used for plasmid and strain construction

Resource	Sequence	Use
F-vector pET15b	CTCGAGGATCCGGCTGCTAAC	pET15b-FLAG-minD construction (vector)
R-vector pET15b	CCCCTGAAAGTAAAGATTCTCC	pET15b-FLAG-minD construction (vector)
F-insert	TATGGAGAATCTTTACTTTCAGGGGatgGCACGCATTATTGTTG	pET15b-FLAG-minD construction (insert)
R-insert	GGCTTTGTTAGCAGCCGGATCCTCGAGttaTCCTCCGAACAAGCGTTTG	pET15b-FLAG-minD construction (insert)
F-PstI-*minD* pKT25	CAACTGCAGccGCTGCACGCATTATTGTTGTTACTTCG	pKT25-minD construction
R-BamHI *minD*	GTGGGATCCttaTCCTCCGAACAAGCGTTTGAGG	pKT25-minD construction
F-PstI-*rne* pUT18C	CAACTGCAGcGCTGCTGCTCATATTTCTCGCTTTGGCC	pUT18C- rne$^{378\text{-}659}$ and pUT18C- rne$^{378\text{-}659}\Delta$A construction
R-BamHI *rne*	GTGGGATCCttaCTGCTGACGGCTCTCACG	pUT18C- rne$^{378\text{-}659}$ and pUT18C- rne$^{378\text{-}659}\Delta$A construction
F-pkT25(EcoRI)	ctaagaattcggccgtcg	pKT25-minD point mutations construction (vector)
R-pkT25(KpnI)	TTACTTAGGTACCCGGGG	pKT25-minD point mutations construction (vector)
F- 26bp(KpnI)-*minD*	CGACTCTAGAGGATCCCCGGGTACCTGCTGCACGC	pKT25-minD point mutations construction (insert)
R-minD-25bp(EcoRI)	acgttgtaaaacgacggccgaattcttaTCCTCCG	pKT25-minD point mutations construction (insert)
F-L194Rnew	/phos/CGTAGCATGGAAGATGTGCTGG	pKT25-minD^{L194R} construction
R-L194R	CATGTCACCTCTGCTTACG	pKT25-minD^{L194R} construction
F-MinD	GATCCCTTTTTAACAAGGAATTTCTCACGTCTTGAGCGATTGTG	MG1655 Δ*mimD* construction
R-MinD	GAAAGAAATCGAGTAATGCCATAACATATCCTCCTTAGTTCCTATT	MG1655 Δ*mimD* construction

B. Oligonucleotides used for real time PCR

Resource	Sequence	Source
F-16S	CGTGTTGTGAAATGTTGGGTTAA	(Baker; Eöry; Yakhnin; Mercante *et al.*, 2007)

R-16S	ACCGCTGGCAACAAAAGATAA	(Baker; Eöry; Yakhnin; Mercante *et al.*, 2007)
F-cheA	CAA TAT TAC CGG GCG ACA TC	This Work
R-cheA	GGC GAG ACT TCT ACT GTT TC	This Work
F-gadE	GAGAAATTAGATGCCGAGAG	(Segura; Auffret; Bibbal; Bertoni *et al.*, 2018)
R-gadE	TTGTGAATTCTTATGGGGCA	(Segura; Auffret; Bibbal; Bertoni *et al.*, 2018)
F-motA	CTTCCTCGGTTGTCGTCTGT	(Chen; Ali; Wu; Liu *et al.*, 2018)
R-motA	CTATCGCCGTTGAGTTTGGT	(Chen; Ali; Wu; Liu *et al.*, 2018)
F-adhE	AACCTGTGGTGTTCTGTCTG	This work
R-adhE	GCAGTTGAAGTCGGGTTAGT	This work
F-fabB	TGGCGTACAGGAAGCTATCTA	This work
R-fabB	CTCGTCCAGCTCTTCAATGTT	This work
F-gyrA	GCGTGCGTGATGGTCTGTAC	(Bury-Moné; Nomane; Reymond; Barbet *et al.*, 2009)
R-gyrA	CGTGCTCAAGACCGGTCAGT	(Bury-Moné; Nomane; Reymond; Barbet *et al.*, 2009)
F-9S	TTTGGCGGATGAGAGAAGAT	This work
R-9S	CGCCAGGCAAATTCTGTTT	This work

References

1. Baker CS, Eöry LA, Yakhnin H, Mercante J, Romeo T, Babitzke P. 2007. Csra inhibits translation initiation of escherichia coli hfq by binding to a single site overlapping the shine-dalgarno sequence. J Bacteriol. 189(15):5472-5481.
2. Bury-Moné S, Nomane Y, Reymond N, Barbet R, Jacquet E, Imbeaud S, Jacq A, Bouloc P. 2009. Global analysis of extracytoplasmic stress signaling in escherichia coli. PLoS Genet. 5(9):e1000651.
3. Chen XP, Ali L, Wu LY, Liu C, Gang CX, Huang QF, Ruan JH, Bao SY, Rao YP, Yu D. 2018. Biofilm formation plays a role in the formation of multidrug-resistant. Front Microbiol. 9:367.
4. Segura A, Auffret P, Bibbal D, Bertoni M, Durand A, Jubelin G, Kérourédan M, Brugère H, Bertin Y, Forano E. 2018. Factors involved in the persistence of a shiga toxin-producing. Front Microbiol. 9:375.
