# Supplementary material for: MinD-RNase E interplay controls localization of polar mRNAs in E. coli
Source: EMBO J. 2024 Jan 19;43(4):8. doi: 10.1038/s44318-023-00026-9 (PMC10897333; doi:10.1038/s44318-023-00026-9)
Supplement: Supplementary file 4 — Appendix [file 44318_2023_26_MOESM4_ESM.pdf]

MinD-RNase E interplay controls localization of polar mRNAs in *E. coli*

Shanmugapriya Kannaiah[1,2*x], Omer Goldberger[1*], Nawsad Alam[1,3], Georgina Barnabas[4,5], Yair Pozniak[4], Anat Nussbaum-Shochat[1], Ora Schueler-Furman[1], Tamar Geiger[4,6] and Orna Amster-Choder[1x]

[1]Department of Microbiology and Molecular Genetics, IMRIC, The Hebrew University Faculty of Medicine, P.O.Box 12272, Jerusalem 91120, Israel

[2]Current address: Department of Molecular Microbiology, Washington University School of Medicine, St Louis, MO 63110, USA,

[3]Current address: Department of Biochemistry, University of Oxford, Oxford, OX1 3QU, UK

[4]Department of Human Molecular Genetics and Biochemistry, Sackler School of Medicine, Tel Aviv University, Tel-Aviv 6997801, Israel

[5]Current address: Department of Pathology, University of British Columbia, Vancouver, British Columbia V6T 1Z4, Canada

[6]Current address: Department of Molecular Cell Biology, Weizmann Institute of Science, Rehovot, 76100001, Israel

* These authors contributed equally to the manuscript

[x] **Corresponding Authors:** Orna Amster-Choder (ornaam@ekmd.huji.ac.il) and Shanmugapriya Kannaiah (kannaiah@wustl.edu)

TABLE OF CONTENTS

Appendix Figure S1. Validation that 42°C restrict the activity of RNase E temperature-sensitive mutant (rne-3071) (related to Fig. 2) , page 2
AppendixFigure S2. Specificity of the GFP-TRAP immunoprecipitation procedure (related to Fig. 3), page 3
Appendix Figure S3. Expression of RNase E in wild type and ΔminCDE cells in the different growth phases (related to Fig. 4), page 4
Appendix Figure S4. Structural disorder of the RNase E segment 378-724 (related to Figure 5), page 5
Appendix Figure S5. MinD purification validation by Western blot analysis (related to Figure 5), page 6
Appendix Fig. S6. The full gel used for the Far-Western analysis and validation of protein quantity by Coomassie staining (related to Figure 5) page 7
Appendix Fig. S7. MinD mutations do not cause reduction in protein level (related to Fig. 5), page 8

Appendix Fig. S1. Validation that 42°C restrict the activity of RNase E temperature-sensitive mutant (*rne-3071)*

(related to Fig. 2)

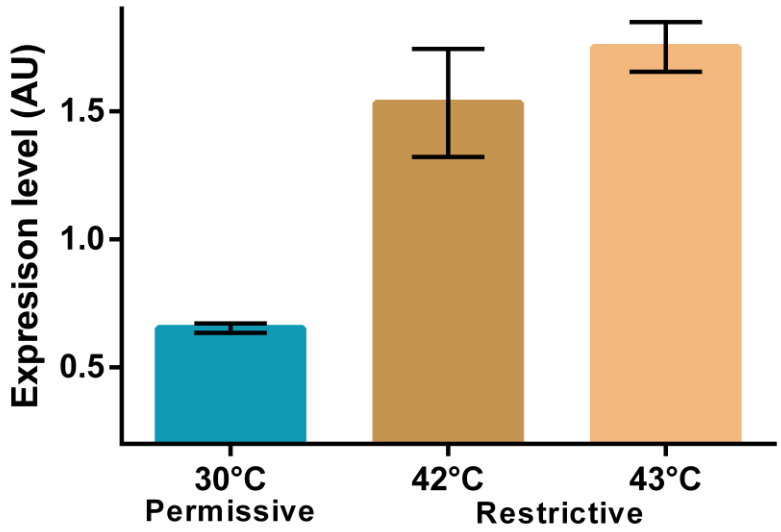

Bar plot showing the expression level of 9S rRNA in the permissive (30°C) or restrictive (42°C or 43°C) temperatures in *rne307* cells that express a temperature-sensitive mutant of RNase E. Expression level was measured by qPCR and SEM error bars are presented for each sample.

Accumulation of the 9S rRNA precursor of p5S in both 42°C or 43°C indicates that RNase E is inactive in these temperatures. Data are the mean of biological triplicates.

Appendix Fig. S2. Specificity of the GFP-TRAP immunoprecipitation procedure (related to Fig. 3)

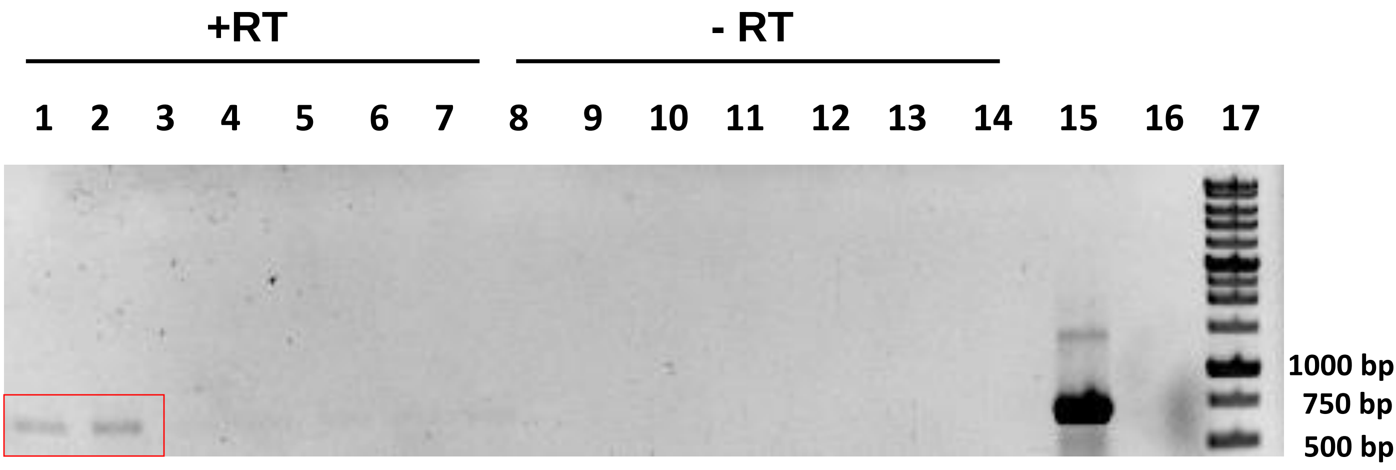

Agarose gel showing RT-PCR products. RNA was isolated from the GFP-TRAP immunoprecipitated eluate and analyzed by RT-PCR using *bglG* primers. The numbers on top represent the following: 1& 8: *bglG*-6xbs eluate 1; 2 & 9: *bglG*-6xbs eluate 2; 3 & 10: *bglG* eluate 1; 4 & 11: *bglG* eluate 2; 5 & 12: flowthrough *bglG*-6xbs; 6 & 13: flowthrough *bglG*; 7 & 14: cell lysate *bglG*. 1-7: samples treated with reverse transcriptase (+RT); 8-14: samples not treated with reverse transcriptase (-RT); 15: positive control; 16: negative control; and 17: 1kb ladder. The amplicon size is 642 bp.

Appendix Fig. S3. Expression of RNase E in wild type and Δ*minCDE* cells in the different growth phases (related to Fig. 4)

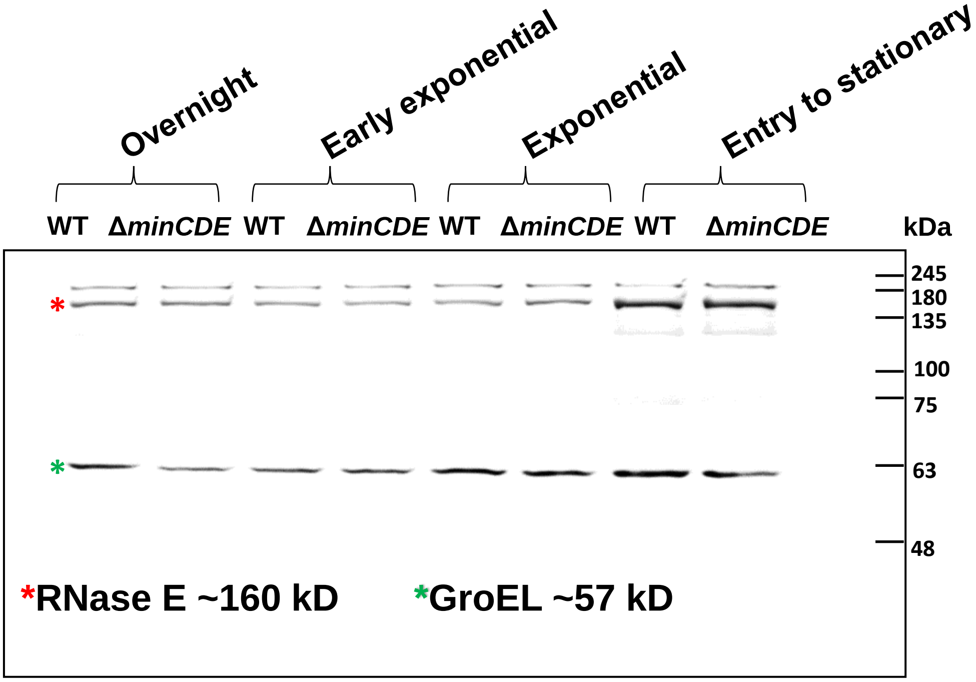

Representative Western blot image of RNase E expression at different growth phases in wild type and Δ*minCDE* cells. GroEL expression was used for normalization.

Appendix Fig. S4. Structural disorder of the RNase E segment 378-724 (related to Figure 5**)**

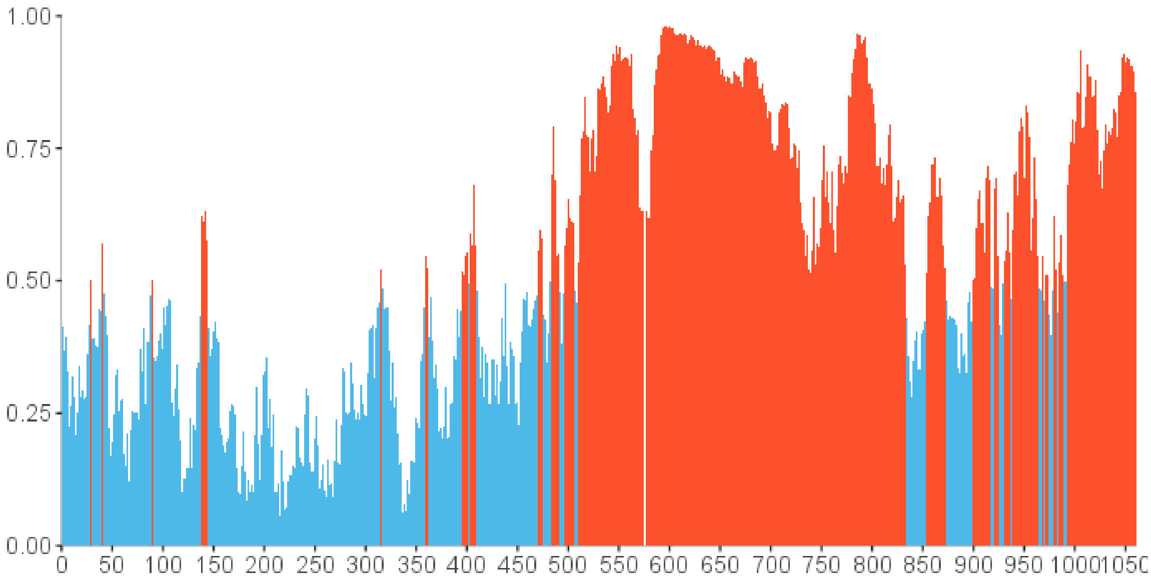

IUPred predicted disorder in RNase E. X-axis=disorder probability; Y-axis=residue position. Disordered regions (disorder probability >0.5) are shaded in red).

Appendix Fig. S5. MinD purification validation by Western blot analysis (related to Figure 5)

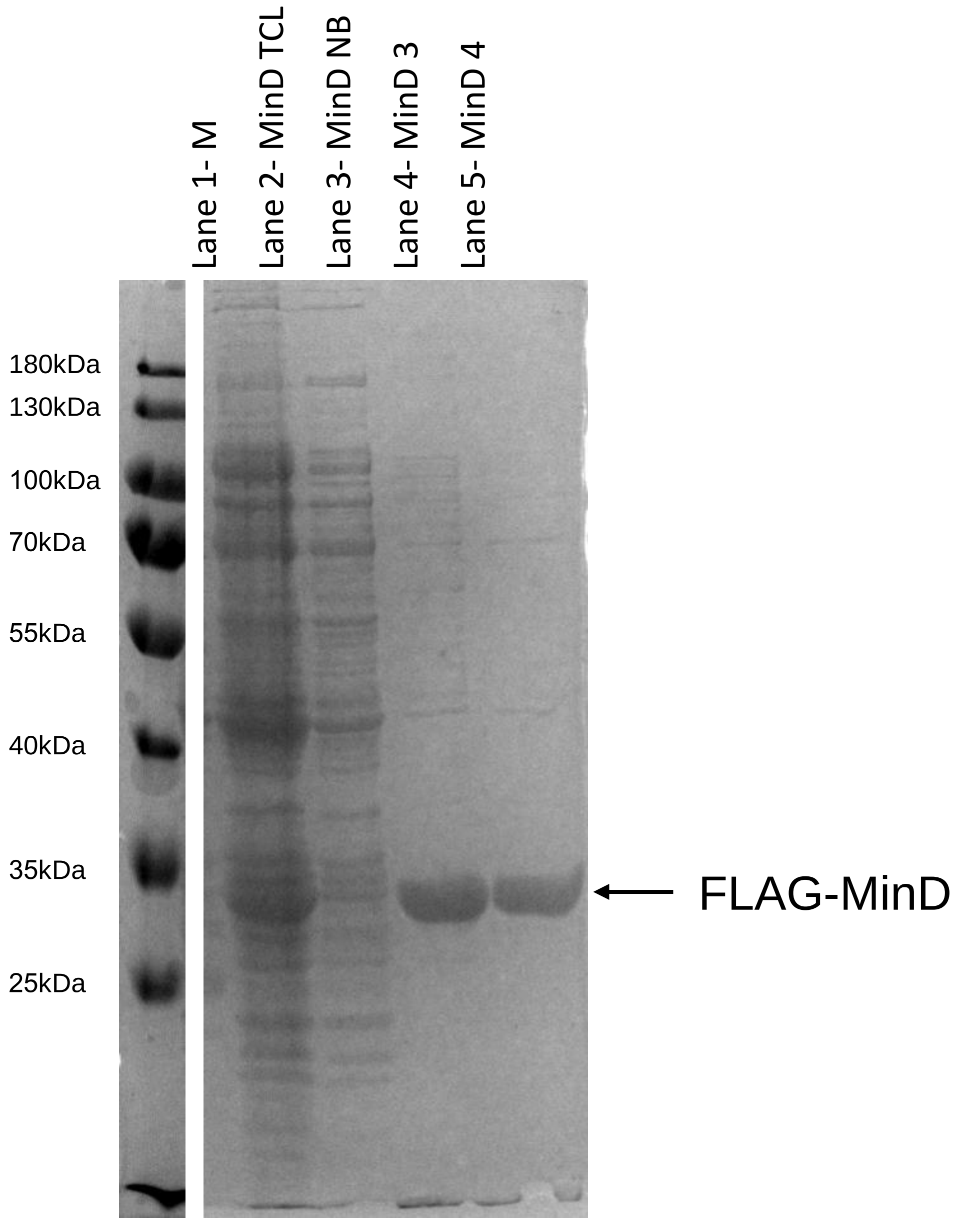

Western blot analysis showing MinD purification. Lane 1, size markers (M); lane 2, total cell lysate (TLC); lane 3, unbound (NB); lane 4, eluted fraction 3; lane 5, eluted fraction 4. Eluted fraction 3 was used for the Far-Western analysis.

Appendix Fig. S6. The full gel used for the Far-Western analysis and validation of protein quantity by Coomassie staining (related to Figure 5)

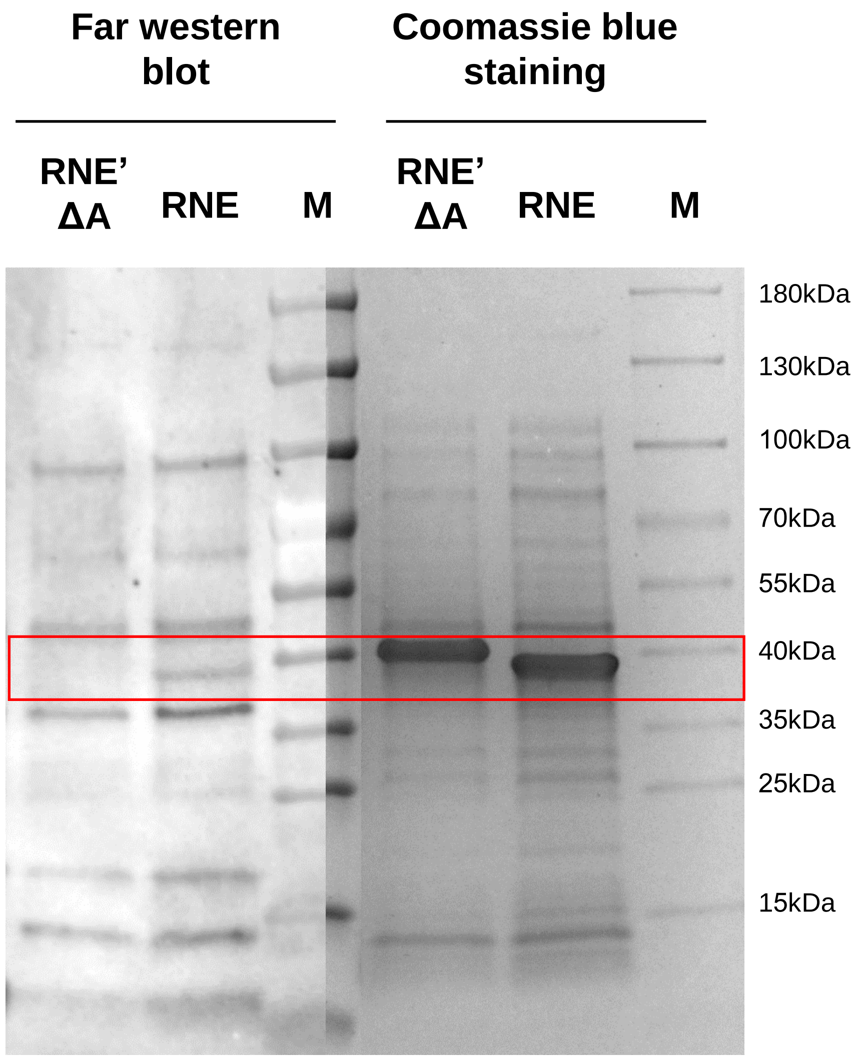

For the Far-Western analysis, equal amounts of each sample were run twice on an SDA-polyacrylamide gel. The gel was subsequently cut to two parts, each containing one set of the samples. One part was used for Far-Western and the second for Coomassie blue staining.

Left panel: Shown are the full-length lanes that were subjected to Far-Western, whose relevant parts are presented in **Fig 4E.** See details of the analysis in the legend to **Fig. 4E.**

Right panel: Shown is the half gel that was stained with Coomassie blue.

Appendix Fig. S7. The MinD mutations used in the 2-hybrid assay do not cause reduction in protein level (related to Fig. 5)

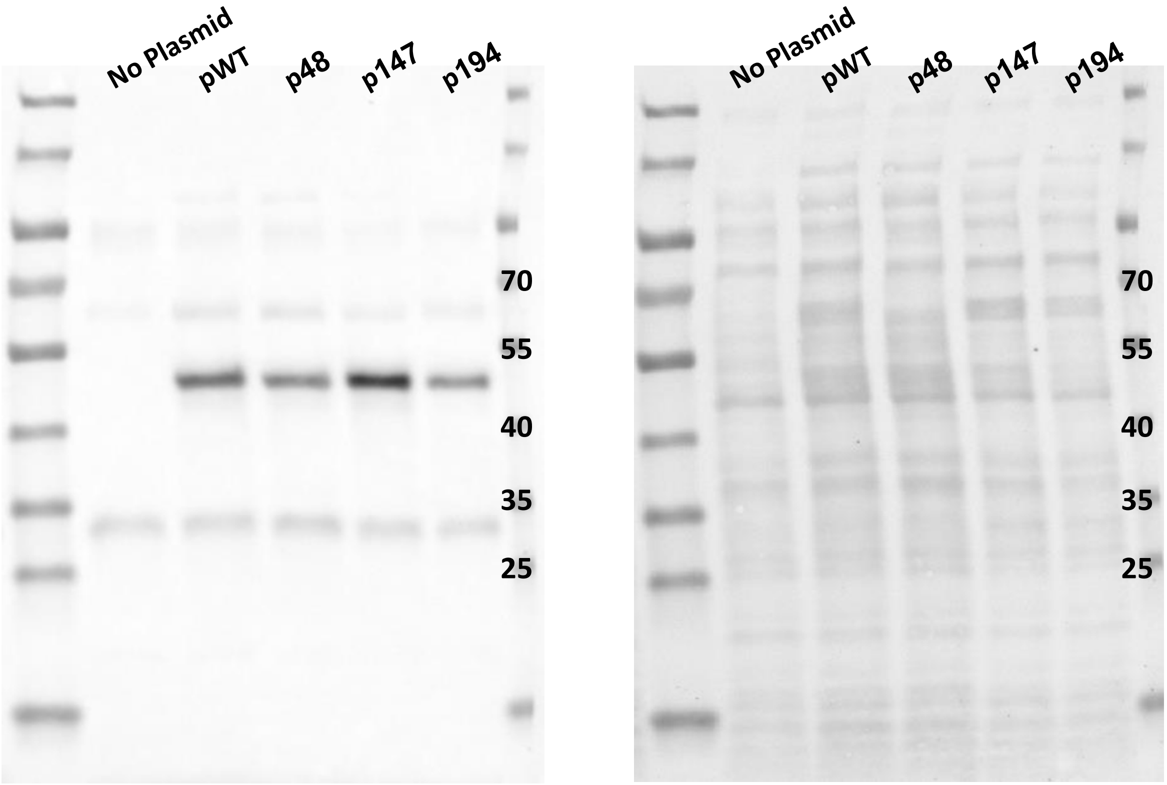

Western blot analysis detecting the expression of wild type MinD and its mutants from pKT25-minD and its derivatives in BTH101 cells also expressing RNA’ from a pUT18C (the same as in the bacterial two hybrid experiment) (left panel). Equal number of cells were sampled and blotted onto the membrane, as can be seen in the Ponceau S staining of the membrane before probing (right panel). The membrane was probed with anti-MinD antiserum.
