# Supplementary material for: MinD-RNase E interplay controls localization of polar mRNAs in E. coli
Source: EMBO J. 2024 Jan 19;43(4):8. doi: 10.1038/s44318-023-00026-9 (PMC10897333; doi:10.1038/s44318-023-00026-9)
Supplement: Supplementary file 5 — Movie EV1 [file 44318_2023_26_MOESM5_ESM.zip › MovieEV1_legend.docx]

**Movie EV1. RNase E forms transient polar clusters in *∆minCDE* cells**

Polar accumulation and disassembly of RNase E-YFP expressed from its own promoter was monitored in *∆minCDE* cells by time-lapse microscopy. Images were acquired every 30 sec for a total of 5 minutes.
