# Supplementary material for: MinD-RNase E interplay controls localization of polar mRNAs in E. coli
Source: EMBO J. 2024 Jan 19;43(4):8. doi: 10.1038/s44318-023-00026-9 (PMC10897333; doi:10.1038/s44318-023-00026-9)
Supplement: Supplementary file 6 — Movie EV2 [file 44318_2023_26_MOESM6_ESM.zip › MovieEV2_legend.docx]

**Movie EV2. RNase E movement does not correlate with MinD oscillation**

In order to check the correlation between RNase E and MinD dynamics, RNaseE-mCherry and mGFP-MinD were co-expressed, and snapshots were taken every 30 sec for a span of 5 minutes.
