# Supplementary figures and images for: MinD-RNase E interplay controls localization of polar mRNAs in E. coli
Source: EMBO J. 2024 Jan 19;43(4):8. doi: 10.1038/s44318-023-00026-9 (PMC10897333; doi:10.1038/s44318-023-00026-9)

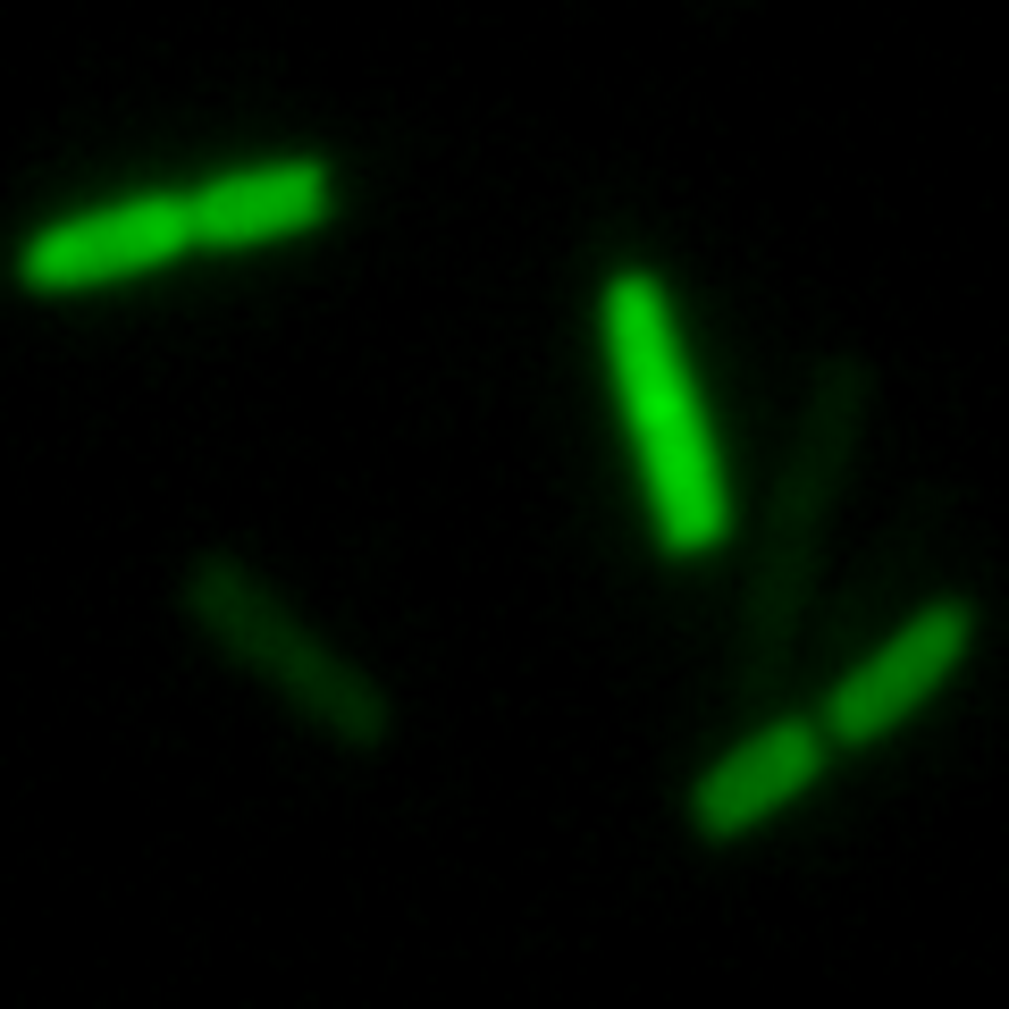

Supplement: Supplementary file 7 — Source Data Fig. 1 [file 44318_2023_26_MOESM7_ESM.zip › Figure 1/1A/6xbs.tif]

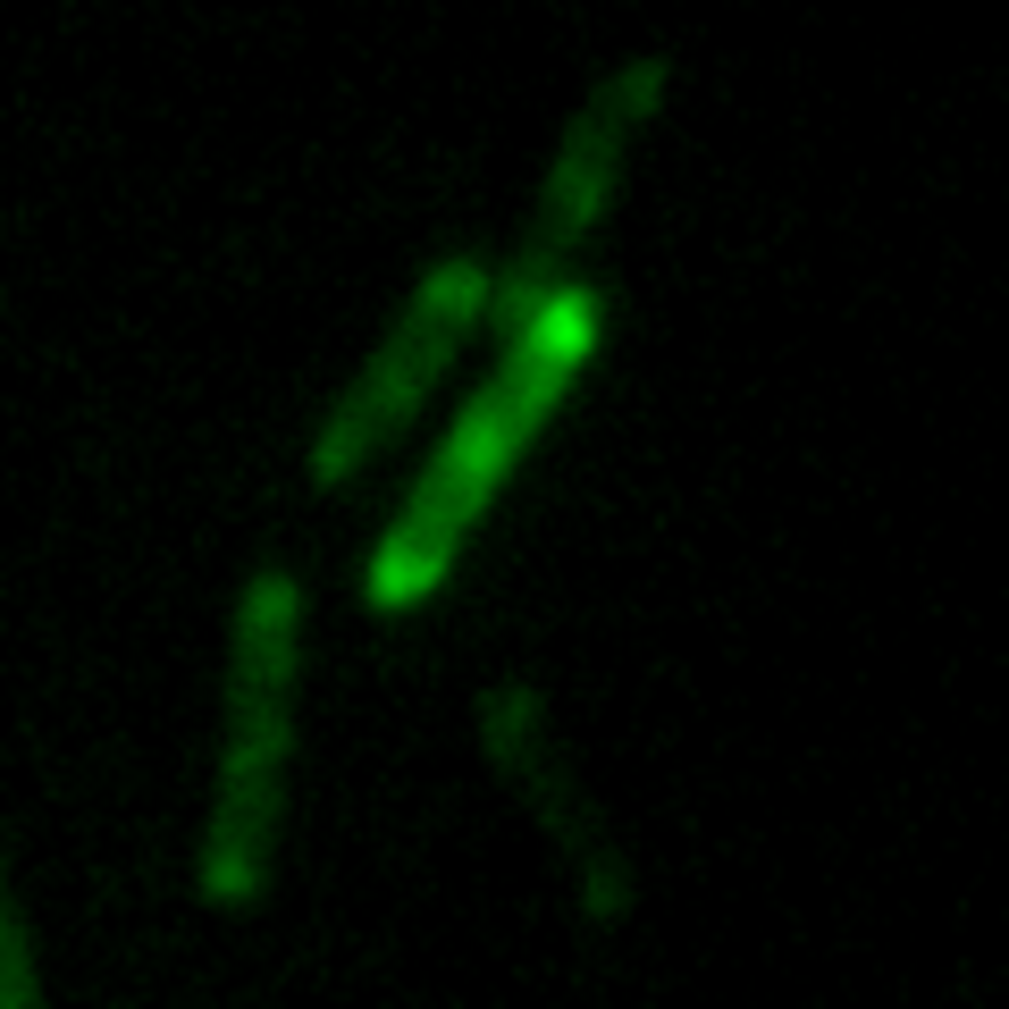

Supplement: Supplementary file 7 — Source Data Fig. 1 [file 44318_2023_26_MOESM7_ESM.zip › Figure 1/1A/cheA.tif]

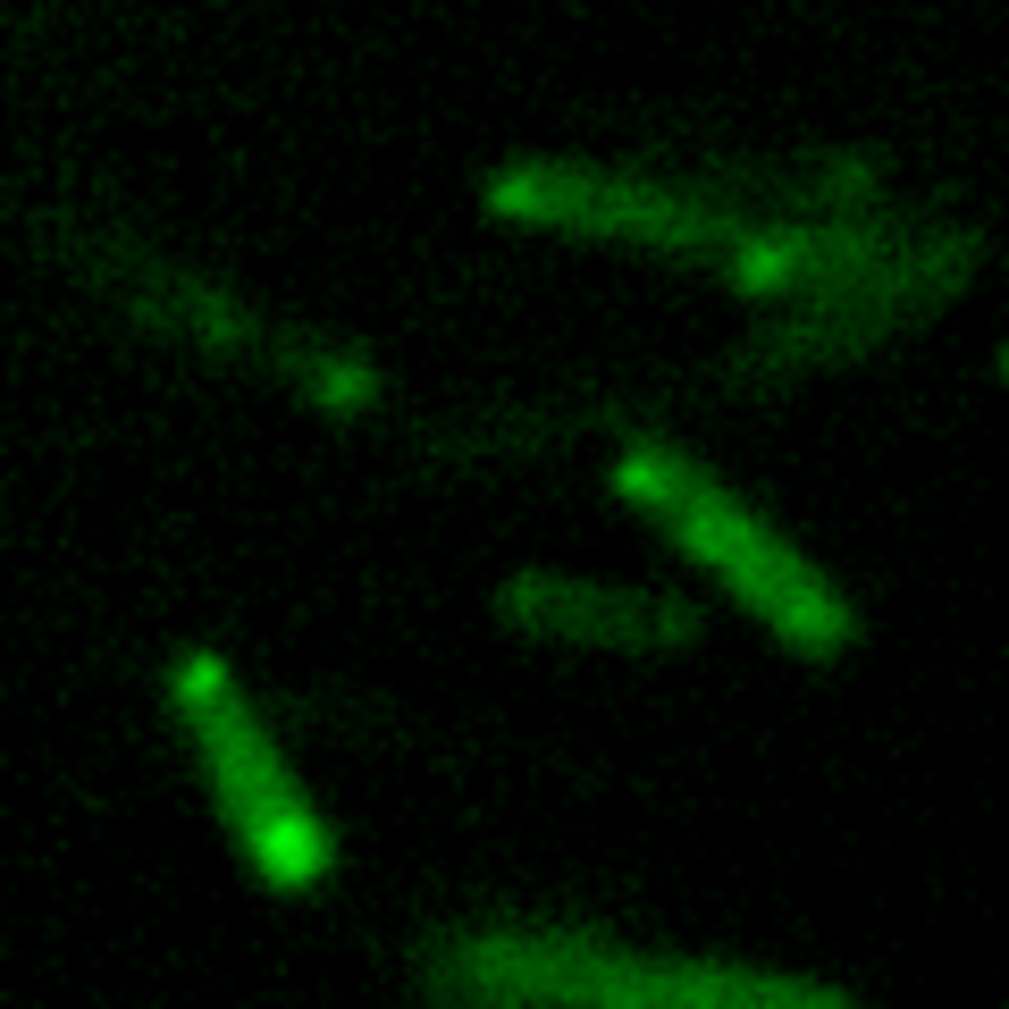

Supplement: Supplementary file 7 — Source Data Fig. 1 [file 44318_2023_26_MOESM7_ESM.zip › Figure 1/1A/bglG.tif]

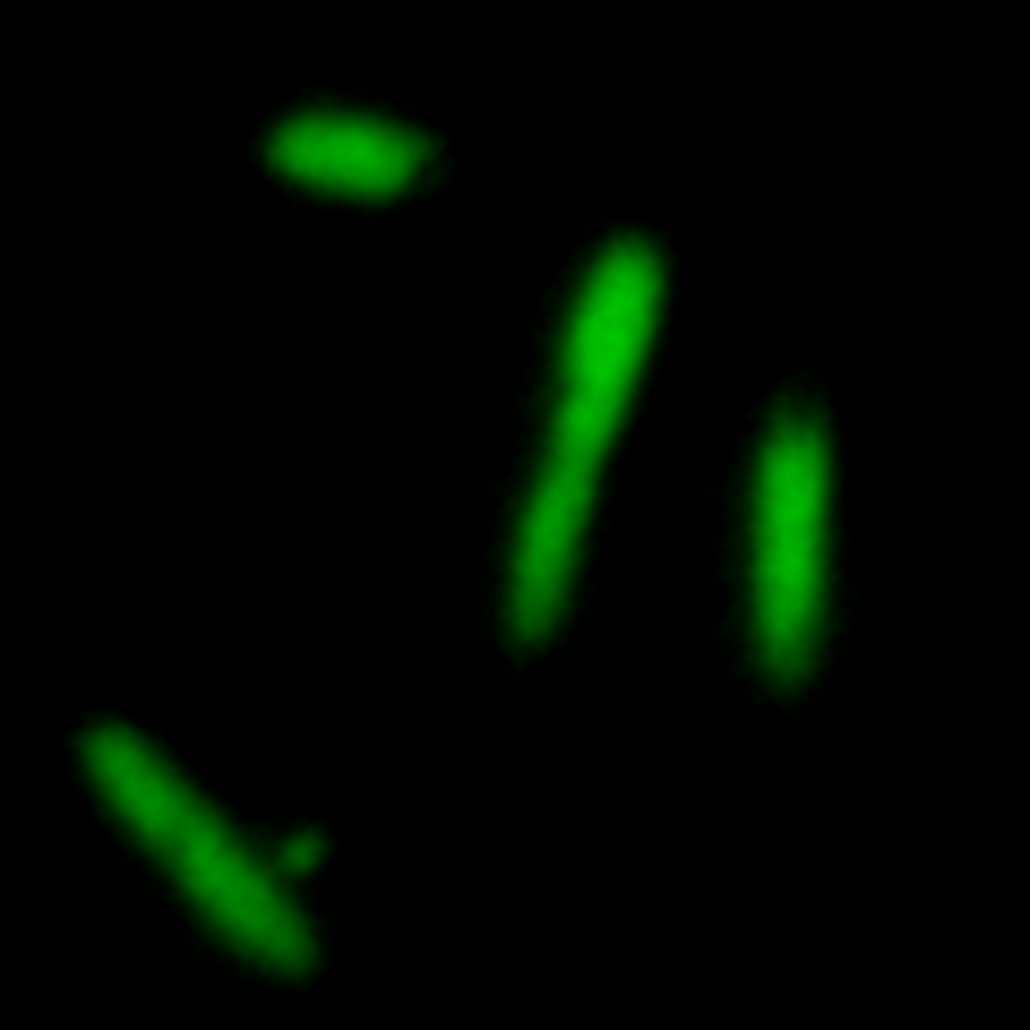

Supplement: Supplementary file 7 — Source Data Fig. 1 [file 44318_2023_26_MOESM7_ESM.zip › Figure 1/1B/6xbs.tif]

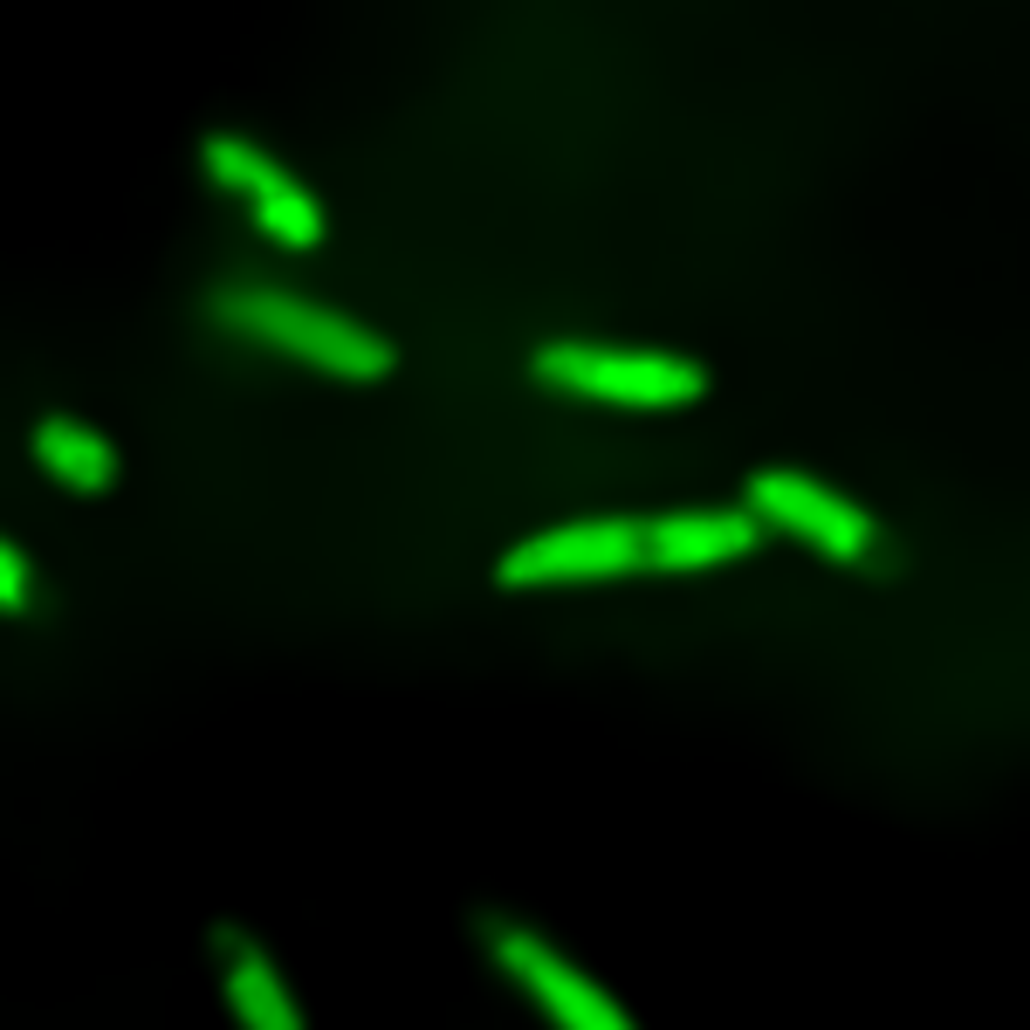

Supplement: Supplementary file 7 — Source Data Fig. 1 [file 44318_2023_26_MOESM7_ESM.zip › Figure 1/1B/cheA.tif]

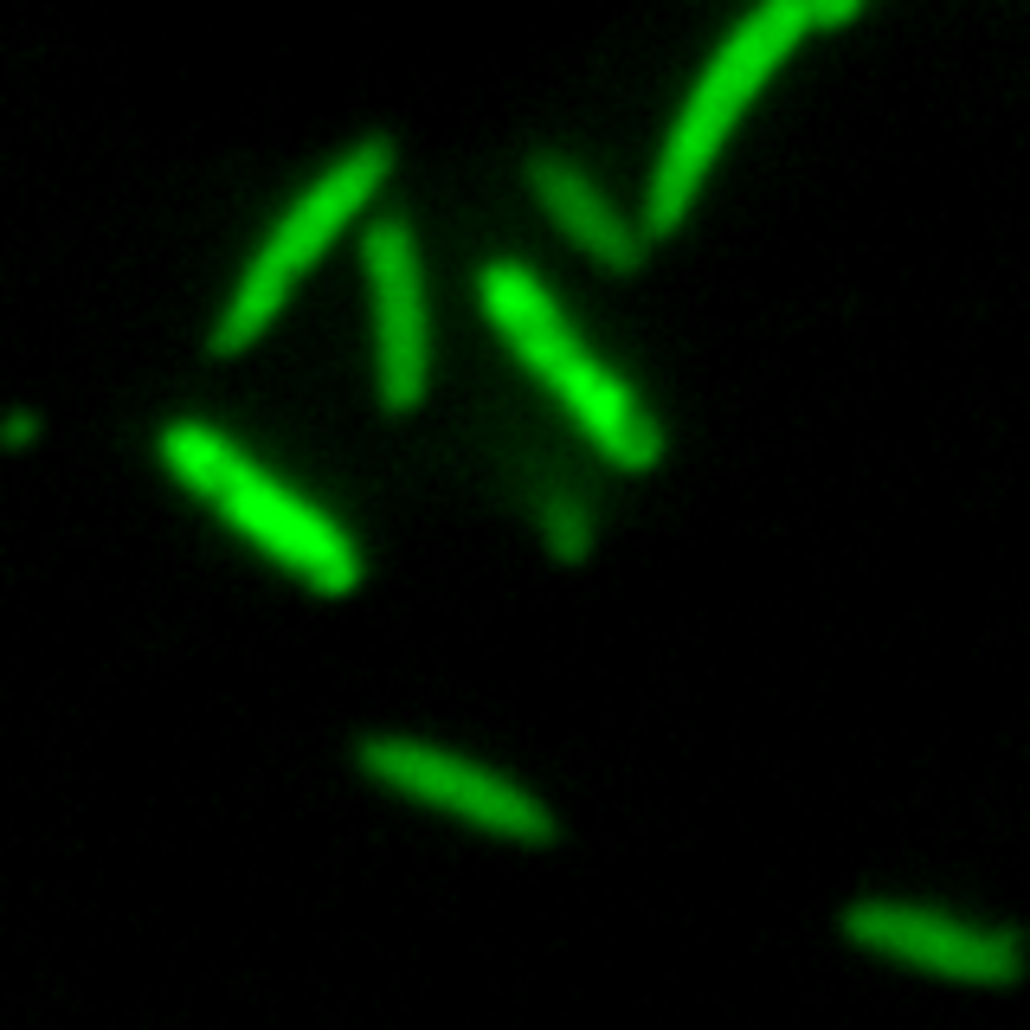

Supplement: Supplementary file 7 — Source Data Fig. 1 [file 44318_2023_26_MOESM7_ESM.zip › Figure 1/1B/bglG.tif]

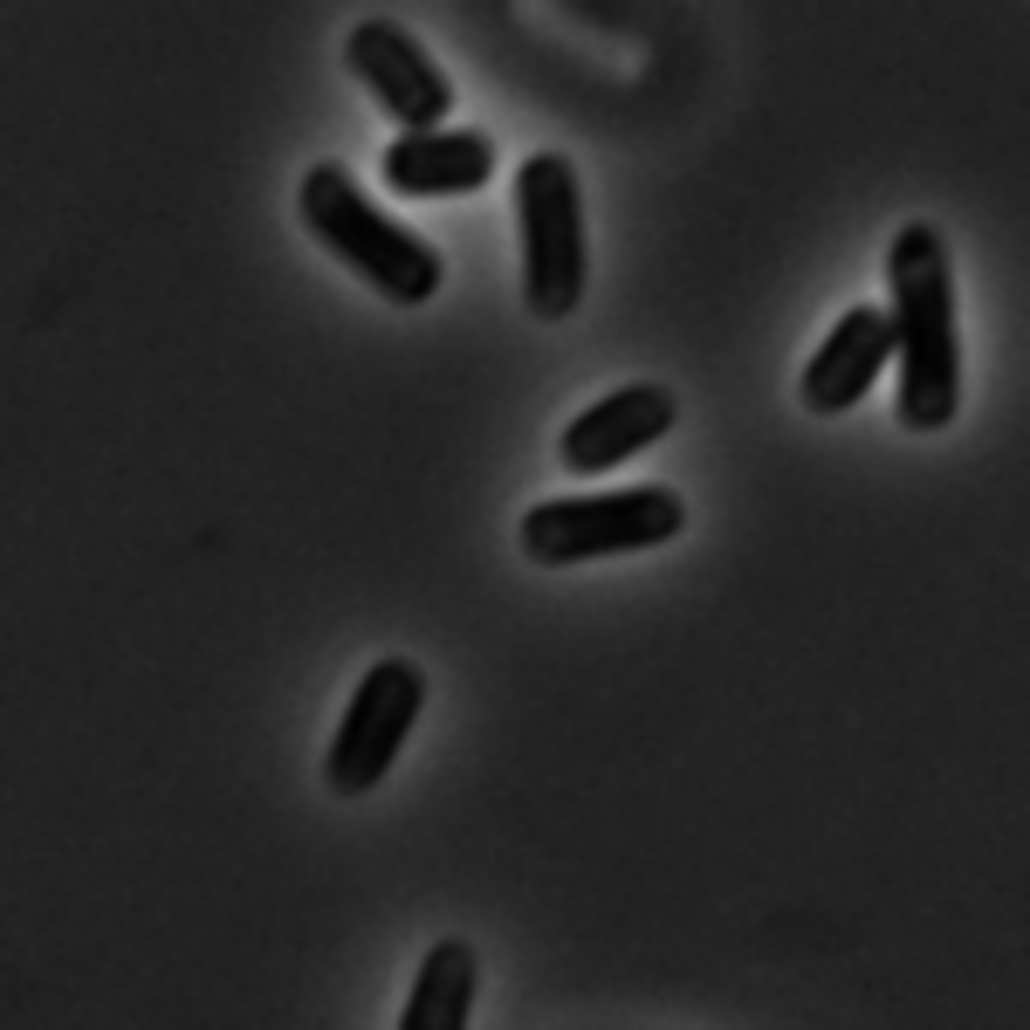

Supplement: Supplementary file 8 — Source Data Fig. 2 [file 44318_2023_26_MOESM8_ESM.zip › Figure 2/2A/bglG wt 42'C2.tif]

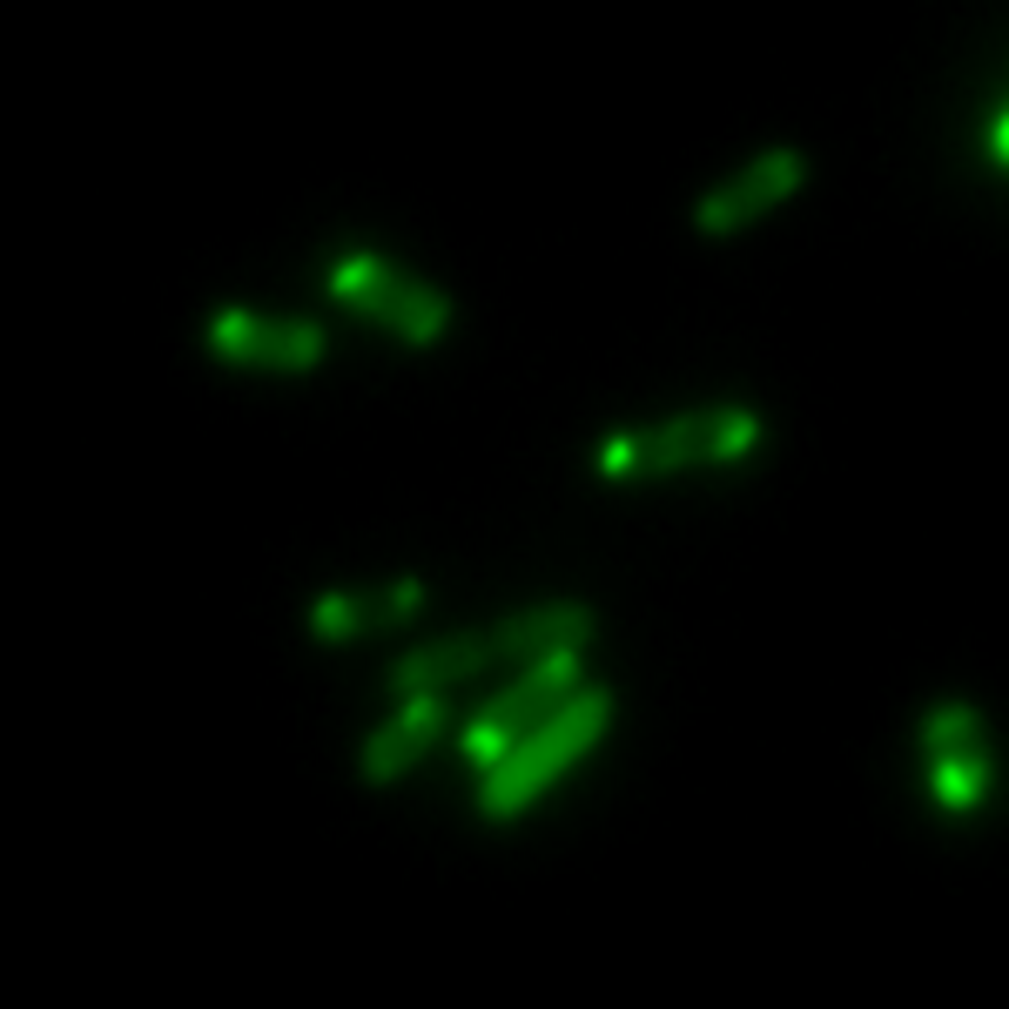

Supplement: Supplementary file 8 — Source Data Fig. 2 [file 44318_2023_26_MOESM8_ESM.zip › Figure 2/2A/cheA wt 42'C1.tif]

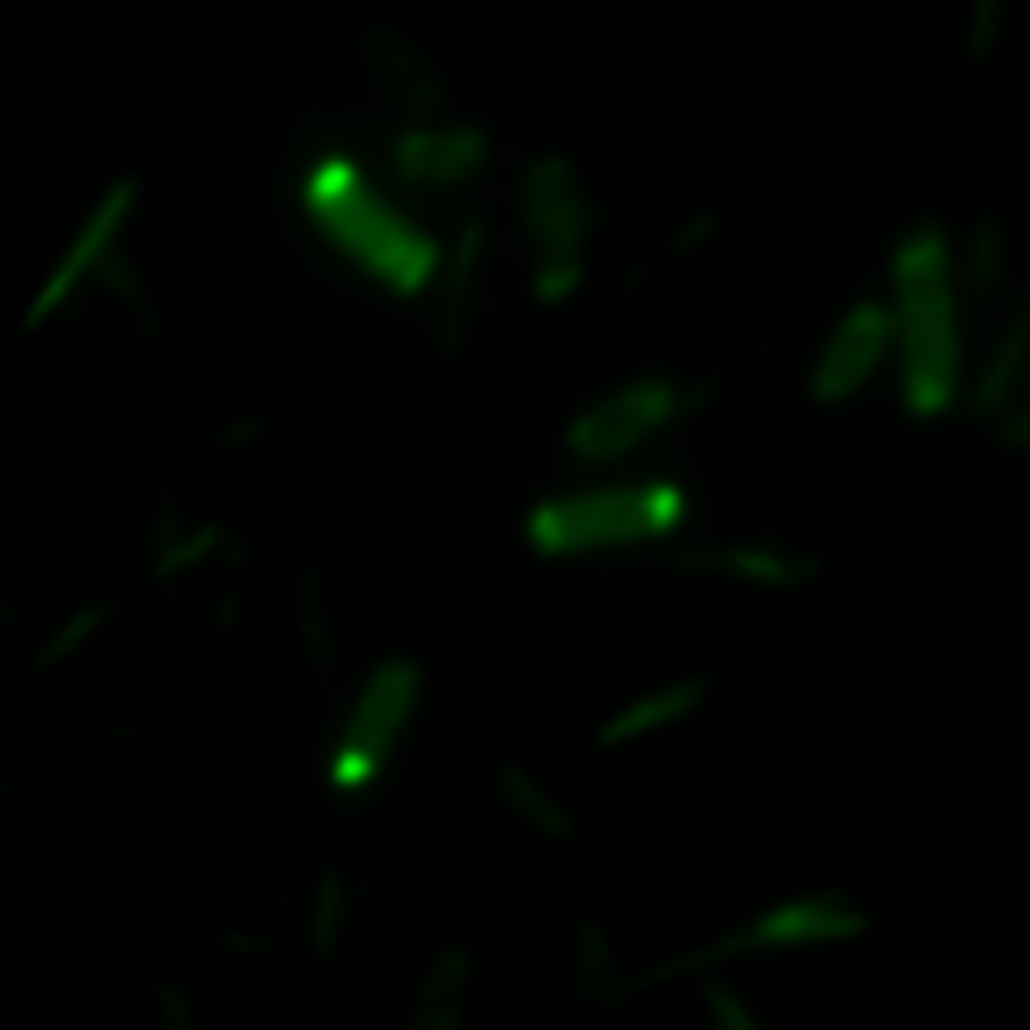

Supplement: Supplementary file 8 — Source Data Fig. 2 [file 44318_2023_26_MOESM8_ESM.zip › Figure 2/2A/bglG wt 42'C1.tif]

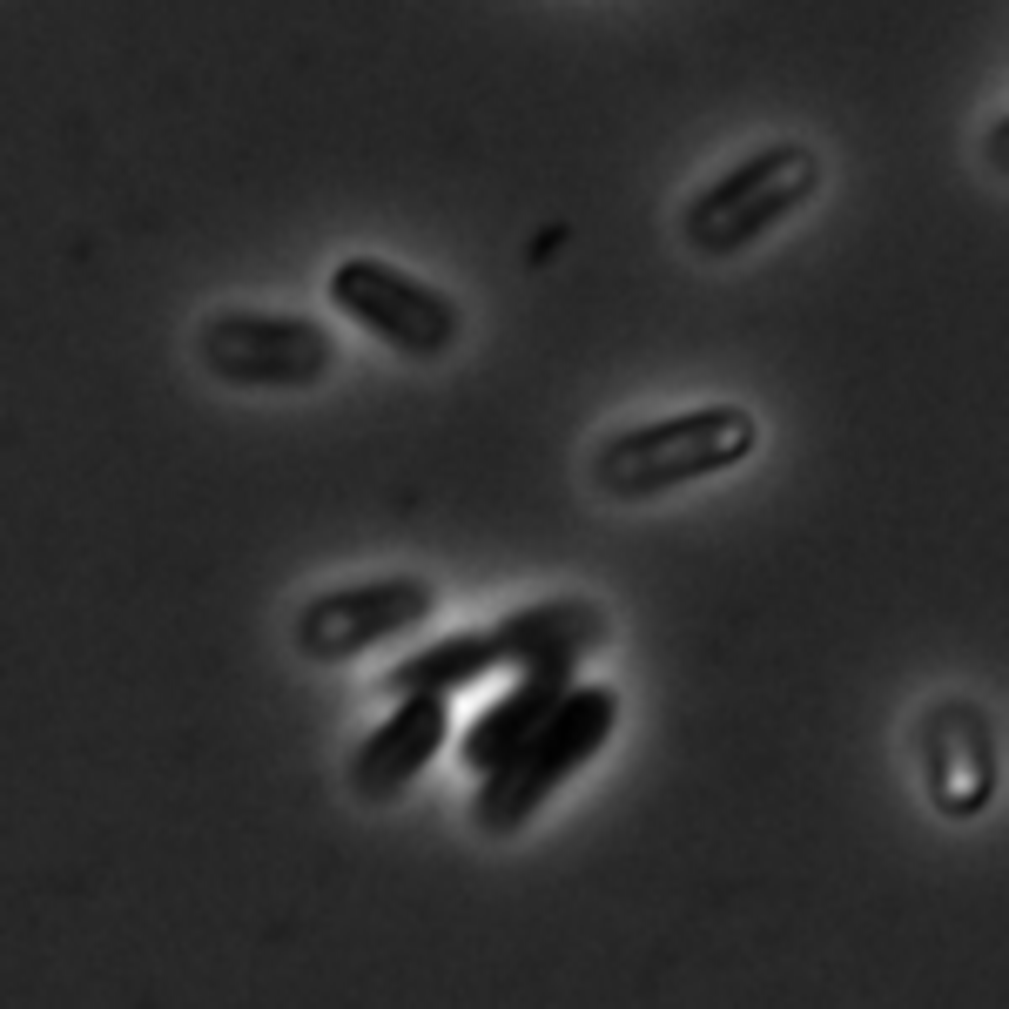

Supplement: Supplementary file 8 — Source Data Fig. 2 [file 44318_2023_26_MOESM8_ESM.zip › Figure 2/2A/cheA wt 42'C2.tif]

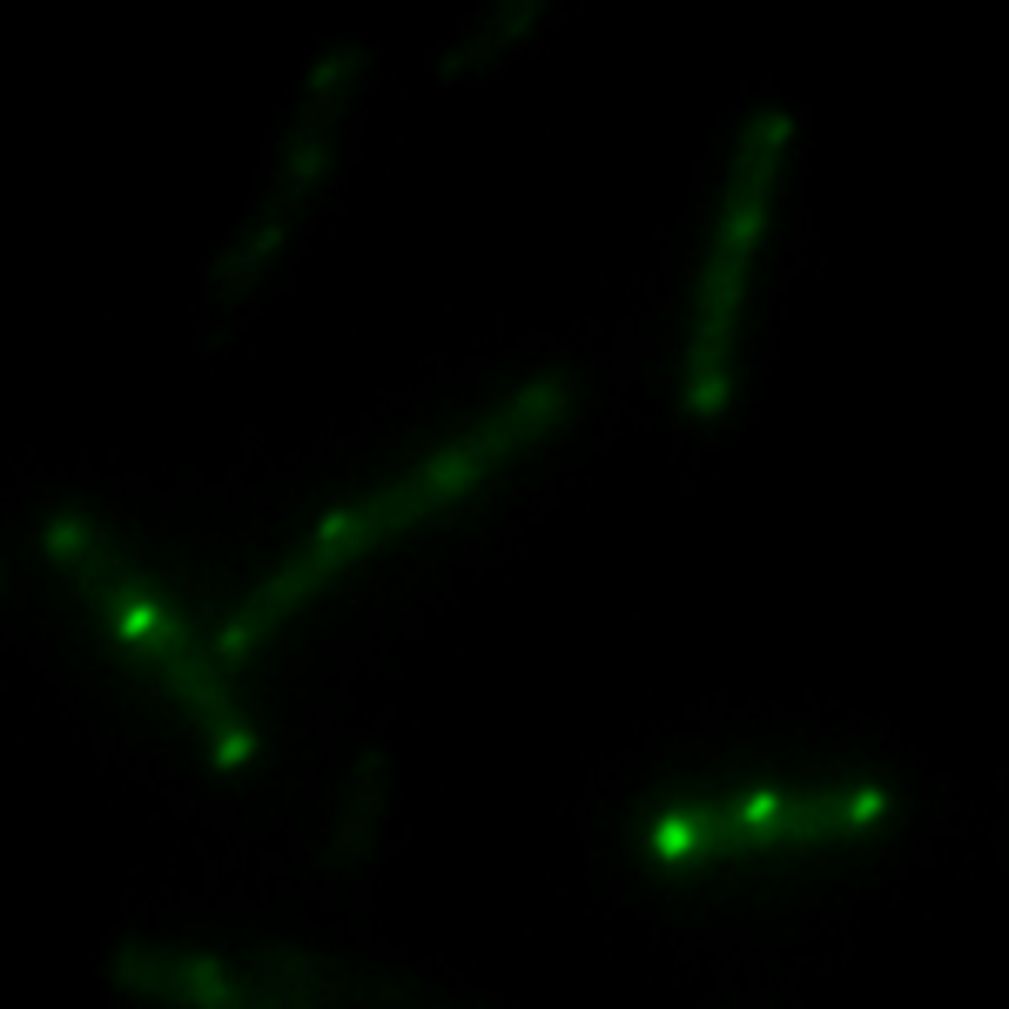

Supplement: Supplementary file 8 — Source Data Fig. 2 [file 44318_2023_26_MOESM8_ESM.zip › Figure 2/2A/cheA ts 30'C1.tif]

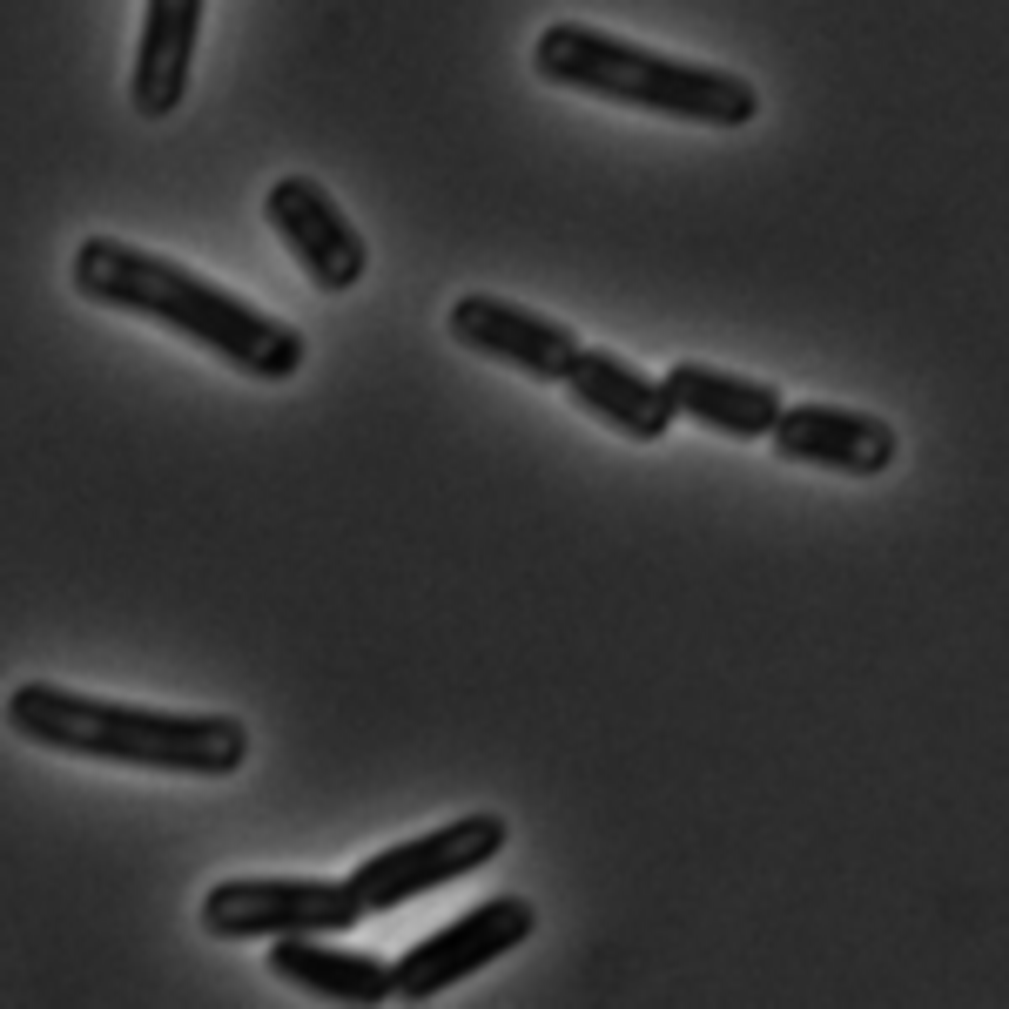

Supplement: Supplementary file 8 — Source Data Fig. 2 [file 44318_2023_26_MOESM8_ESM.zip › Figure 2/2A/bglG ts 30'C2.tif]

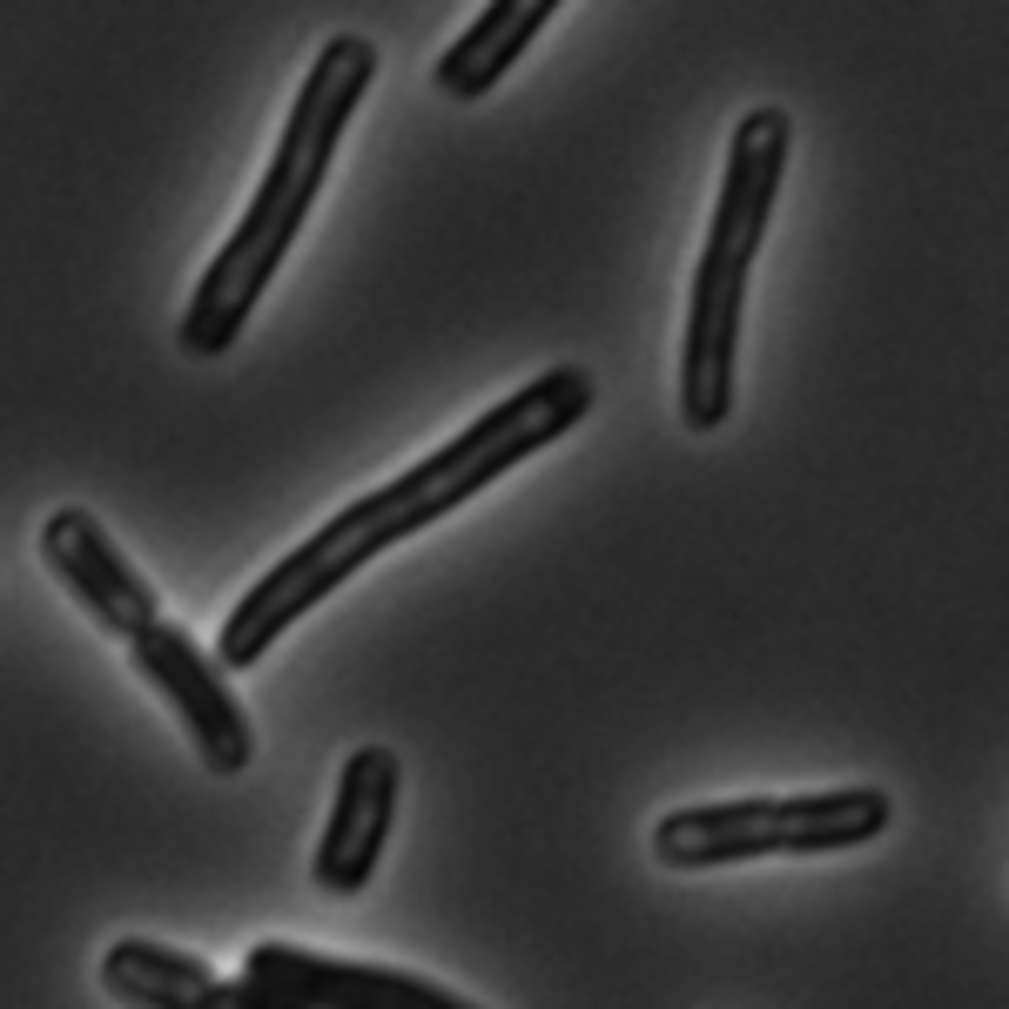

Supplement: Supplementary file 8 — Source Data Fig. 2 [file 44318_2023_26_MOESM8_ESM.zip › Figure 2/2A/cheA ts 30'C2.tif]

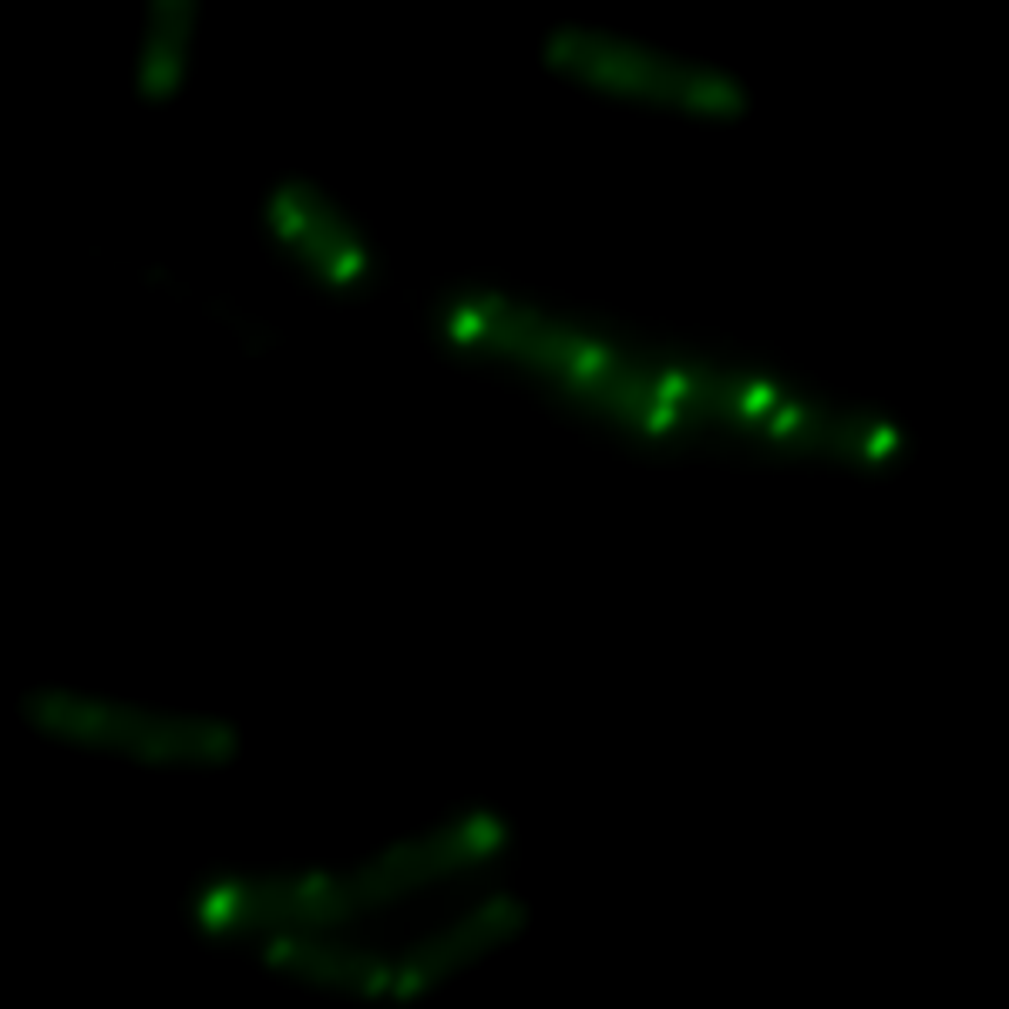

Supplement: Supplementary file 8 — Source Data Fig. 2 [file 44318_2023_26_MOESM8_ESM.zip › Figure 2/2A/bglG ts 30'C1.tif]

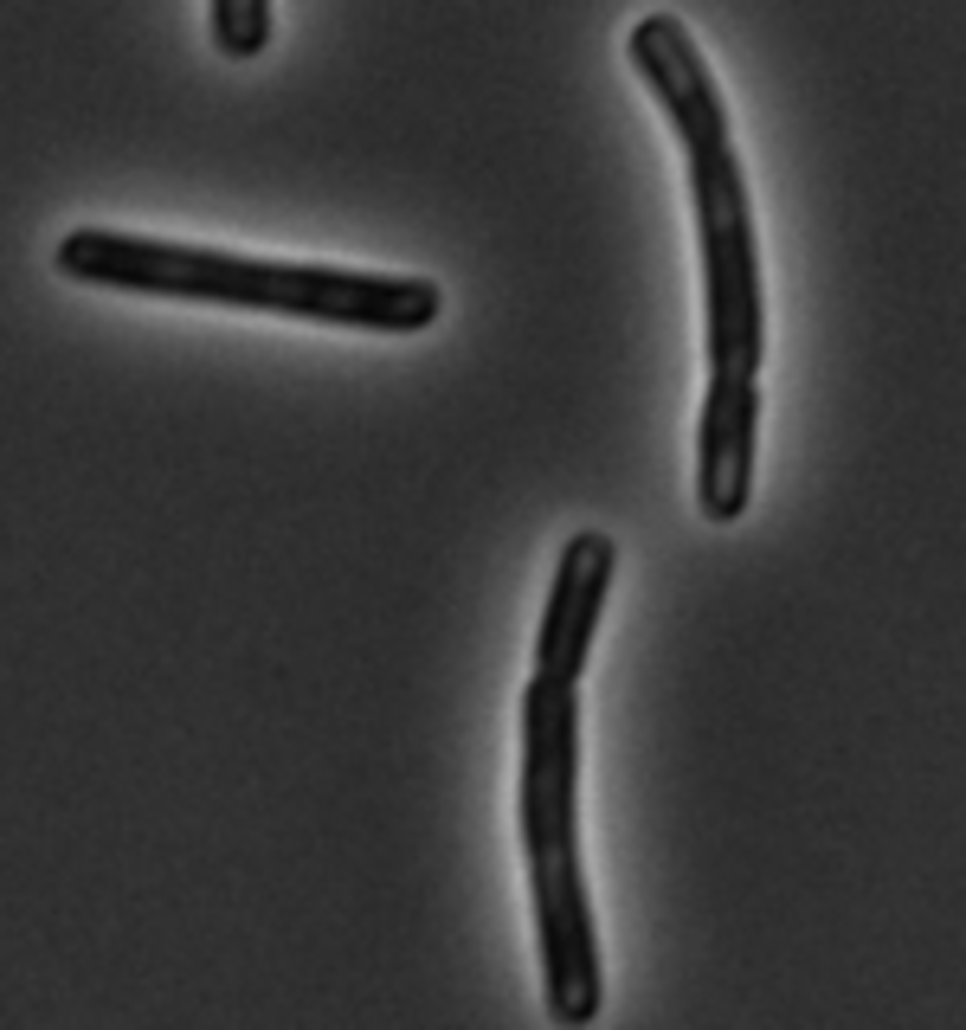

Supplement: Supplementary file 8 — Source Data Fig. 2 [file 44318_2023_26_MOESM8_ESM.zip › Figure 2/2A/bglG ts 42'C2.tif]

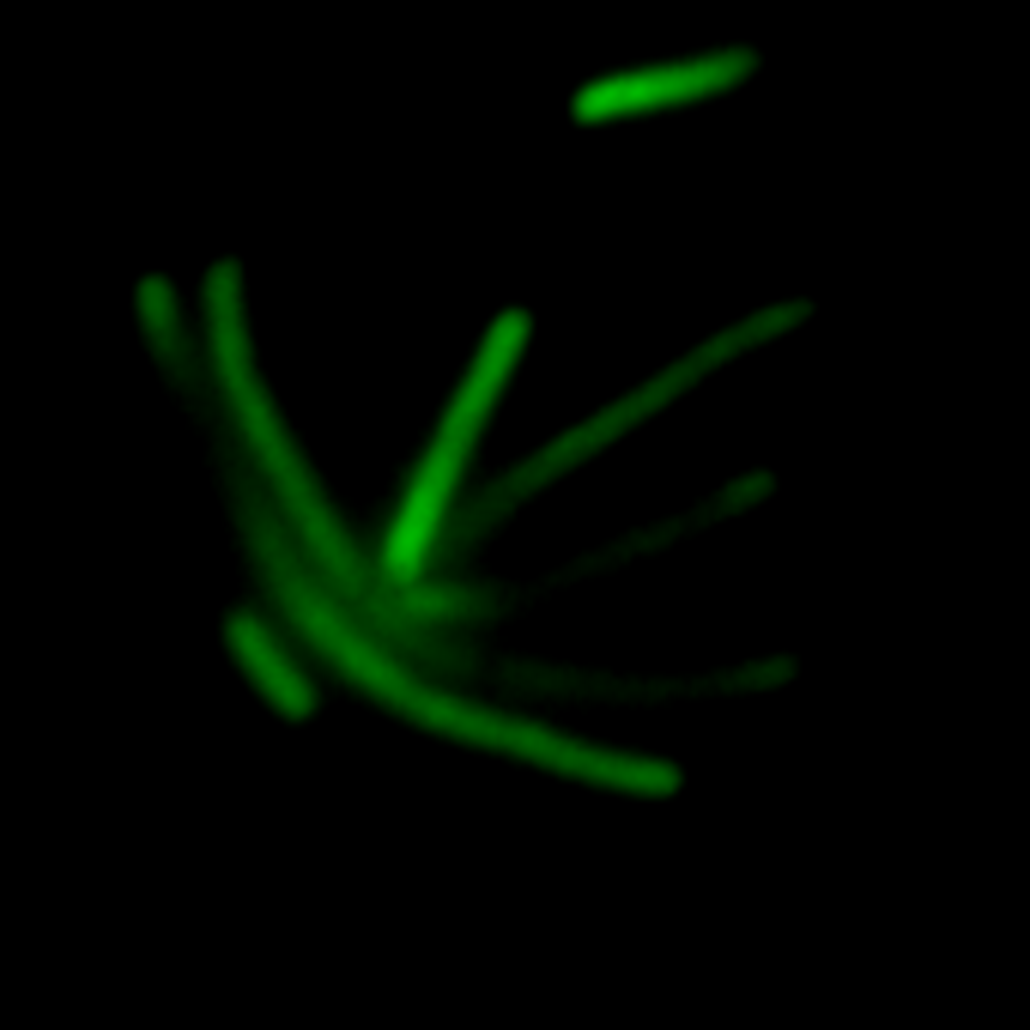

Supplement: Supplementary file 8 — Source Data Fig. 2 [file 44318_2023_26_MOESM8_ESM.zip › Figure 2/2A/cheA ts 42'C1.tif]

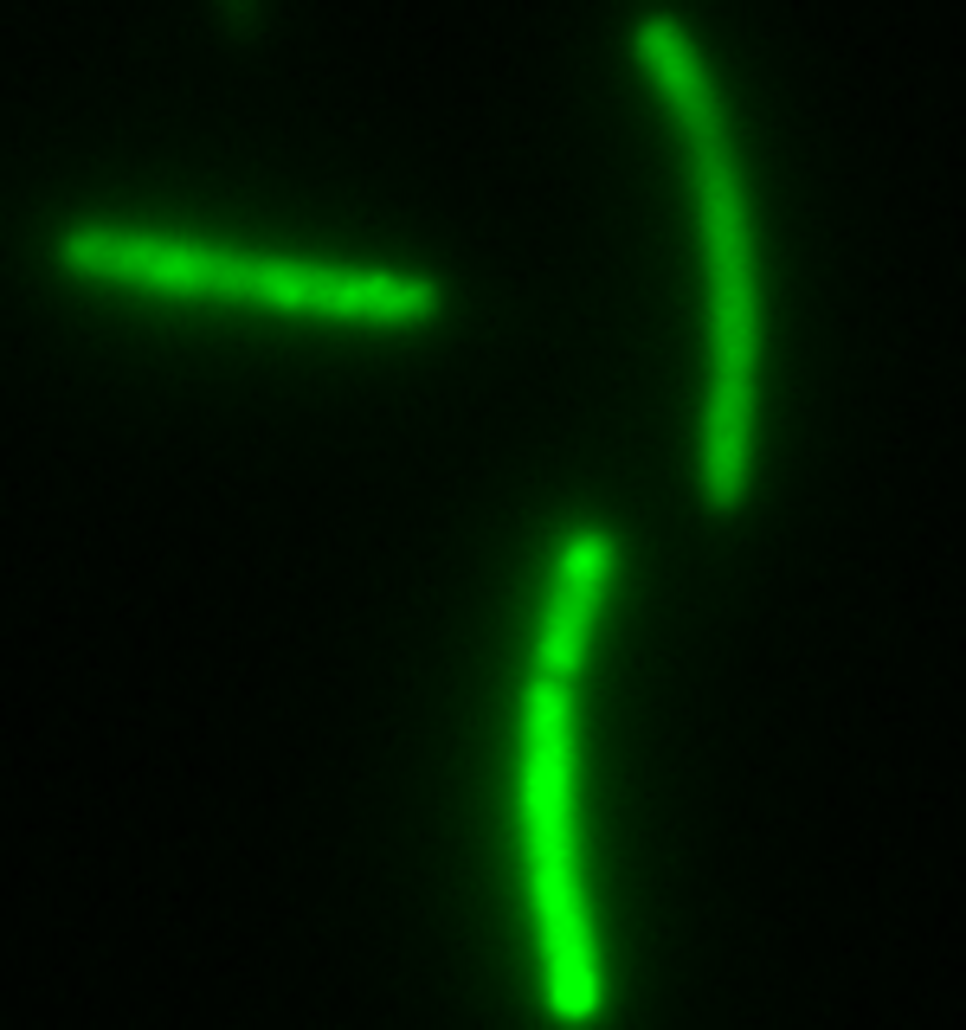

Supplement: Supplementary file 8 — Source Data Fig. 2 [file 44318_2023_26_MOESM8_ESM.zip › Figure 2/2A/bglG ts 42'C1.tif]

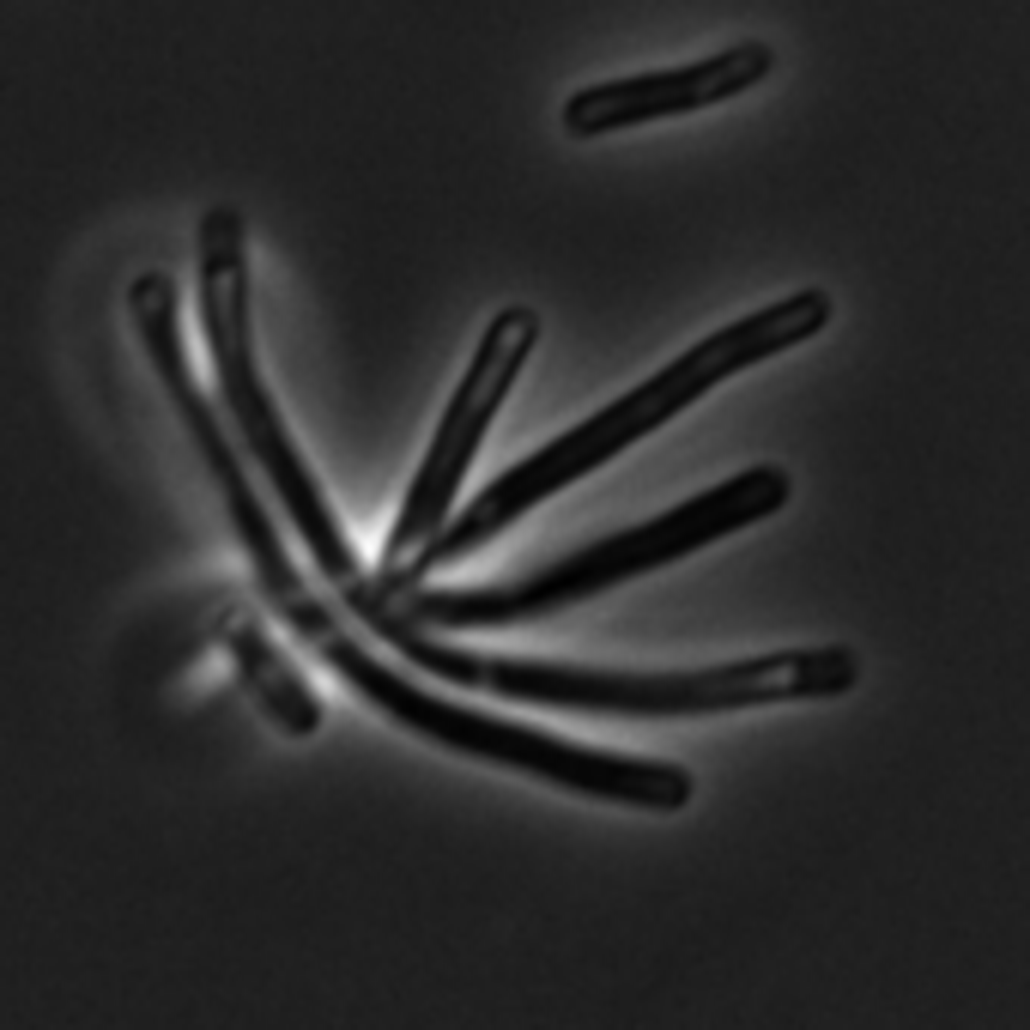

Supplement: Supplementary file 8 — Source Data Fig. 2 [file 44318_2023_26_MOESM8_ESM.zip › Figure 2/2A/cheA ts 42'C2.tif]

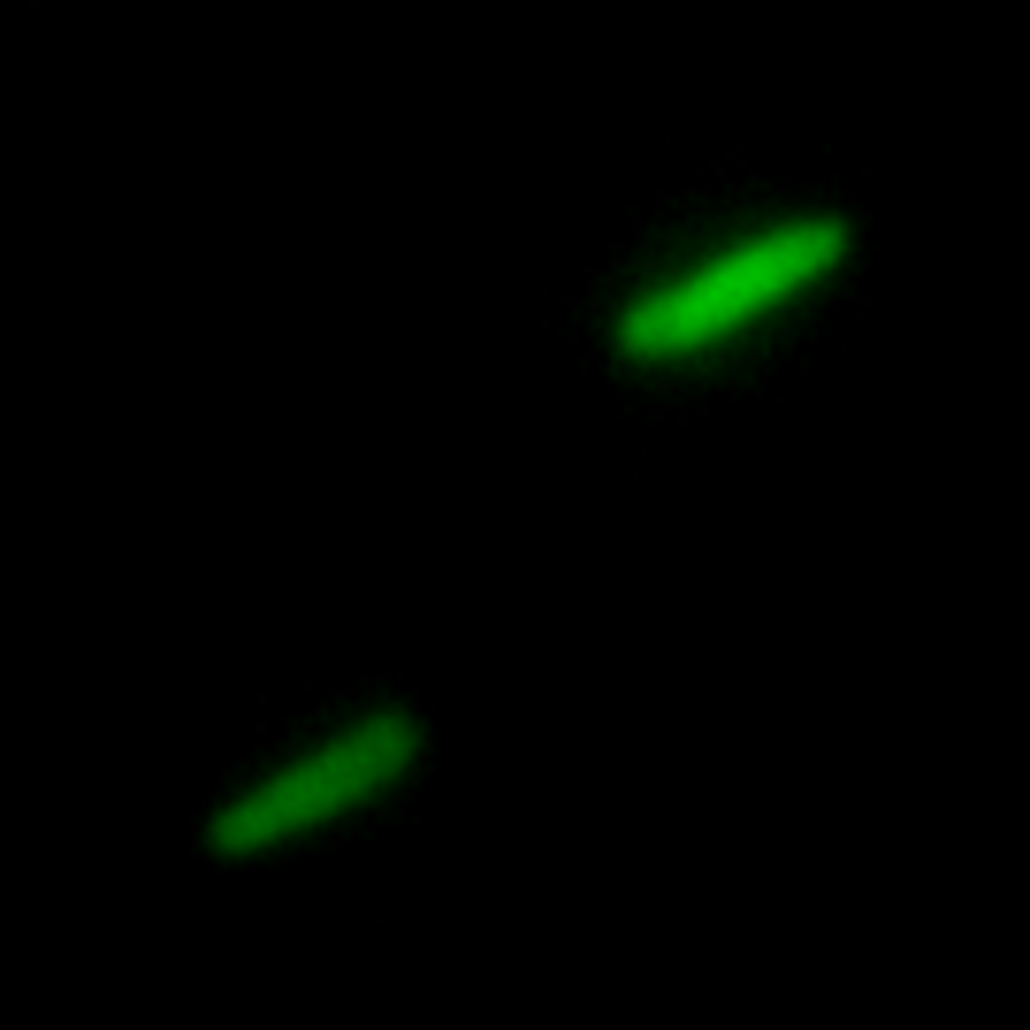

Supplement: Supplementary file 8 — Source Data Fig. 2 [file 44318_2023_26_MOESM8_ESM.zip › Figure 2/2C/dpnp cheA1.tif]

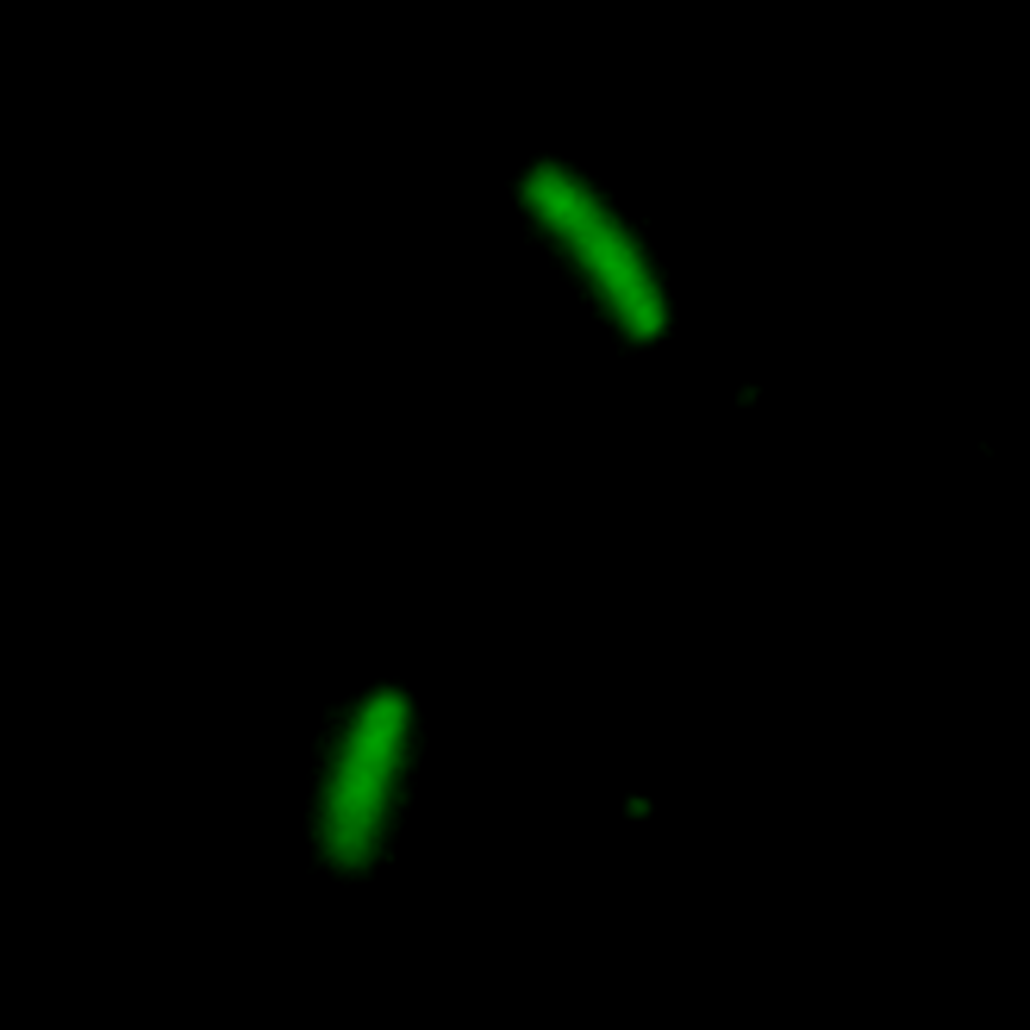

Supplement: Supplementary file 8 — Source Data Fig. 2 [file 44318_2023_26_MOESM8_ESM.zip › Figure 2/2C/drhlB cheA.tif]

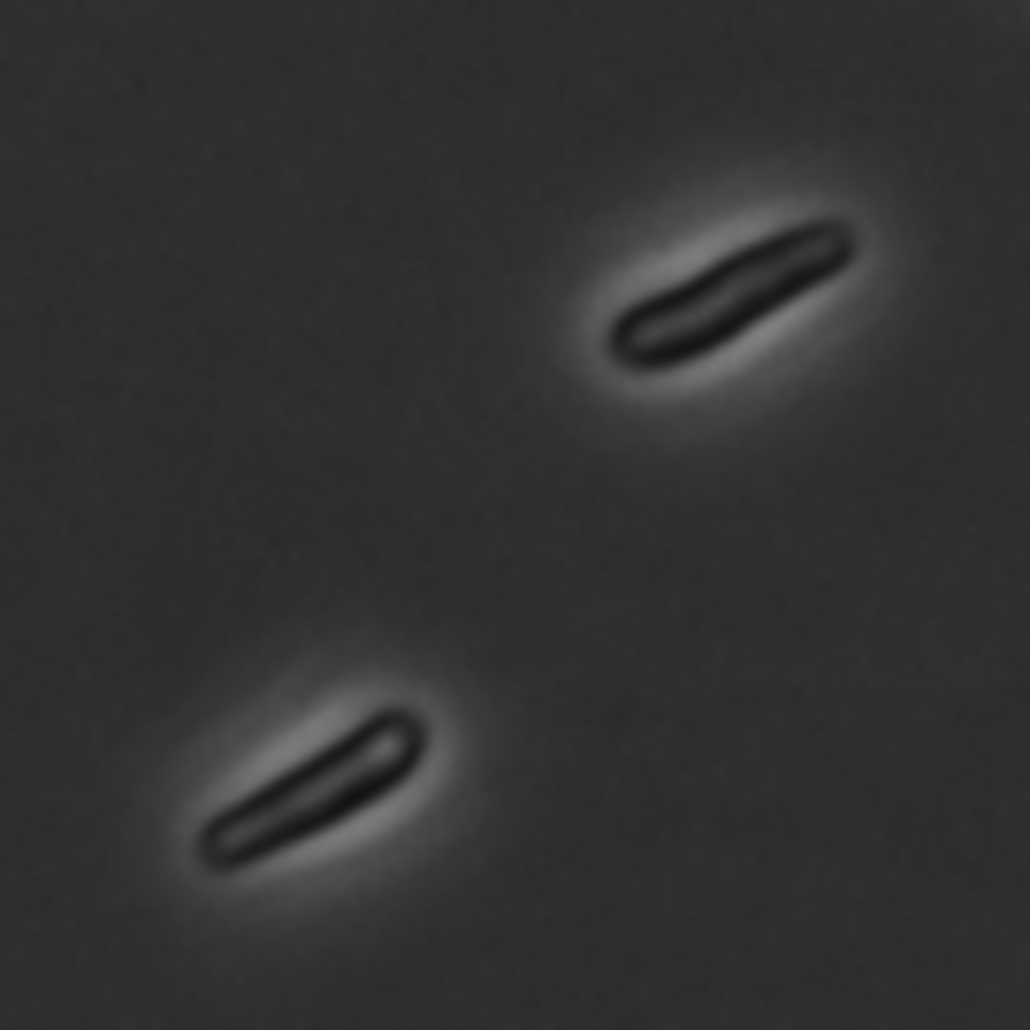

Supplement: Supplementary file 8 — Source Data Fig. 2 [file 44318_2023_26_MOESM8_ESM.zip › Figure 2/2C/dpnp cheA.tif]

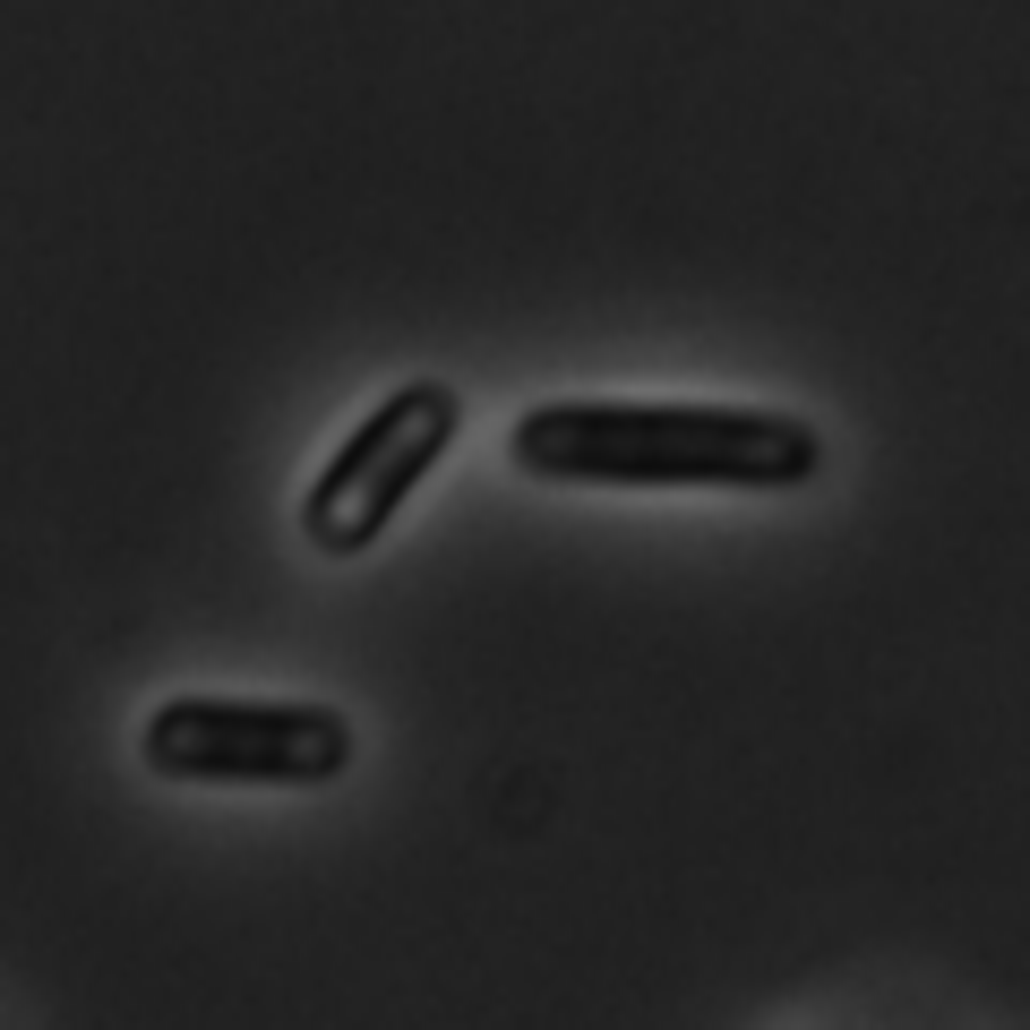

Supplement: Supplementary file 8 — Source Data Fig. 2 [file 44318_2023_26_MOESM8_ESM.zip › Figure 2/2C/dpnp bglG1.tif]

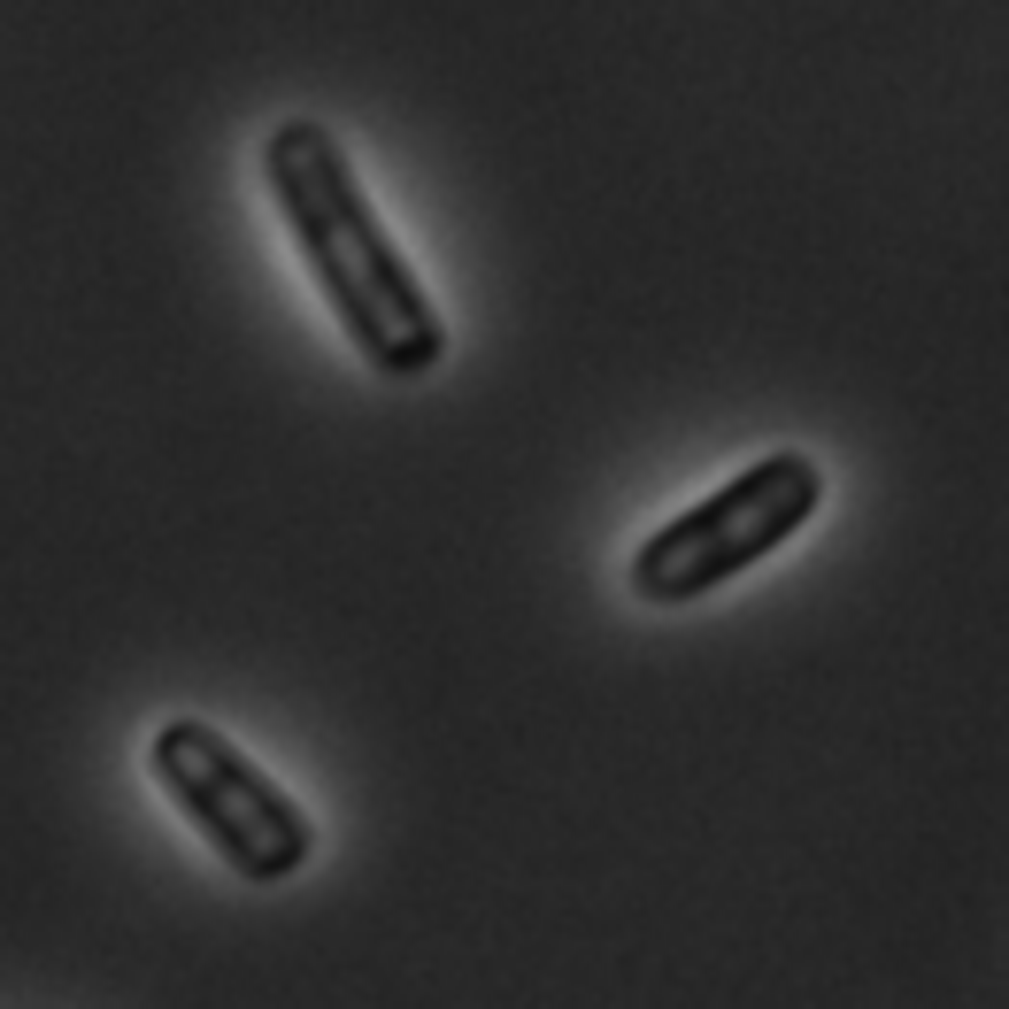

Supplement: Supplementary file 8 — Source Data Fig. 2 [file 44318_2023_26_MOESM8_ESM.zip › Figure 2/2C/drhlB bglG1.tif]

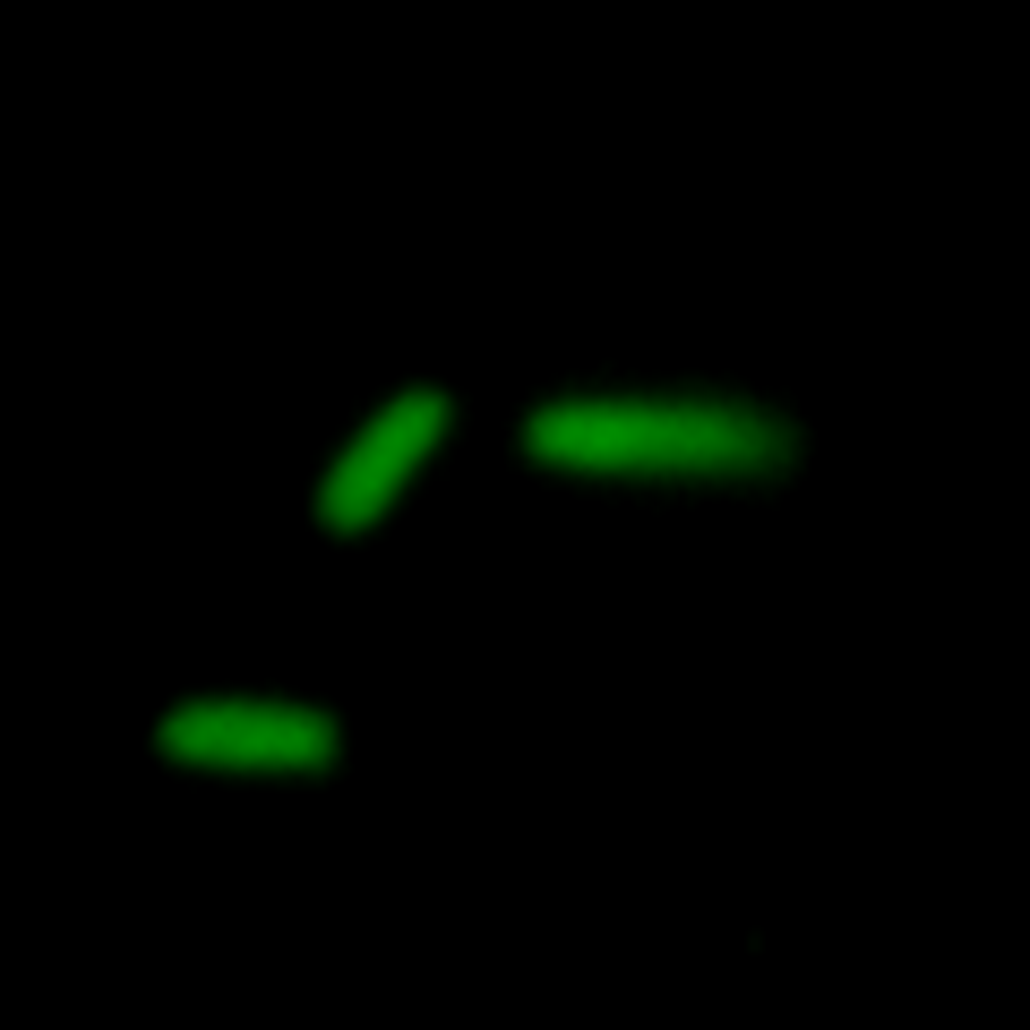

Supplement: Supplementary file 8 — Source Data Fig. 2 [file 44318_2023_26_MOESM8_ESM.zip › Figure 2/2C/dpnp bglG.tif]

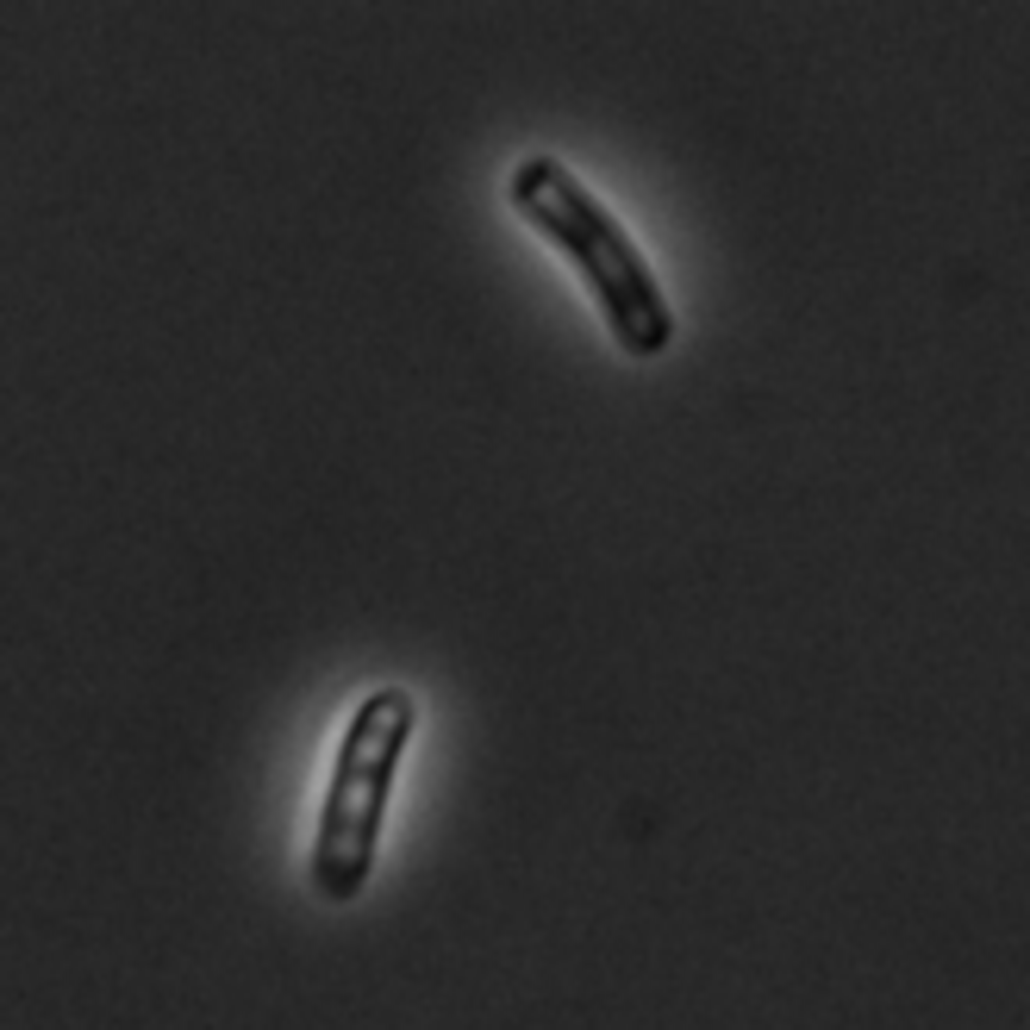

Supplement: Supplementary file 8 — Source Data Fig. 2 [file 44318_2023_26_MOESM8_ESM.zip › Figure 2/2C/drhlB cheA1.tif]

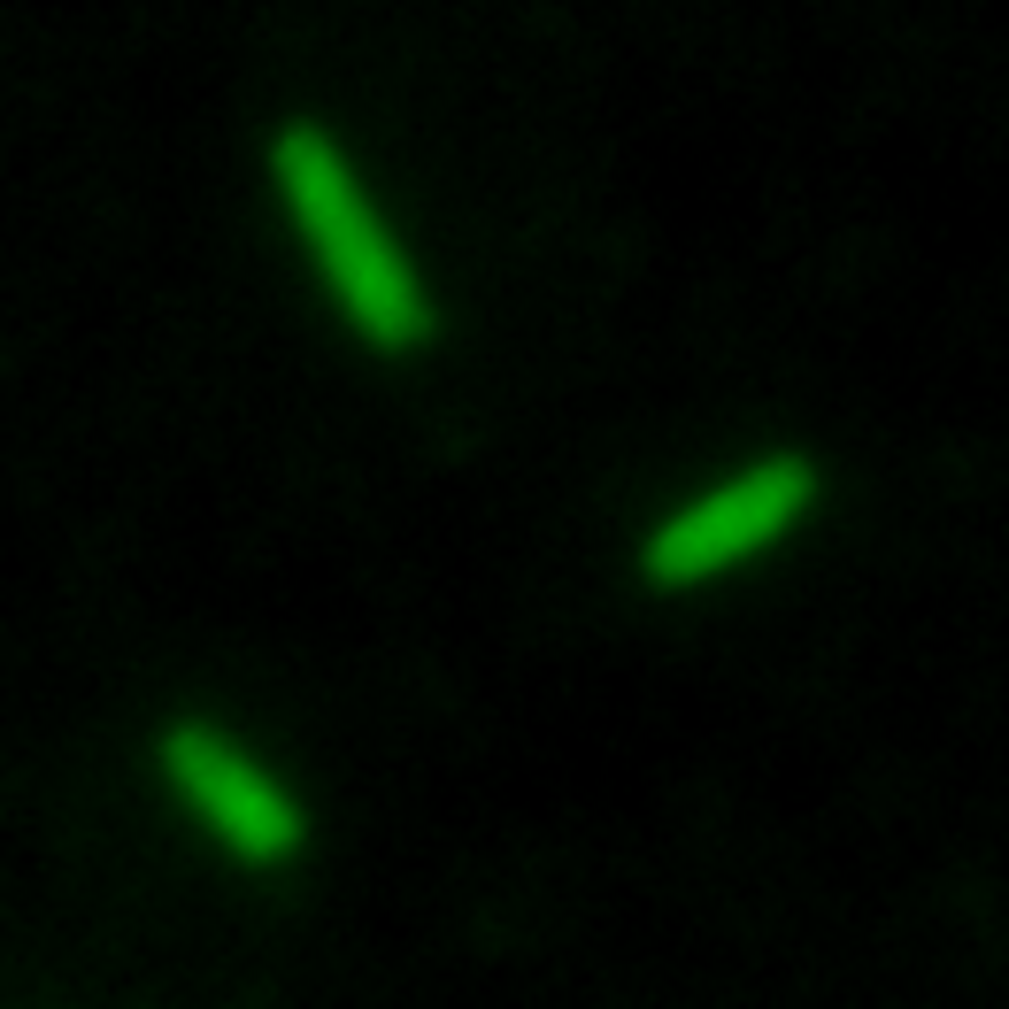

Supplement: Supplementary file 8 — Source Data Fig. 2 [file 44318_2023_26_MOESM8_ESM.zip › Figure 2/2C/drhlB bglG.tif]

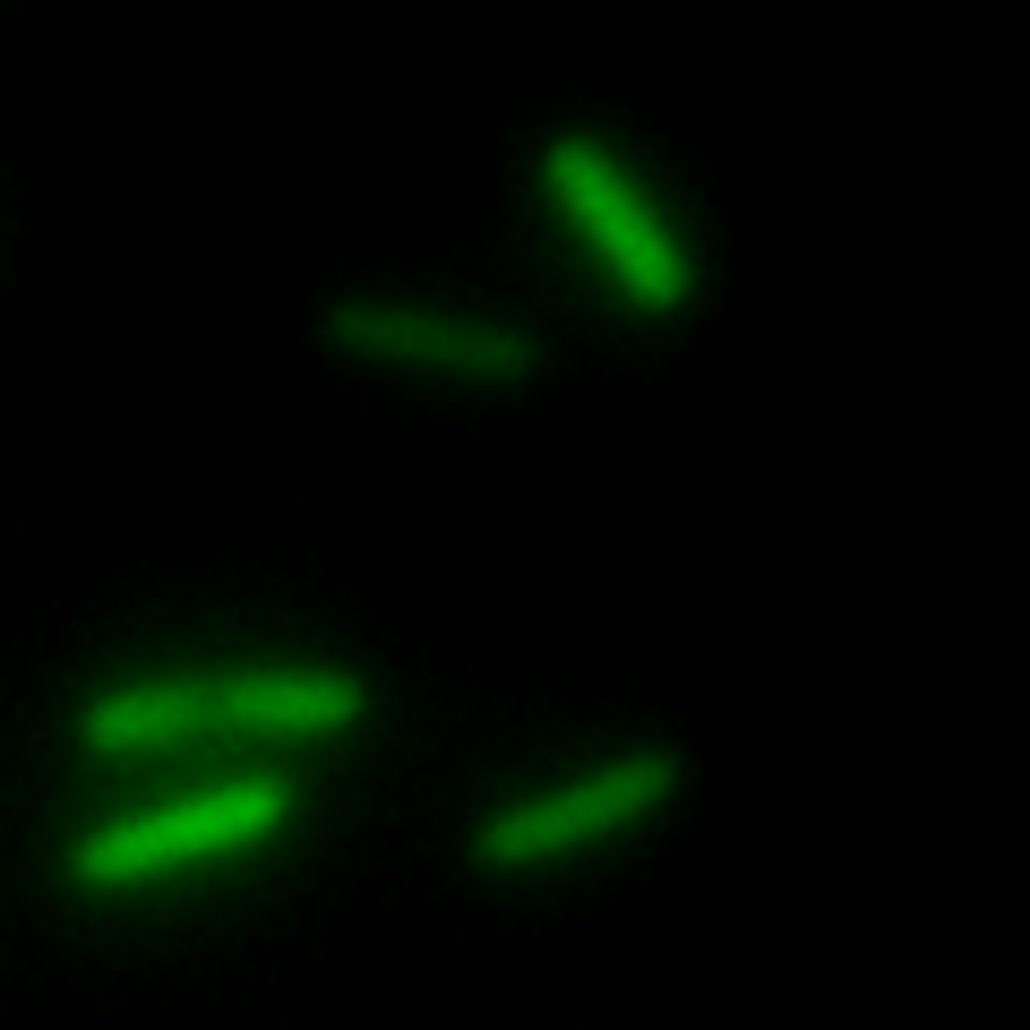

Supplement: Supplementary file 8 — Source Data Fig. 2 [file 44318_2023_26_MOESM8_ESM.zip › Figure 2/2B/eno-2 cheA.tif]

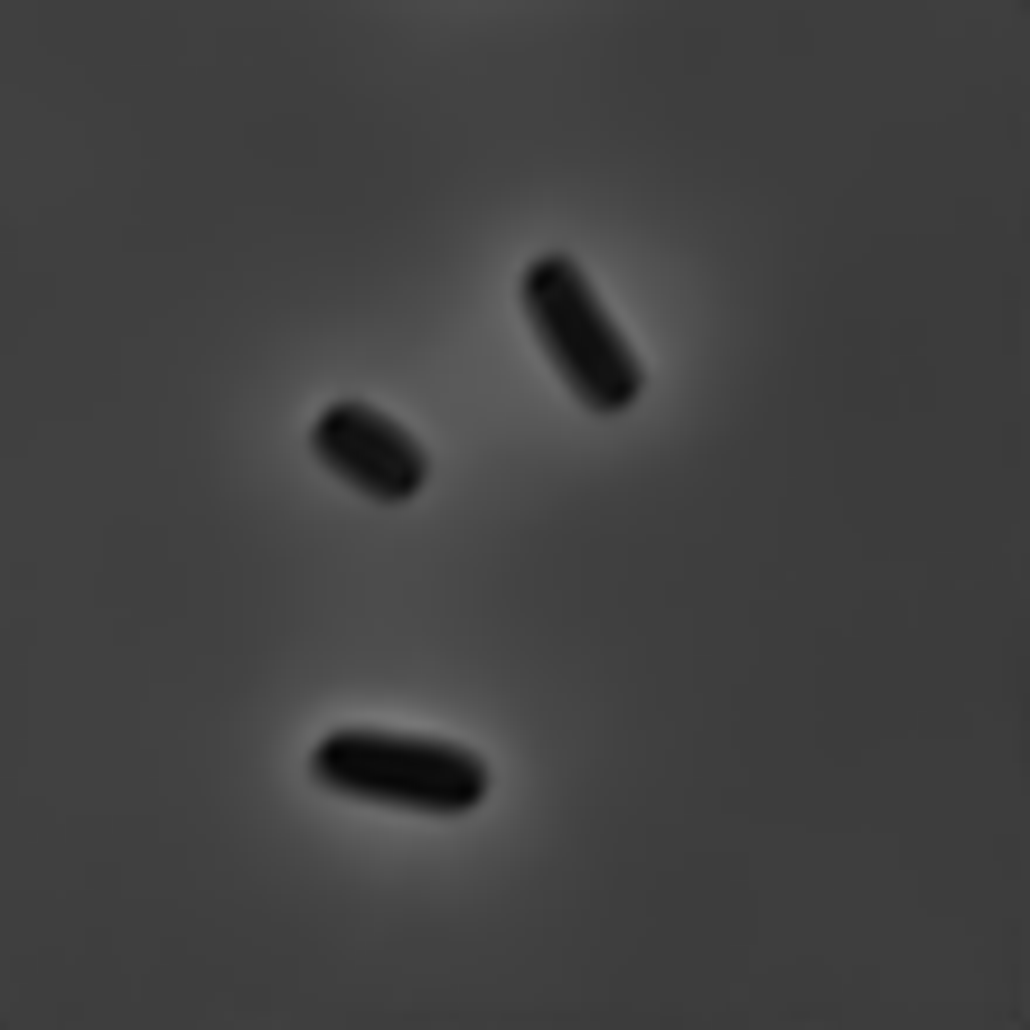

Supplement: Supplementary file 8 — Source Data Fig. 2 [file 44318_2023_26_MOESM8_ESM.zip › Figure 2/2B/eno-2 bglG1 wt.tif]

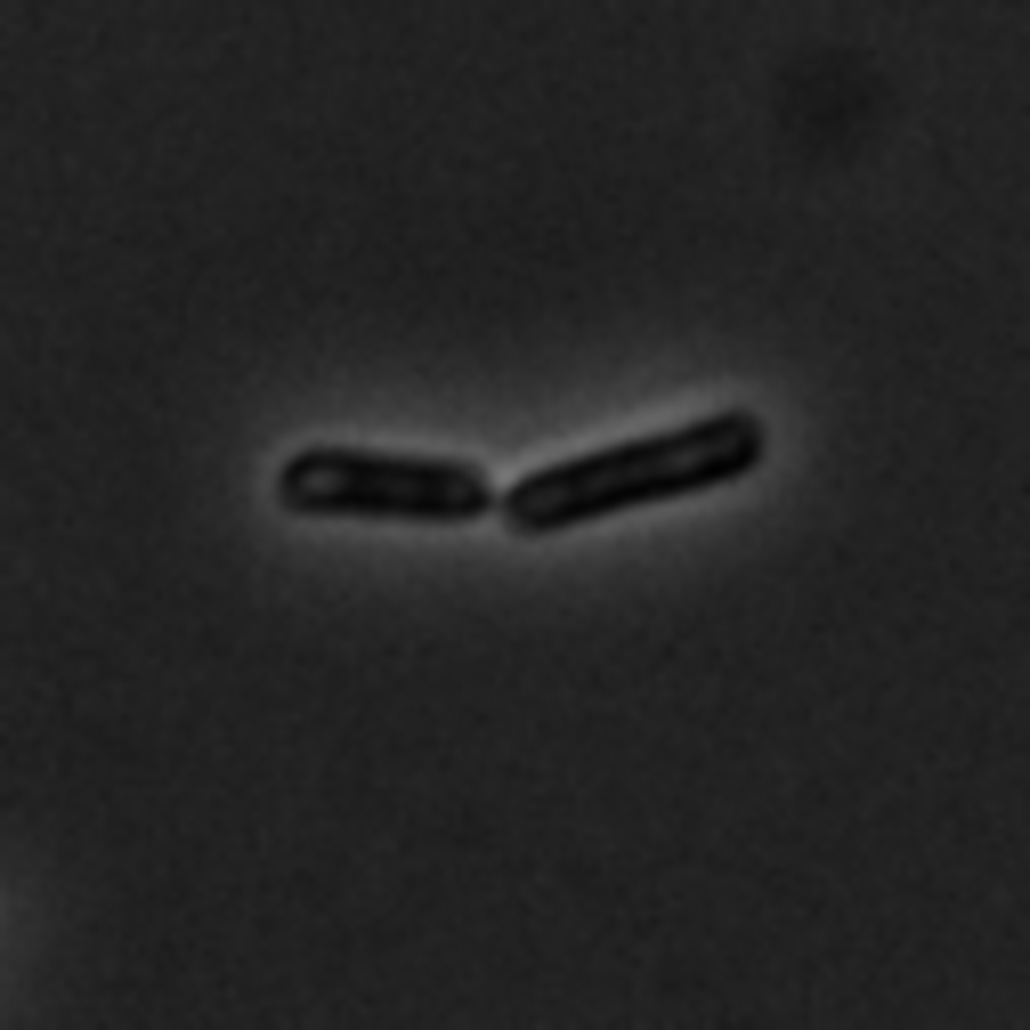

Supplement: Supplementary file 8 — Source Data Fig. 2 [file 44318_2023_26_MOESM8_ESM.zip › Figure 2/2B/eno-2 bglG1.tif]

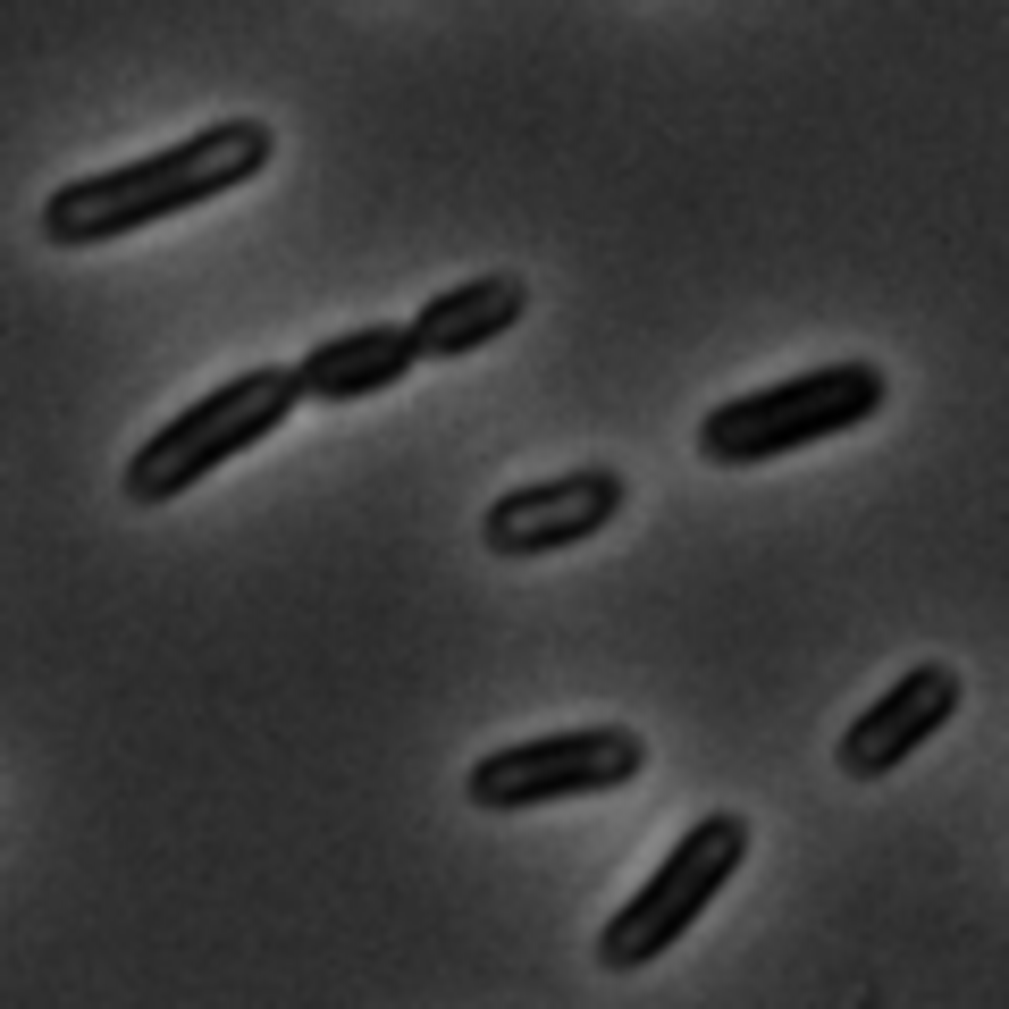

Supplement: Supplementary file 8 — Source Data Fig. 2 [file 44318_2023_26_MOESM8_ESM.zip › Figure 2/2B/eno-2 chheA1 wt.tif]

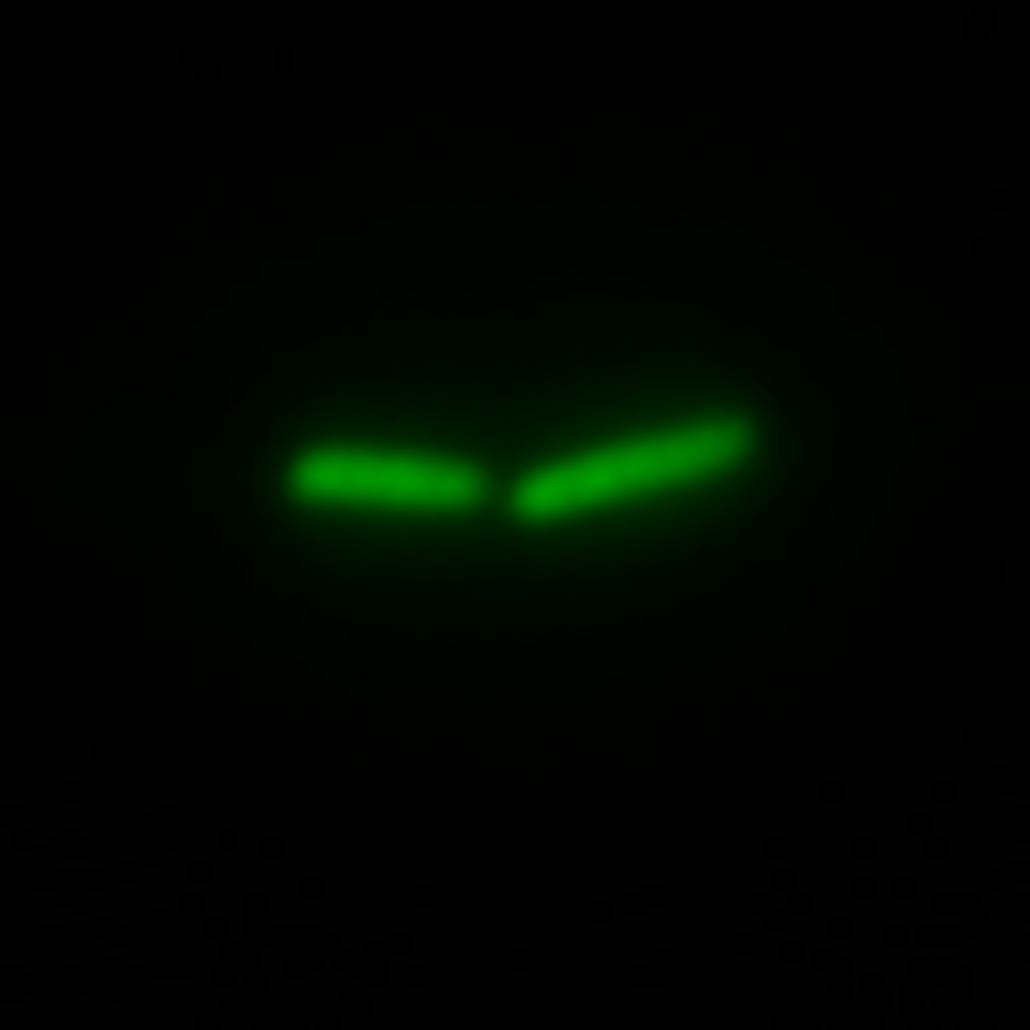

Supplement: Supplementary file 8 — Source Data Fig. 2 [file 44318_2023_26_MOESM8_ESM.zip › Figure 2/2B/eno-2 bglG.tif]

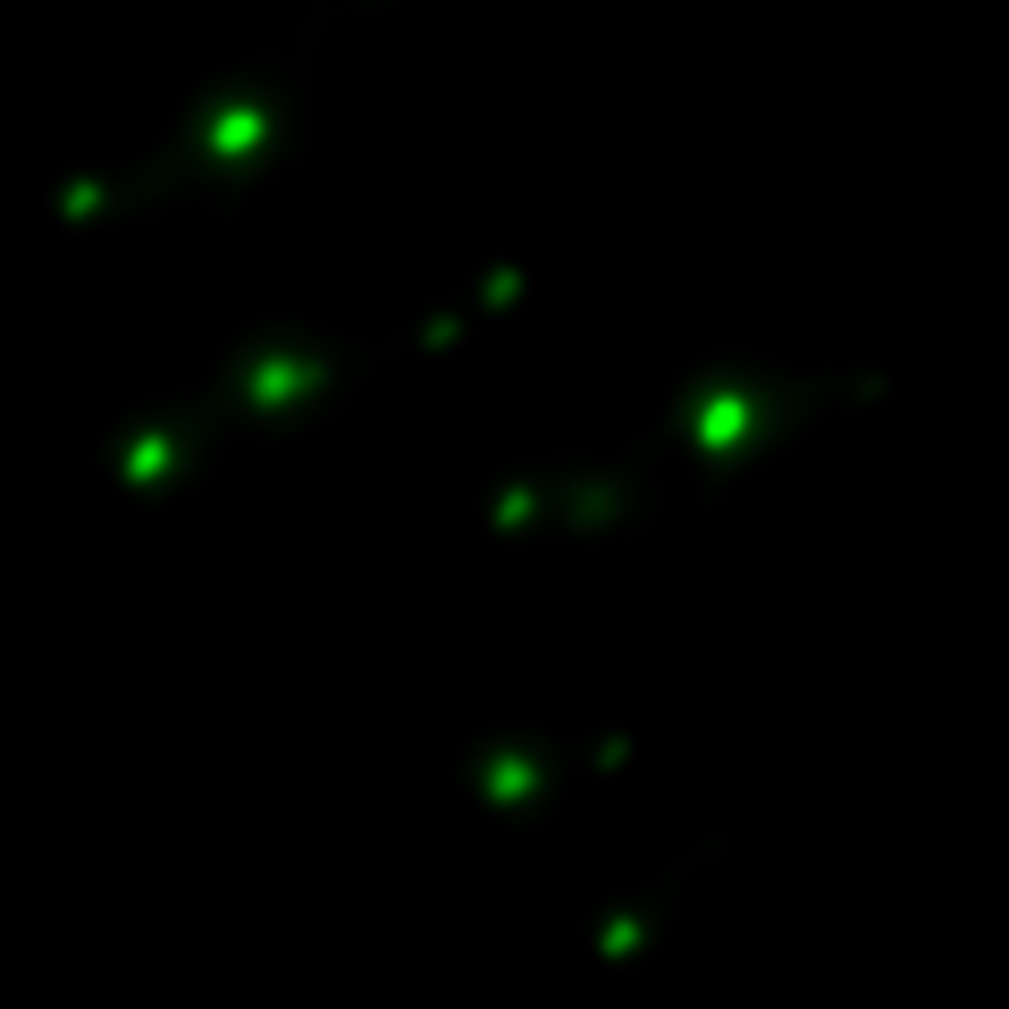

Supplement: Supplementary file 8 — Source Data Fig. 2 [file 44318_2023_26_MOESM8_ESM.zip › Figure 2/2B/eno-2 chheA wt.tif]

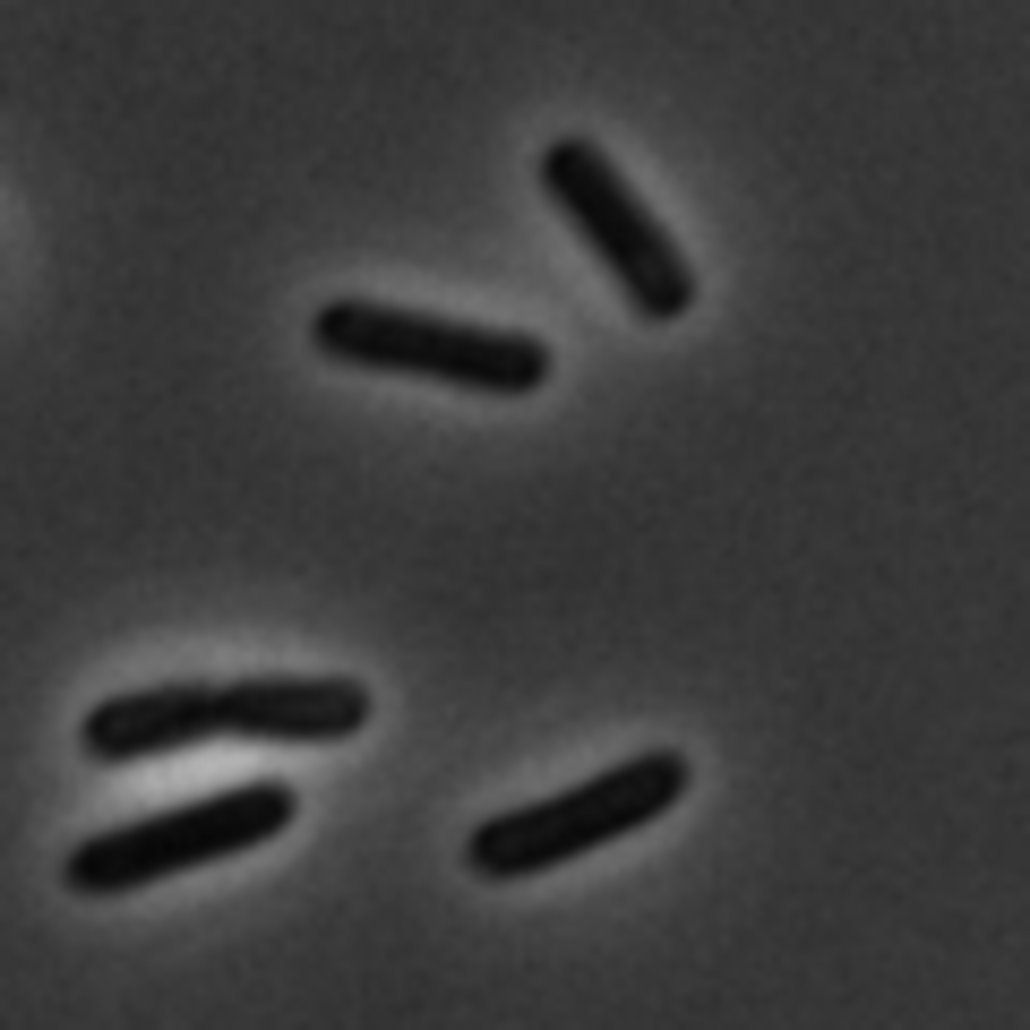

Supplement: Supplementary file 8 — Source Data Fig. 2 [file 44318_2023_26_MOESM8_ESM.zip › Figure 2/2B/eno-2 cheA1.tif]

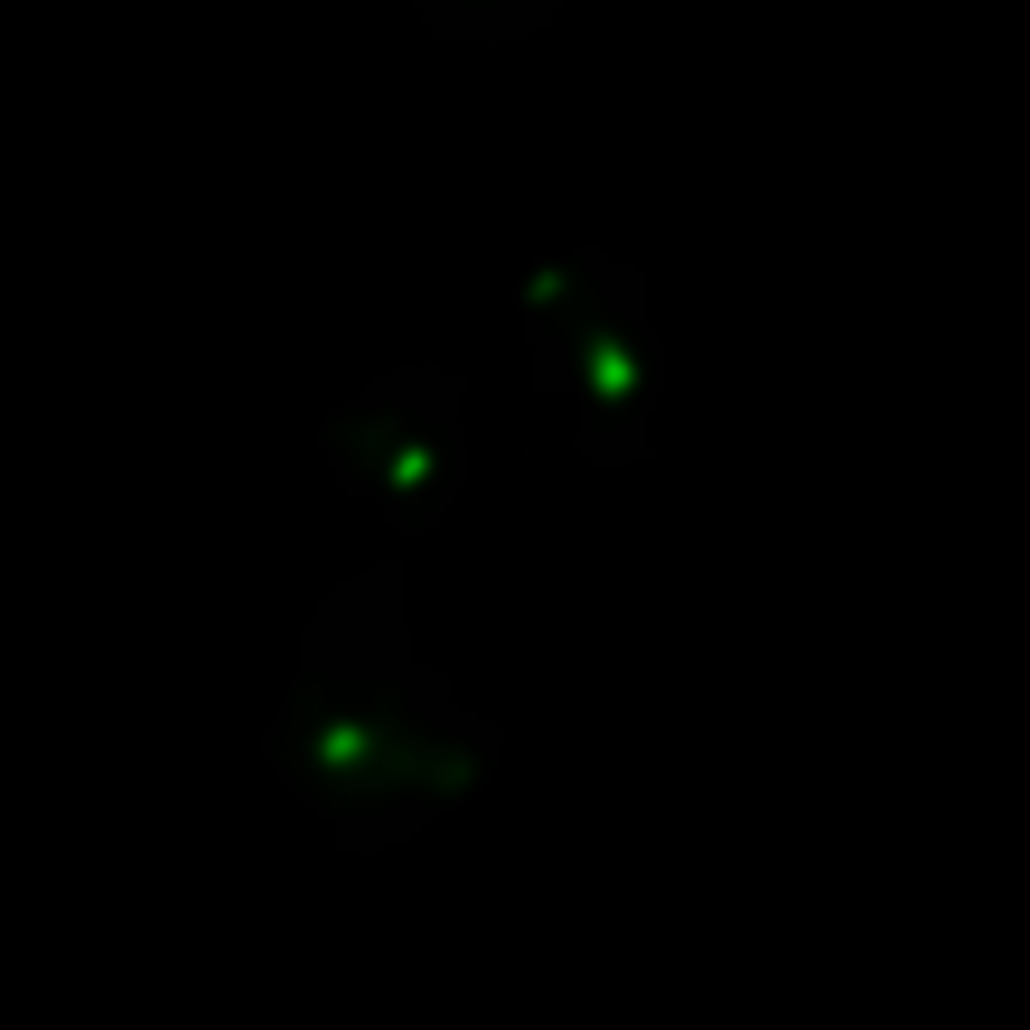

Supplement: Supplementary file 8 — Source Data Fig. 2 [file 44318_2023_26_MOESM8_ESM.zip › Figure 2/2B/eno-2 bglG wt.tif]

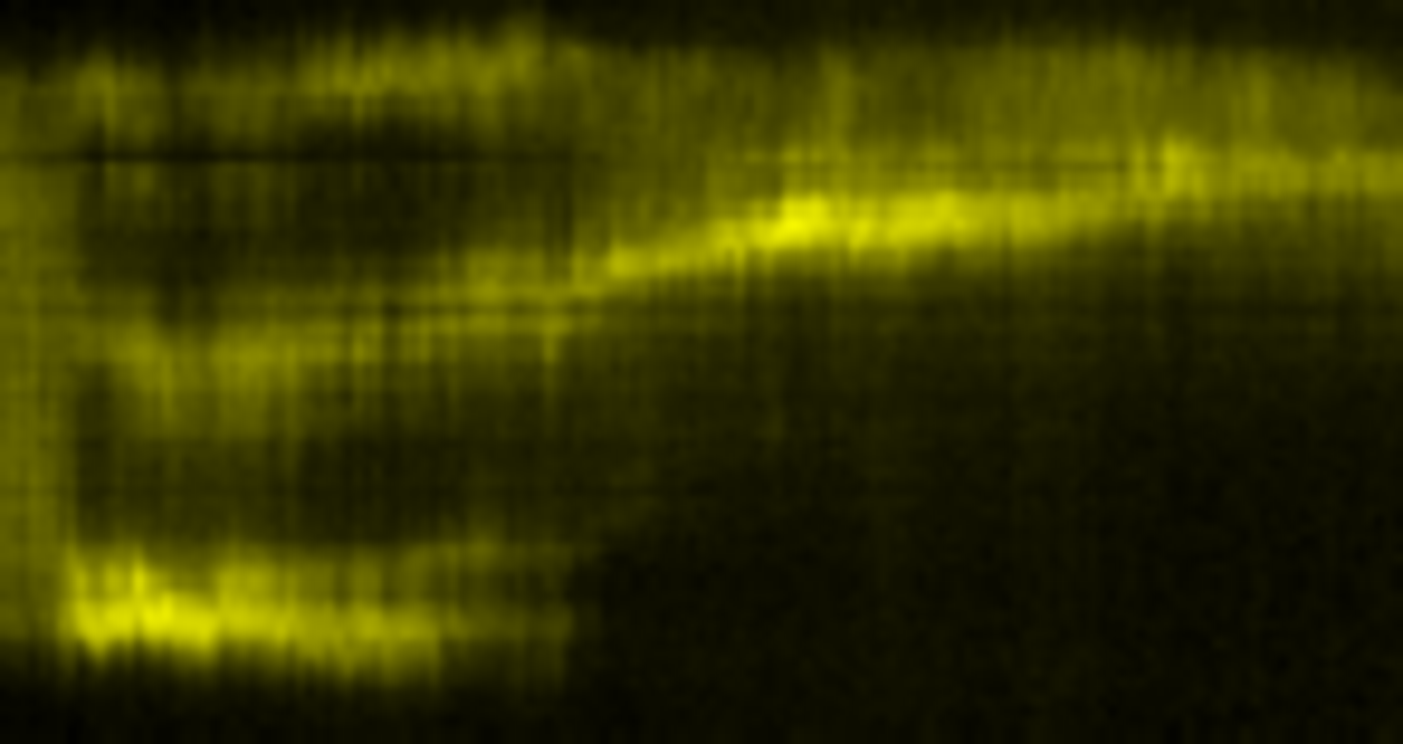

Supplement: Supplementary file 9 — Source Data Fig. 4 [file 44318_2023_26_MOESM9_ESM.zip › Figure 4/4B/Kymo.tif]

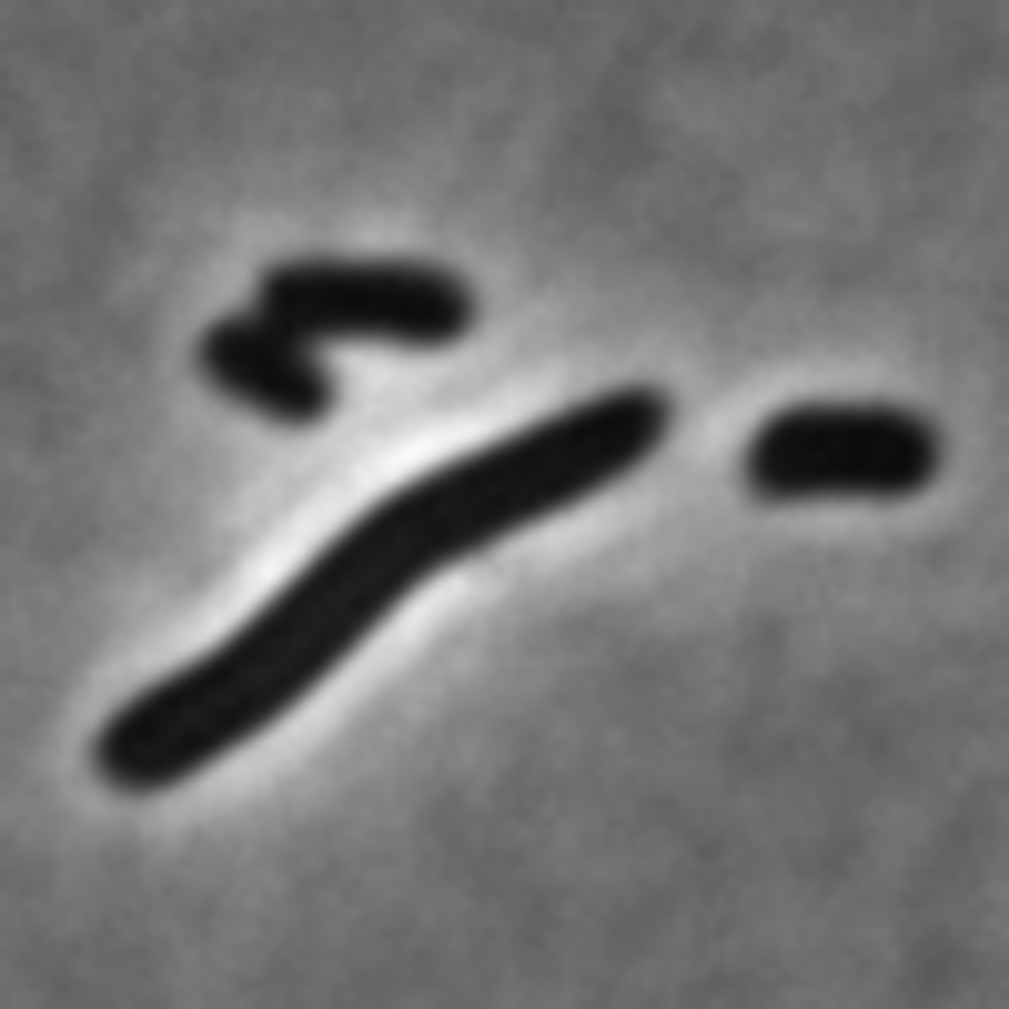

Supplement: Supplementary file 9 — Source Data Fig. 4 [file 44318_2023_26_MOESM9_ESM.zip › Figure 4/4A/rne-yfp dMin1.tif]

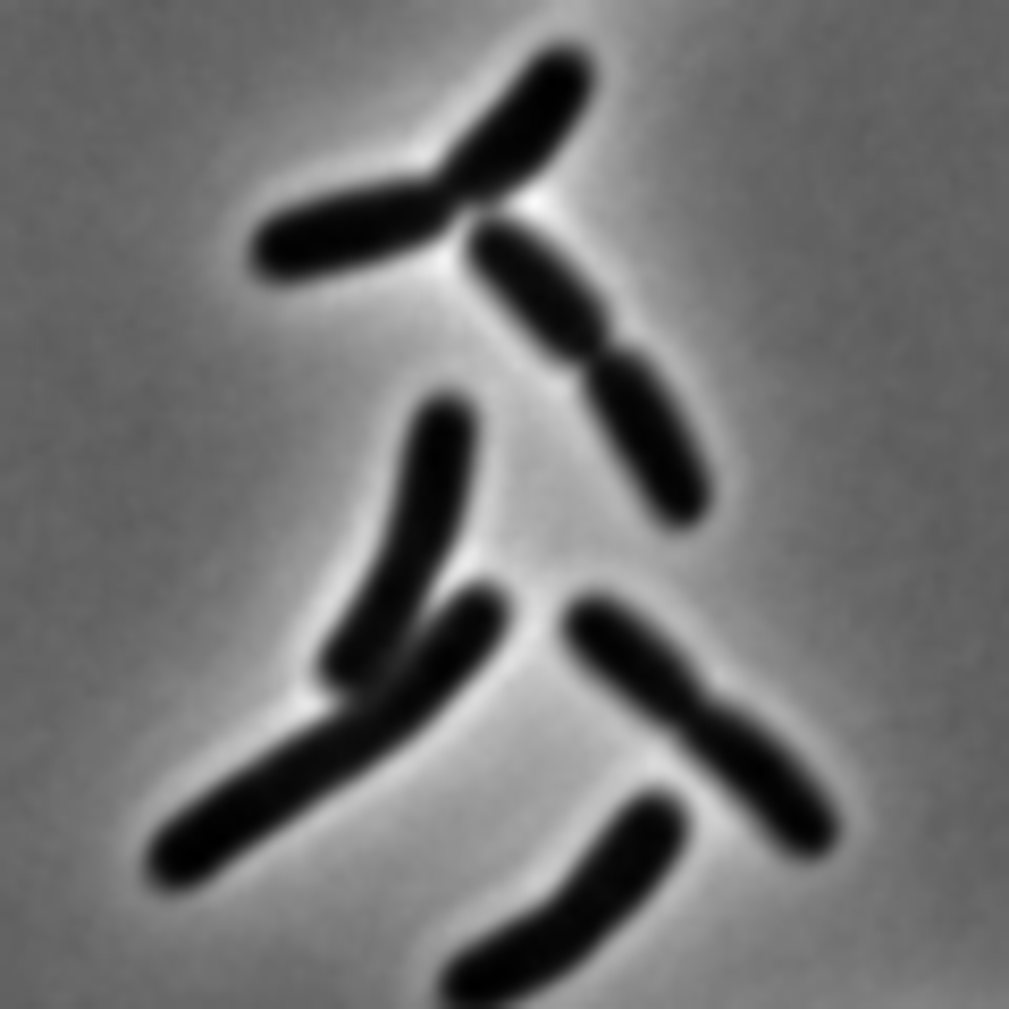

Supplement: Supplementary file 9 — Source Data Fig. 4 [file 44318_2023_26_MOESM9_ESM.zip › Figure 4/4A/rne-yfp WT1.tif]

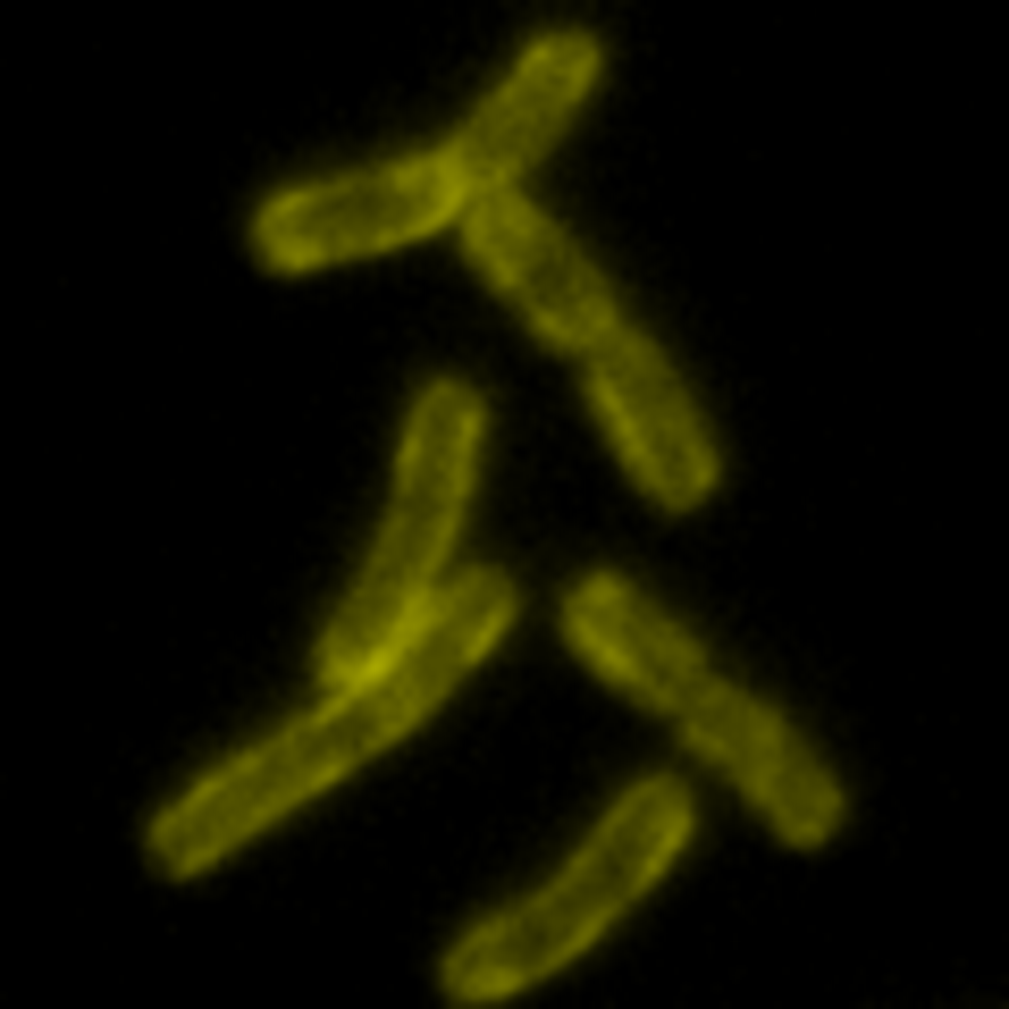

Supplement: Supplementary file 9 — Source Data Fig. 4 [file 44318_2023_26_MOESM9_ESM.zip › Figure 4/4A/rne-yfp WT.tif]

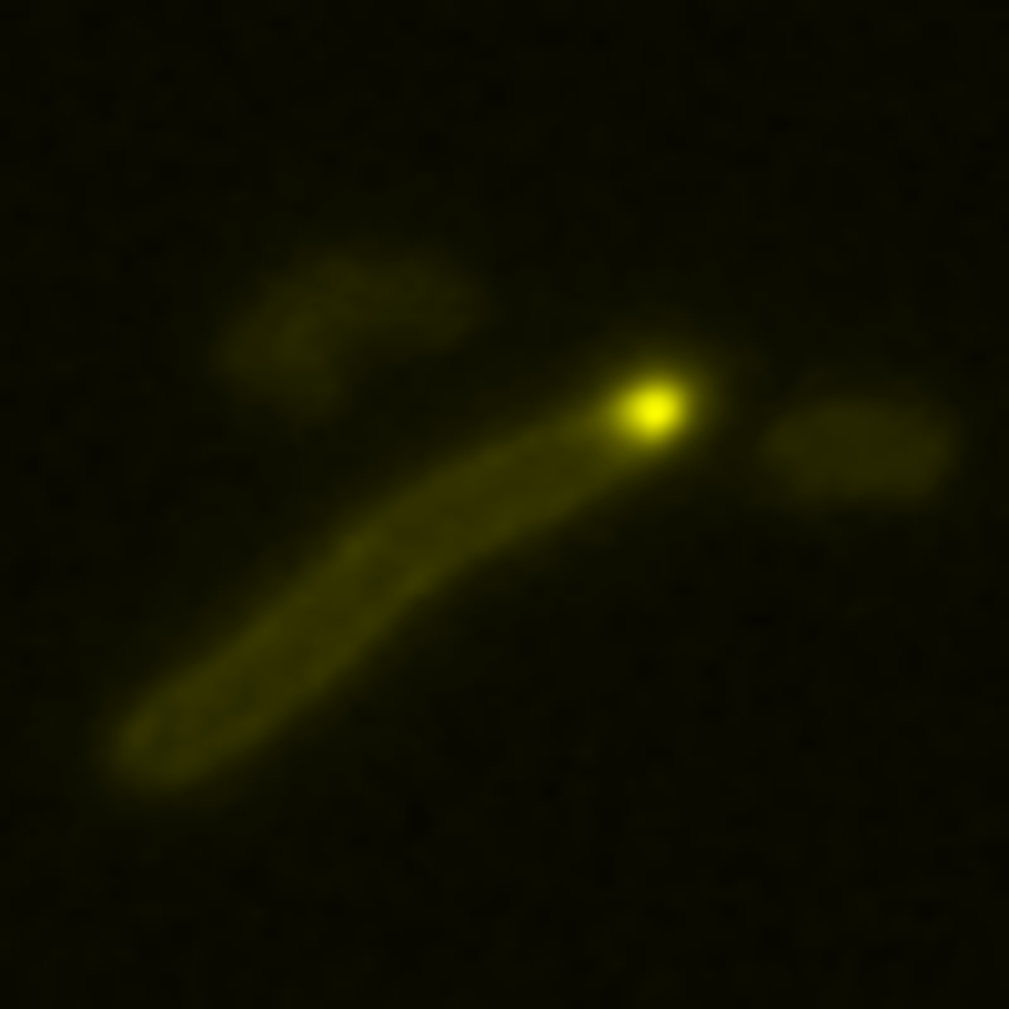

Supplement: Supplementary file 9 — Source Data Fig. 4 [file 44318_2023_26_MOESM9_ESM.zip › Figure 4/4A/rne-yfp dMin.tif]

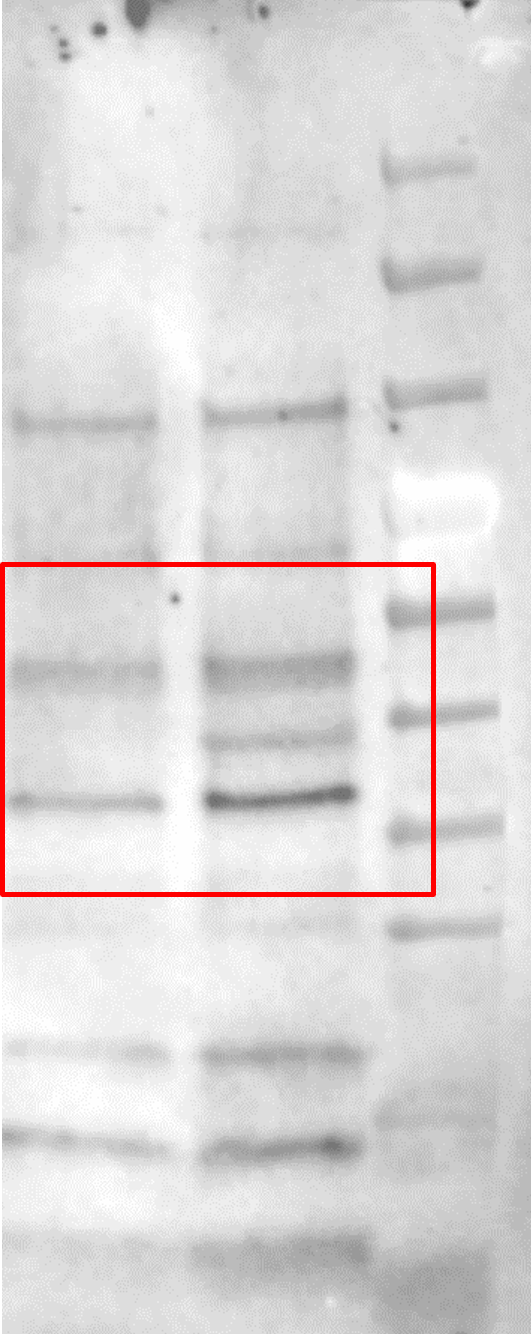

Supplement: Supplementary file 10 — Source Data Fig. 5 [file 44318_2023_26_MOESM10_ESM.zip › Figure 5/5C/5C.tif]

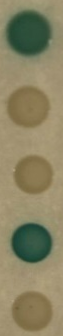

Supplement: Supplementary file 10 — Source Data Fig. 5 [file 44318_2023_26_MOESM10_ESM.zip › Figure 5/5D/5D.tif]

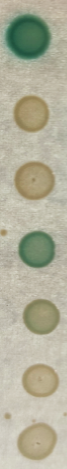

Supplement: Supplementary file 10 — Source Data Fig. 5 [file 44318_2023_26_MOESM10_ESM.zip › Figure 5/5E/5E.png]

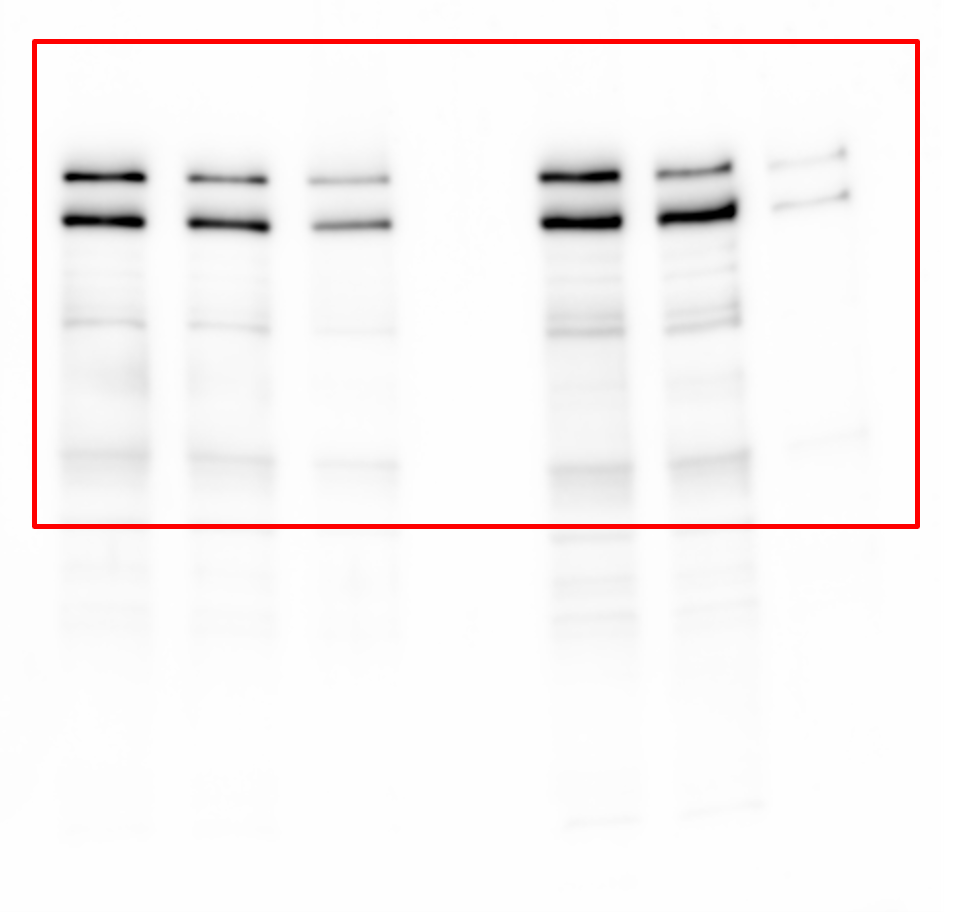

Supplement: Supplementary file 11 — Source Data Fig. 6 [file 44318_2023_26_MOESM11_ESM.zip › Figure 6/6B/6B.tif]

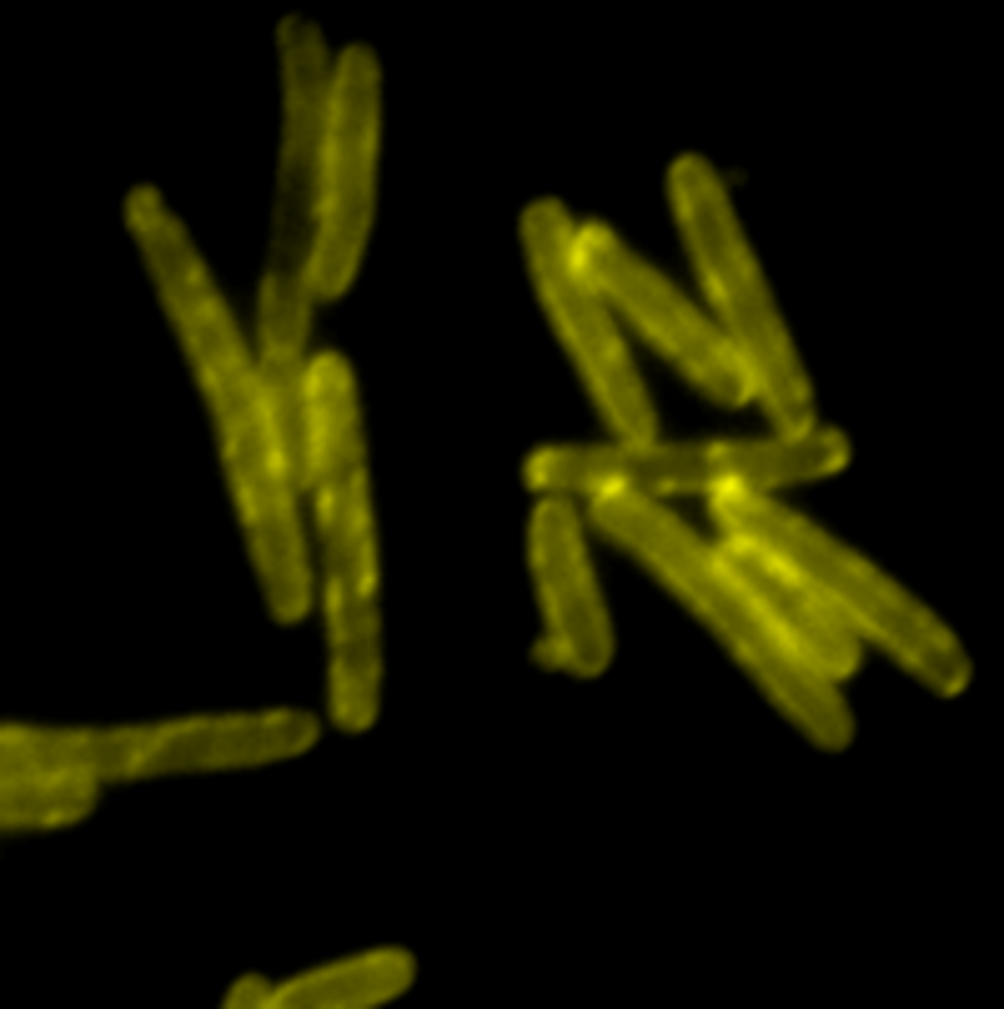

Supplement: Supplementary file 11 — Source Data Fig. 6 [file 44318_2023_26_MOESM11_ESM.zip › Figure 6/6D/dMin rif.tif]

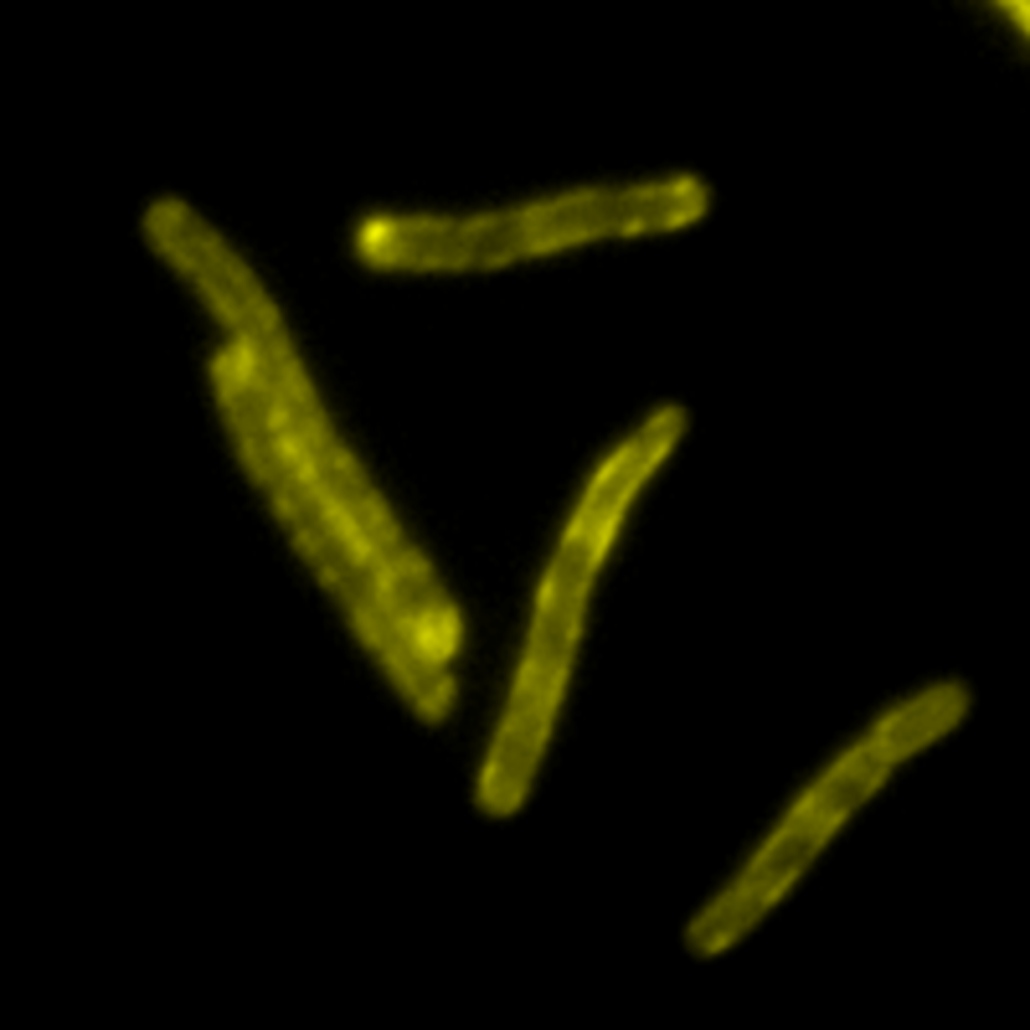

Supplement: Supplementary file 11 — Source Data Fig. 6 [file 44318_2023_26_MOESM11_ESM.zip › Figure 6/6D/dMin untreated.tif]

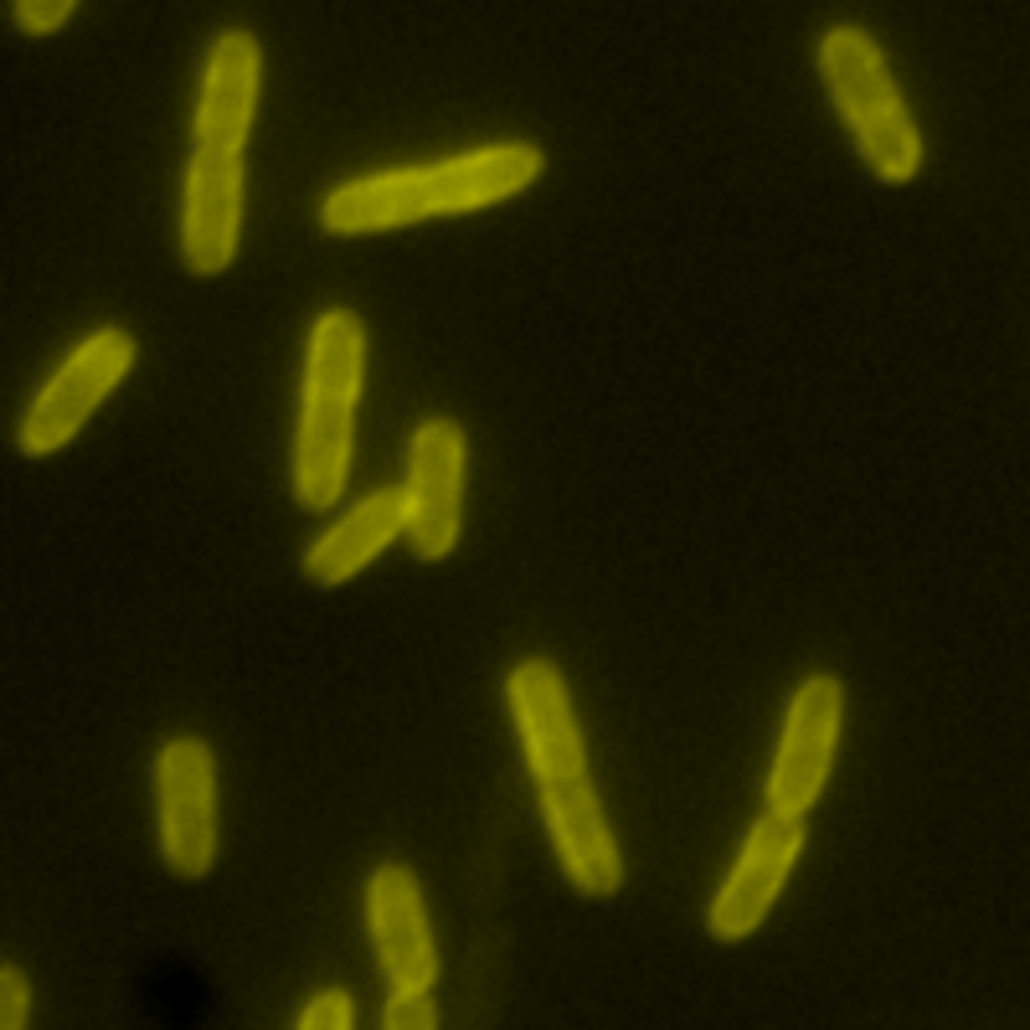

Supplement: Supplementary file 11 — Source Data Fig. 6 [file 44318_2023_26_MOESM11_ESM.zip › Figure 6/6D/WT rif.tif]

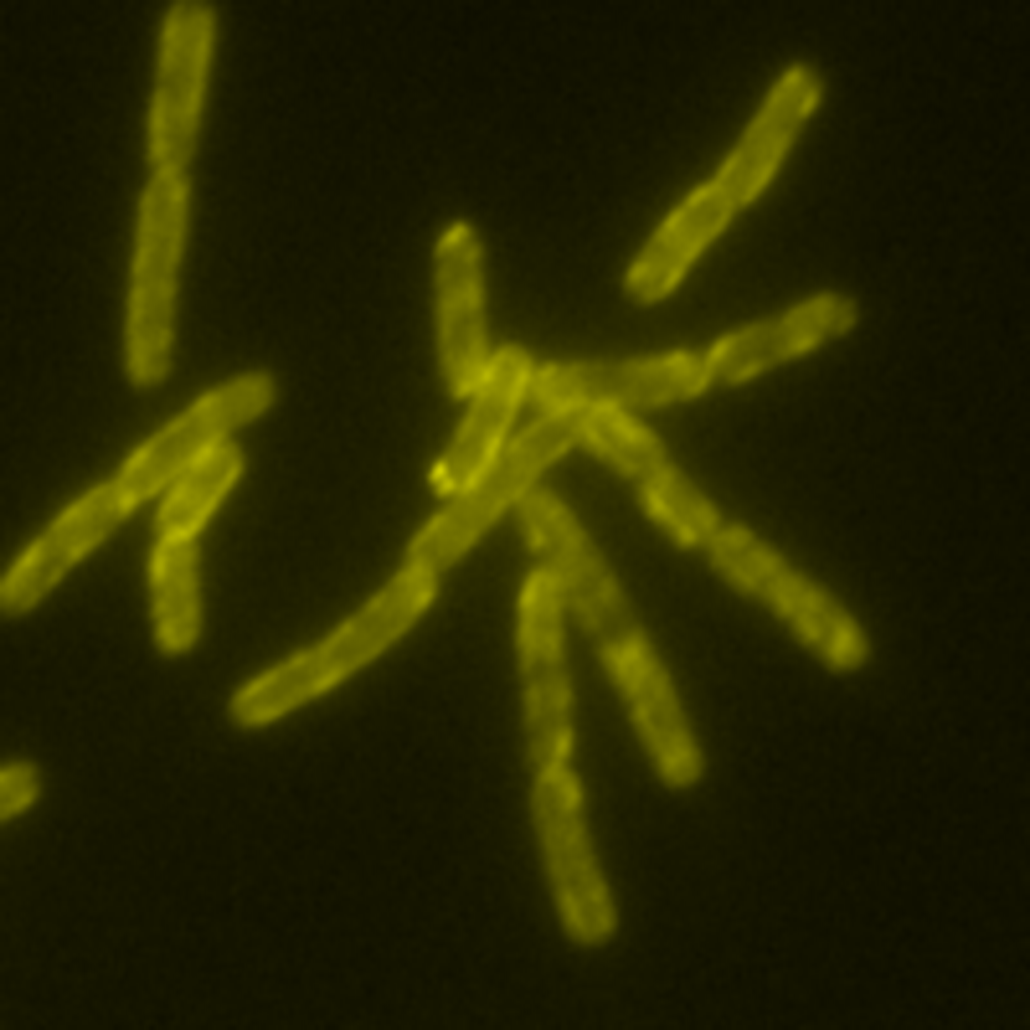

Supplement: Supplementary file 11 — Source Data Fig. 6 [file 44318_2023_26_MOESM11_ESM.zip › Figure 6/6D/WT untreated.tif]

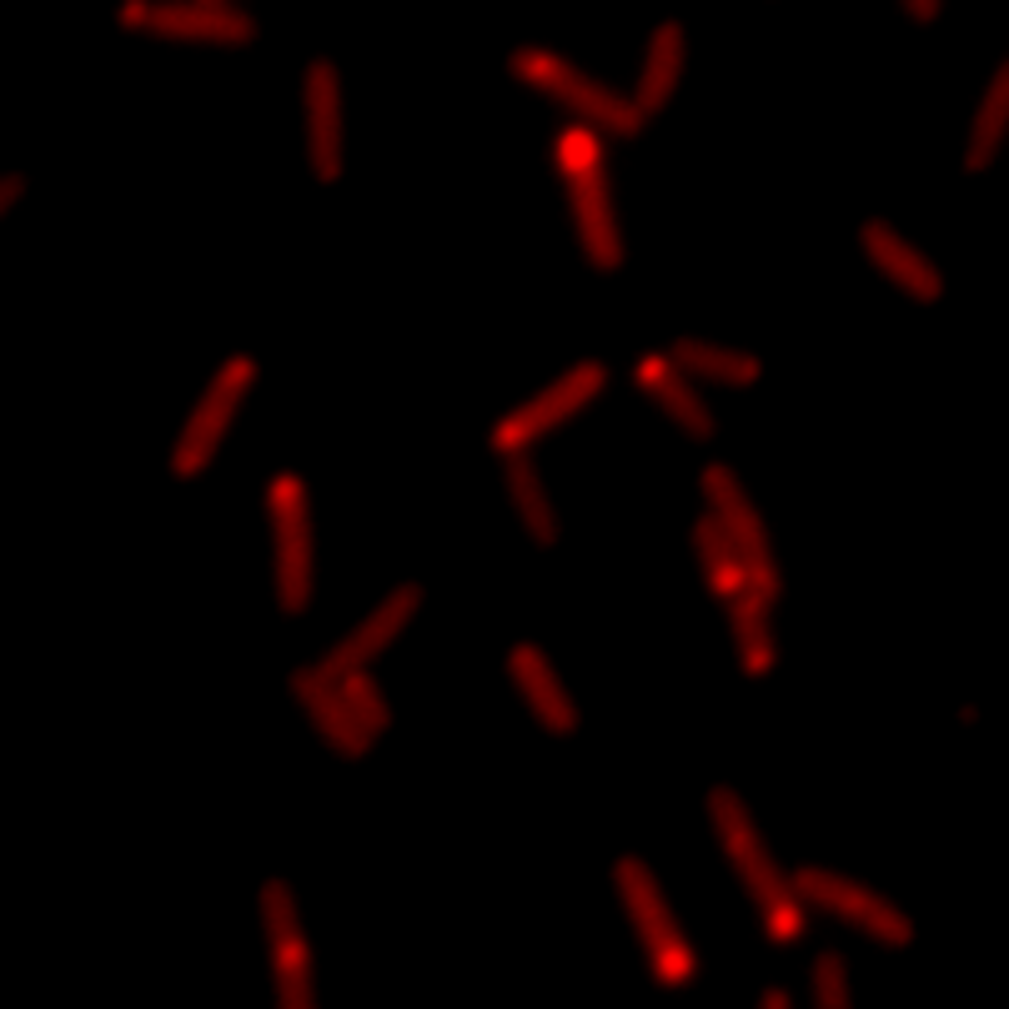

Supplement: Supplementary file 12 — Source Data Fig. 7 [file 44318_2023_26_MOESM12_ESM.zip › Figure 7/7D/E1-mCh.tif]

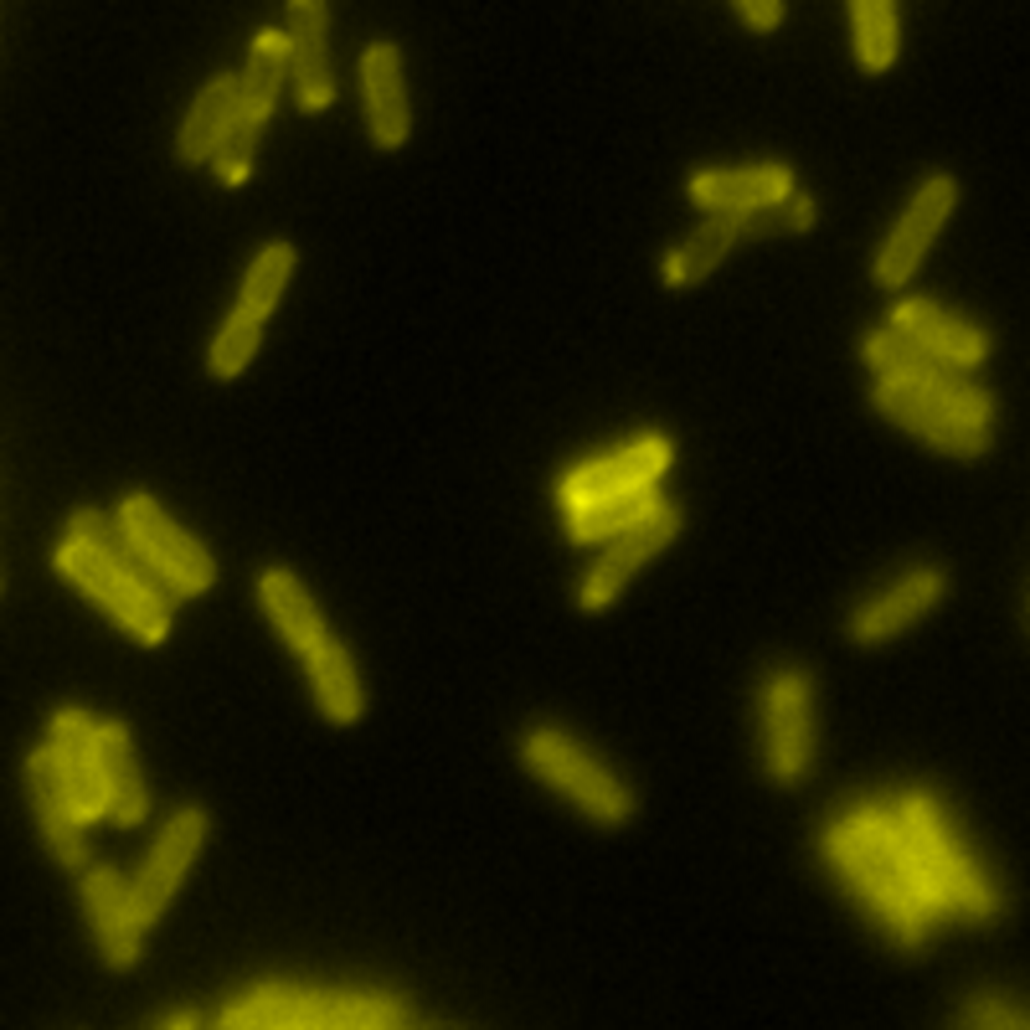

Supplement: Supplementary file 12 — Source Data Fig. 7 [file 44318_2023_26_MOESM12_ESM.zip › Figure 7/7C/tar-yfp WT.tif]

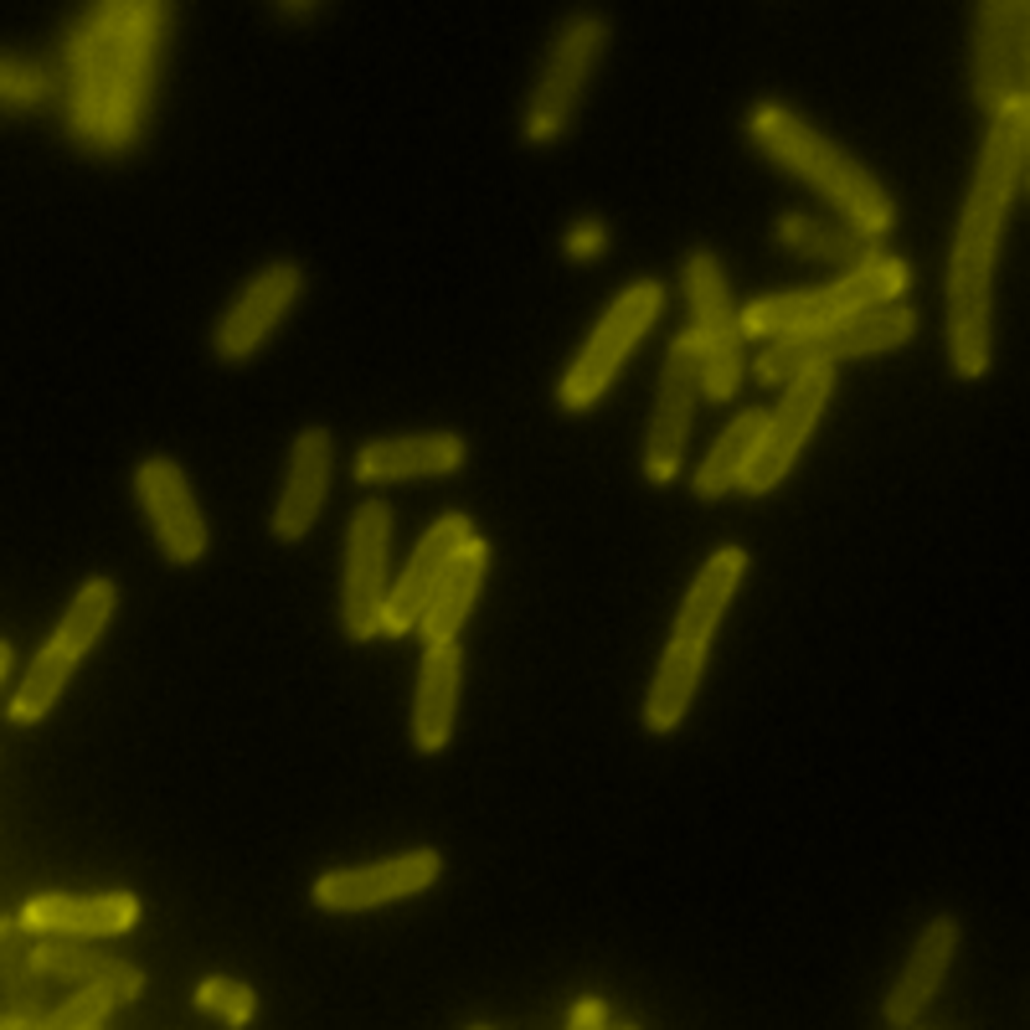

Supplement: Supplementary file 12 — Source Data Fig. 7 [file 44318_2023_26_MOESM12_ESM.zip › Figure 7/7C/tar-yfp dMin.tif]

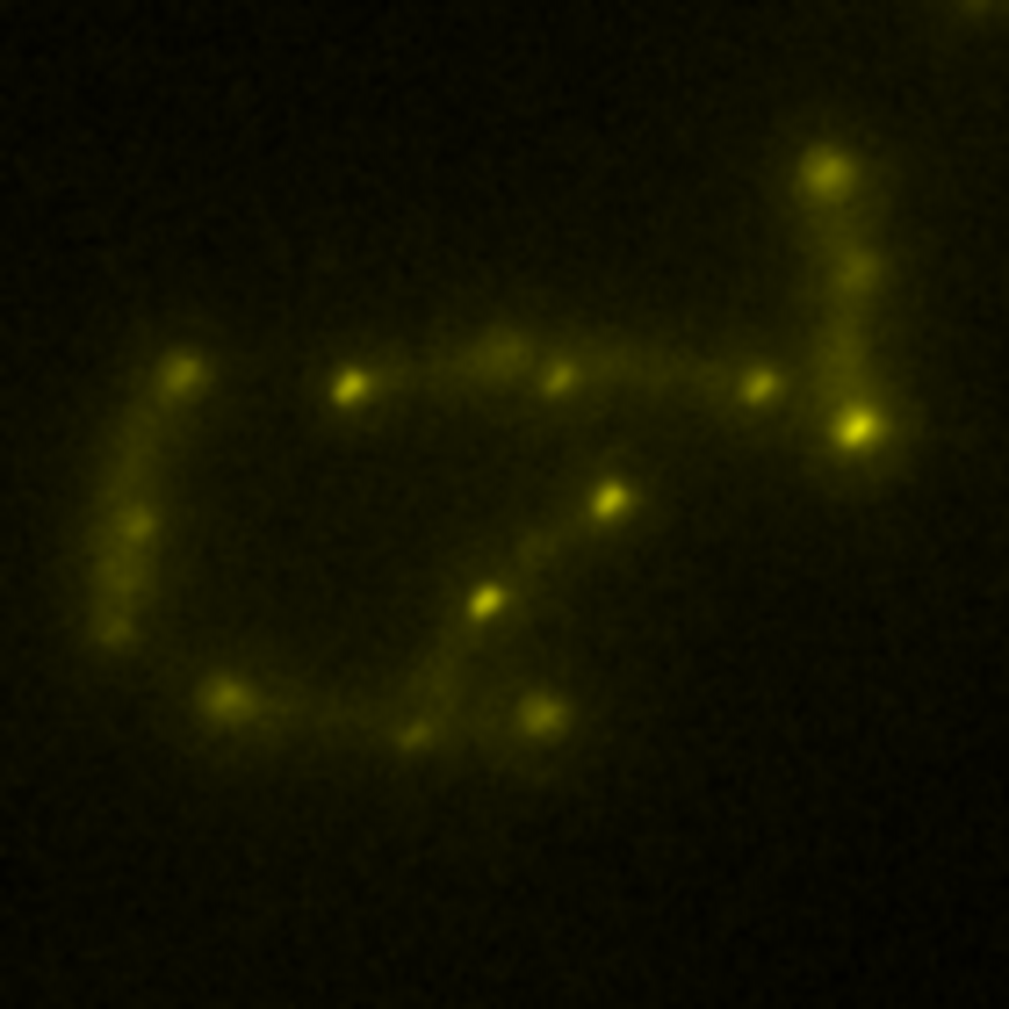

Supplement: Supplementary file 12 — Source Data Fig. 7 [file 44318_2023_26_MOESM12_ESM.zip › Figure 7/7B/Ceph.tif]

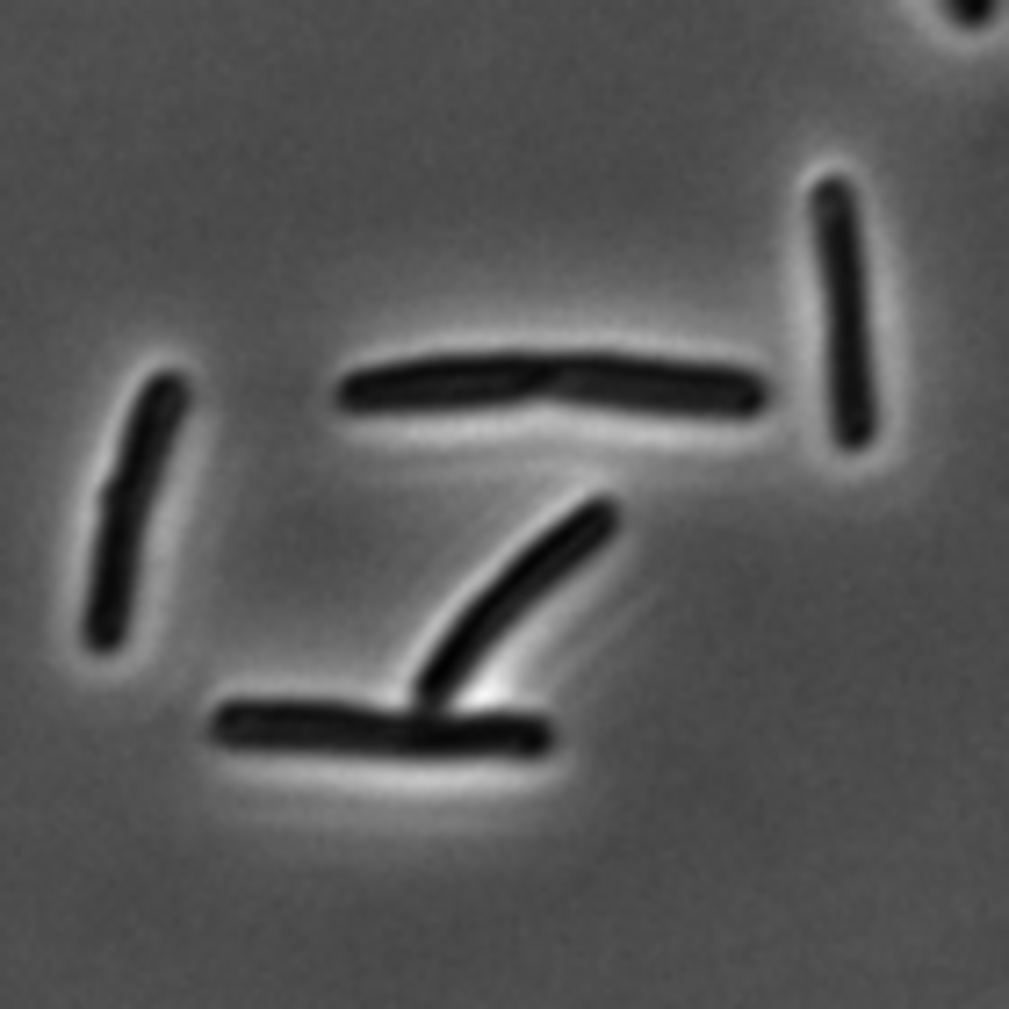

Supplement: Supplementary file 12 — Source Data Fig. 7 [file 44318_2023_26_MOESM12_ESM.zip › Figure 7/7B/Ceph1.tif]

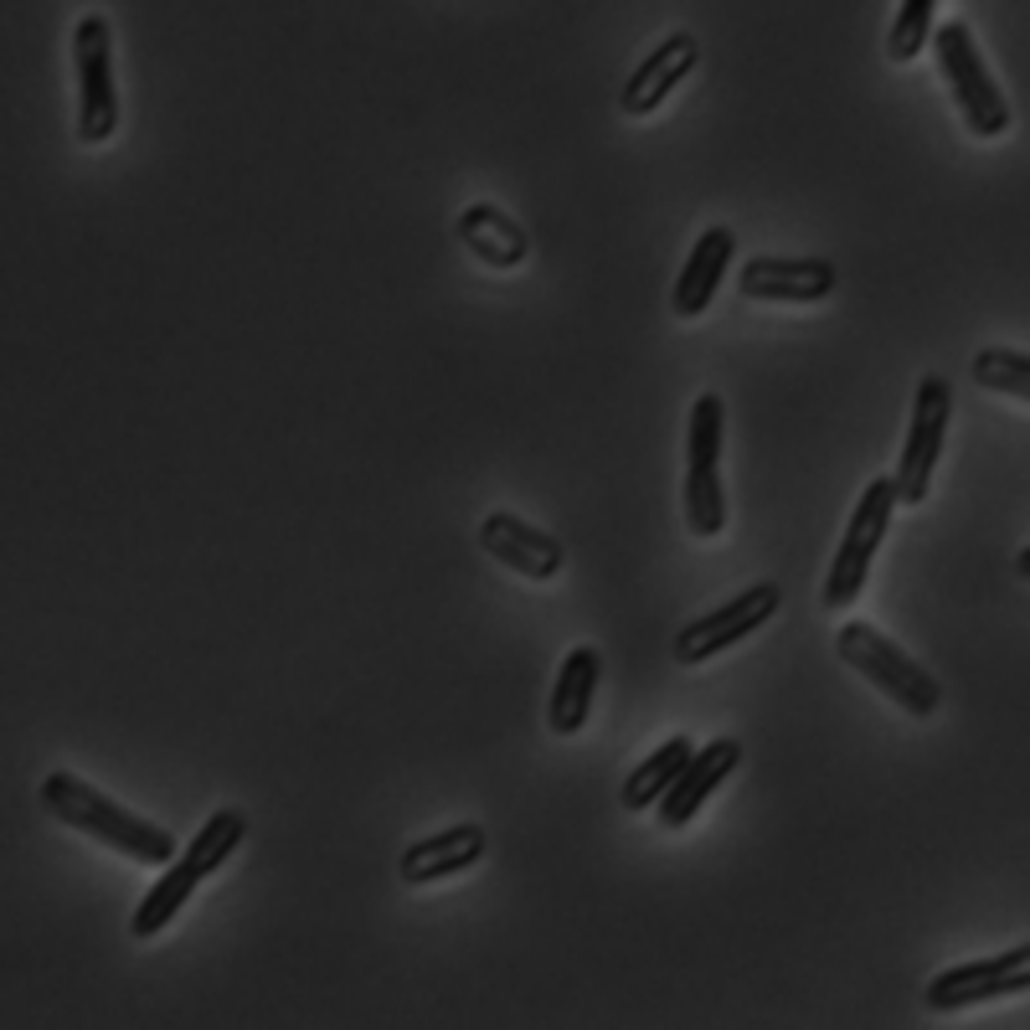

Supplement: Supplementary file 12 — Source Data Fig. 7 [file 44318_2023_26_MOESM12_ESM.zip › Figure 7/7A/cheA WT1.tif]

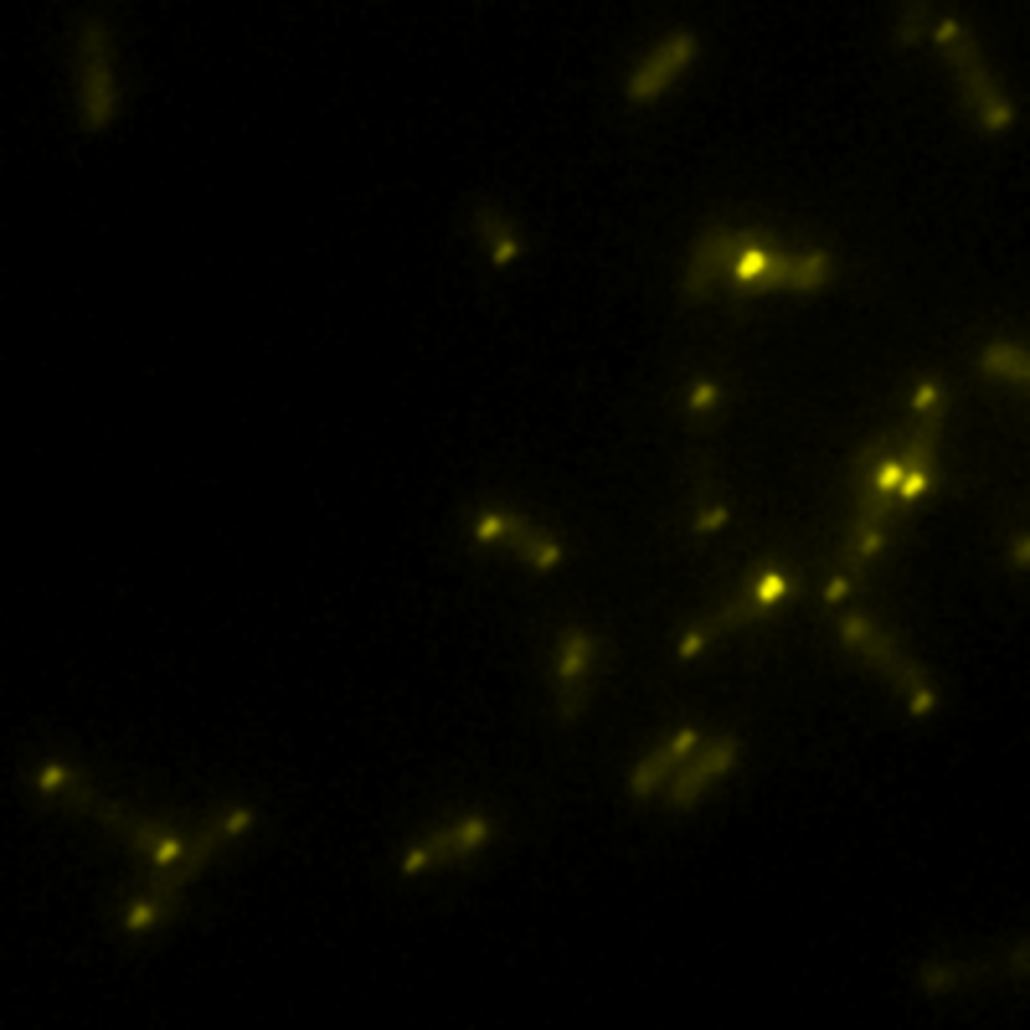

Supplement: Supplementary file 12 — Source Data Fig. 7 [file 44318_2023_26_MOESM12_ESM.zip › Figure 7/7A/cheA WT.tif]

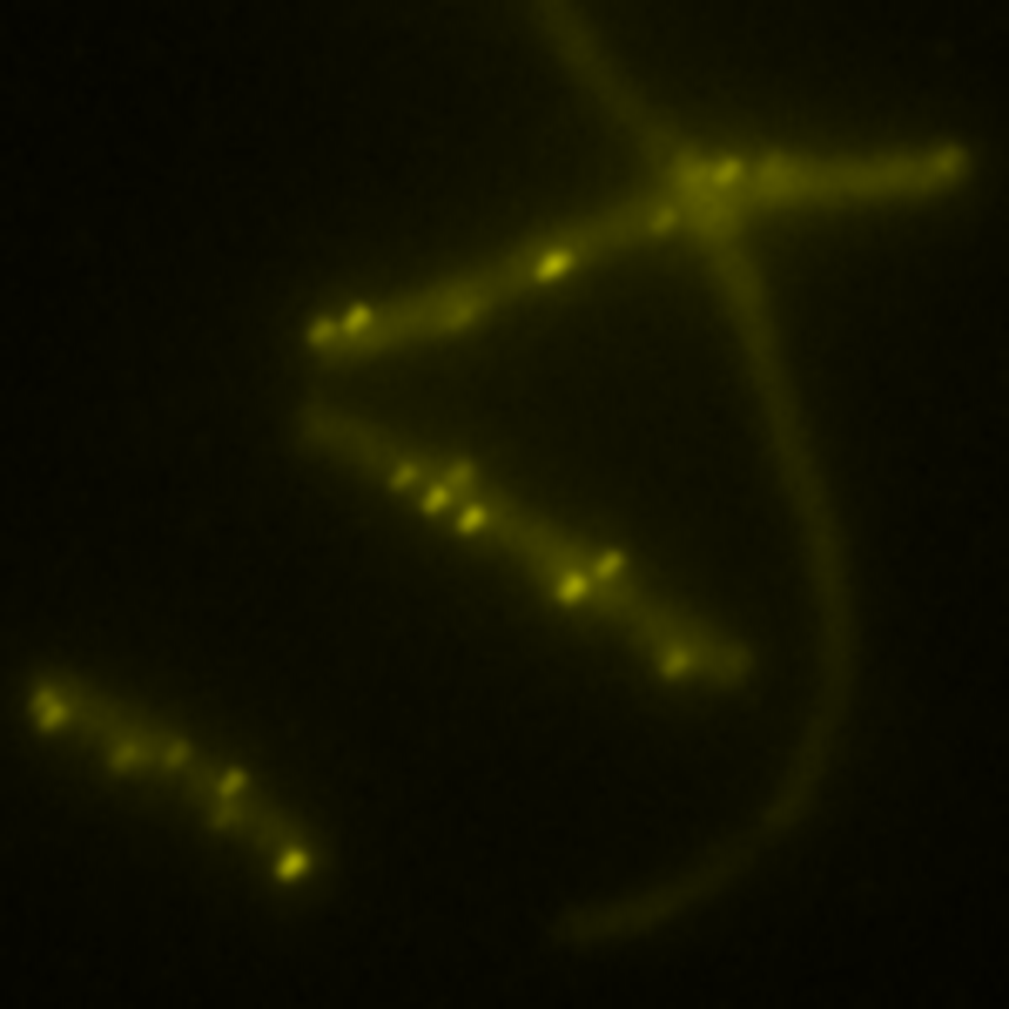

Supplement: Supplementary file 12 — Source Data Fig. 7 [file 44318_2023_26_MOESM12_ESM.zip › Figure 7/7A/cheA dMin.tif]

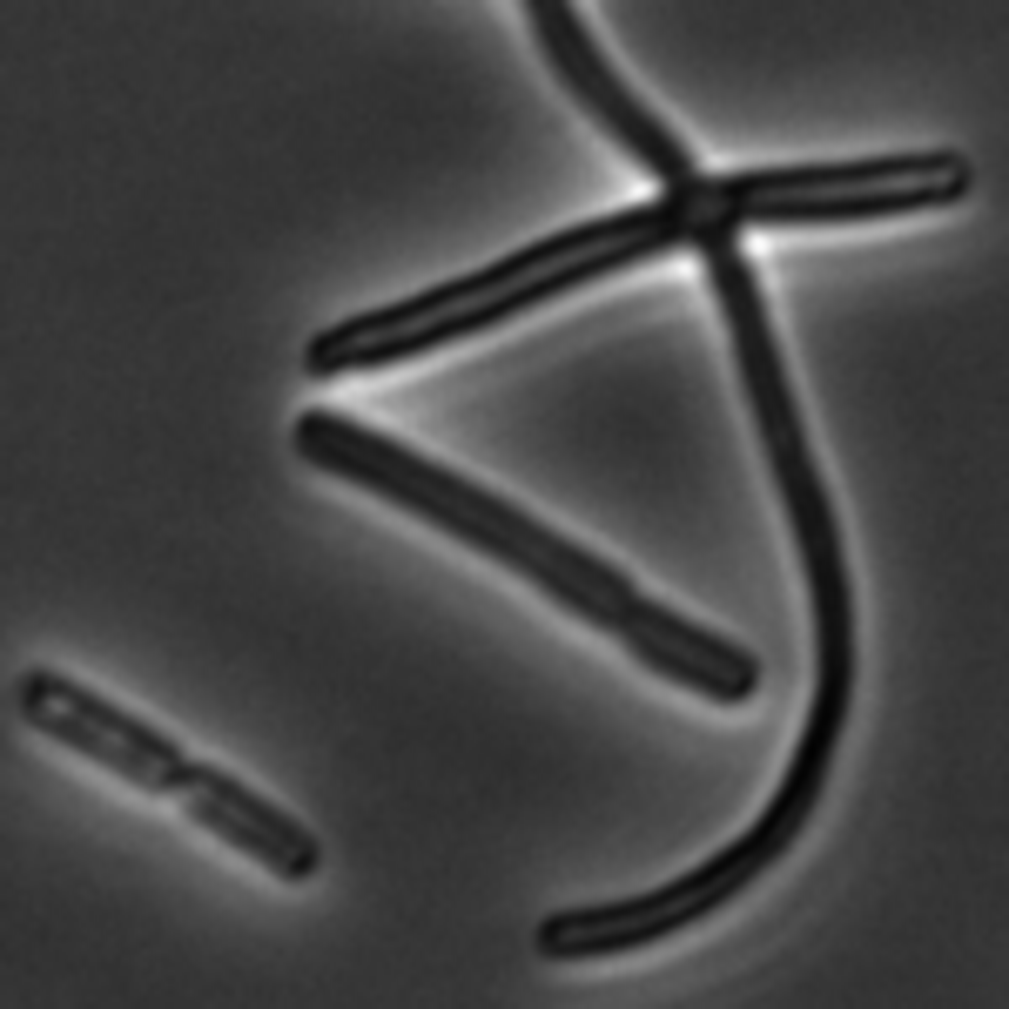

Supplement: Supplementary file 12 — Source Data Fig. 7 [file 44318_2023_26_MOESM12_ESM.zip › Figure 7/7A/cheA dMin1.tif]
