# Supplementary material for: MinD-RNase E interplay controls localization of polar mRNAs in E. coli
Source: EMBO J. 2024 Jan 19;43(4):8. doi: 10.1038/s44318-023-00026-9 (PMC10897333; doi:10.1038/s44318-023-00026-9)
Supplement: Supplementary file 14 — Expanded View Figures [file 44318_2023_26_MOESM14_ESM.pdf]

Expanded View Figures

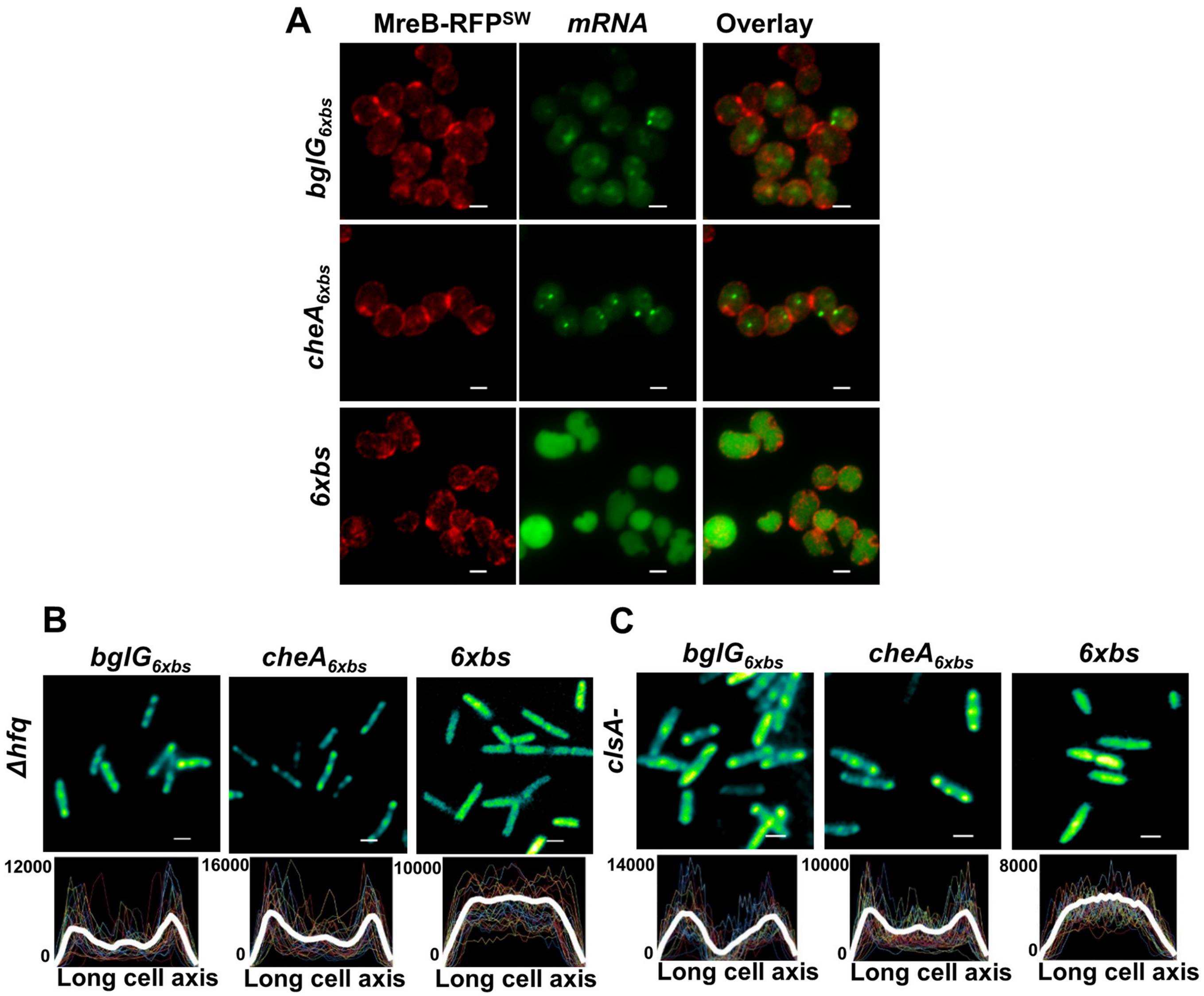

Figure EV1. MreB, RNA chaperone Hfq, and cardiolipin are not involved in the localization of polar mRNAs (related to Fig. 1).

(**A**) Images of *bglG*, *cheA* and 6xbs in A22-treated cells, which also express MreB-RFP^{SW}. Overlays of MreB (RFP) and mRNA (GFP) are also displayed. (**B**, **C**) Upper panels: Images showing localization of representative polar transcripts, *bglG*, *cheA*, as well as no mRNA control (6xbs) in Δ*hfq* and *clsA*- strain background, respectively. Lower panels: The average fluorescence intensity profiles of the transcripts plotted against the long cell axis after normalizing to cell length; $n = 50–60$ in both cases. mRNAs were detected in live cells by the MS2 system. Images are representatives of biological triplicates (**A**, **B**). Scale bar corresponds to 2 μm.

 © The Author(s)

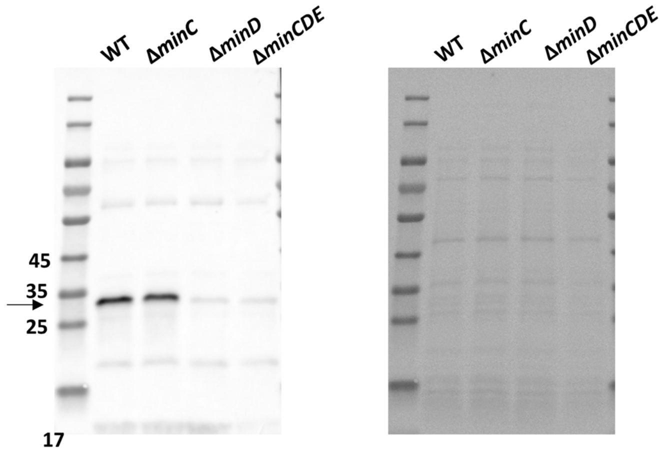

Figure EV2. Deletion of the *minC* gene does not affect the expression of *minD* (related to Fig. 1).

Western blot analysis detecting MinD expression in log phase of Δ*minC*, Δ*minD* and Δ*minCDE* compared to wild type cells (left panel). An equal number of cells were sampled and blotted onto the membrane, as can be seen in the Ponceau S staining of the membrane before probing (right panel). The membrane was probed with anti-MinD antiserum. The band corresponding to MinD is indicated with an arrowhead (29.6 kDa).

© The Author(s)

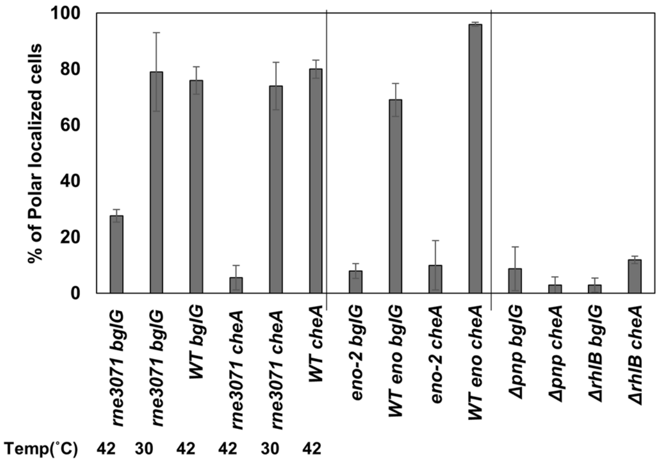

Figure EV3. Quantification of the spatial distribution of polar transcripts in cells deleted or disrupted for functional activity of degradosome components (related to Fig. 2).

Bar plots showing the percentage of pole-localized transcripts in the different strains deleted or disrupted for the degradosome components in the experiment presented in Fig. 2. SEM error bars are presented for each sample. $n > 200$ for each strain.

© The Author(s)

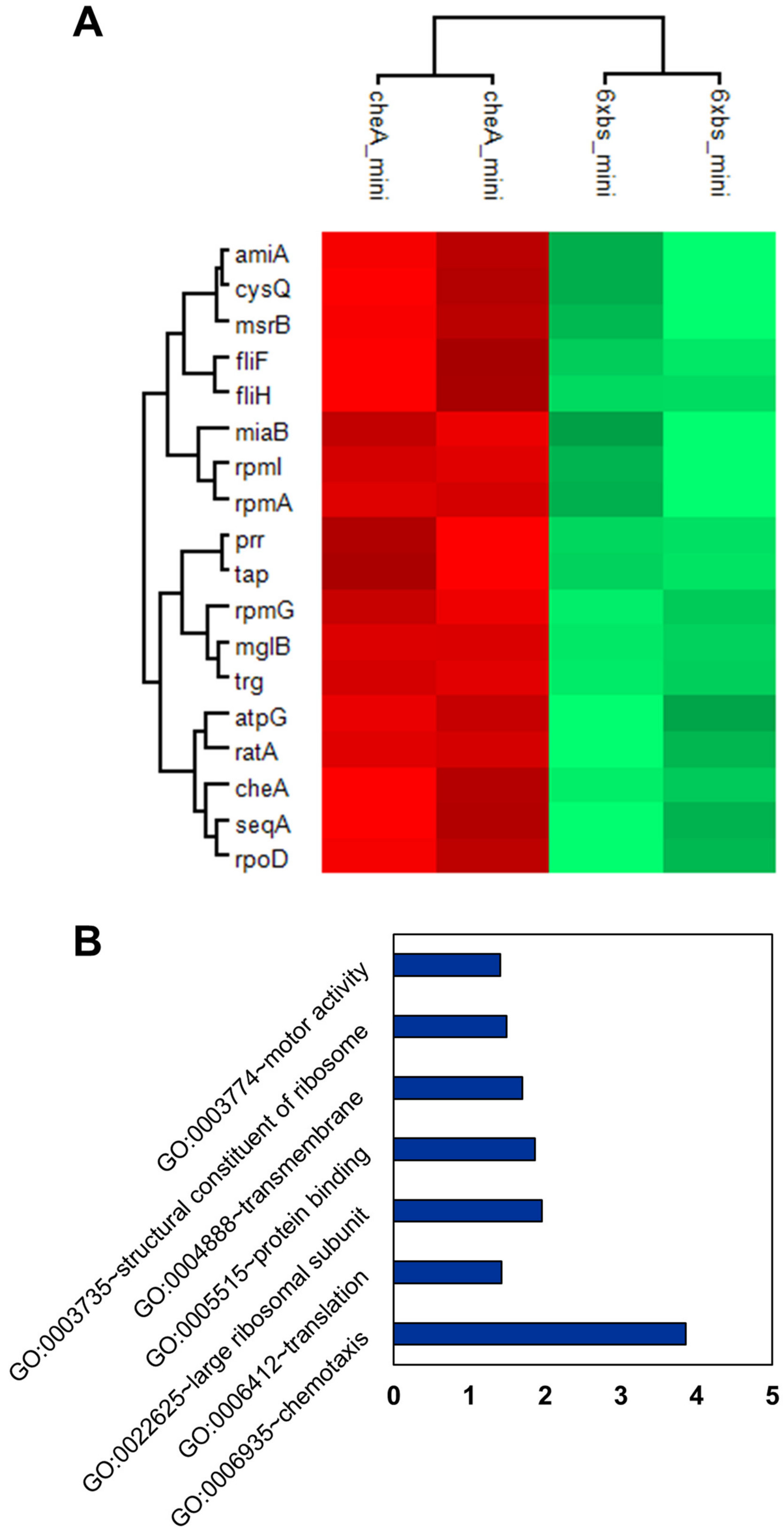

A. Heat map showing differentially enriched proteins that were pulled down with the *cheA* mRNA and with the no mRNA control (6xbs); p value<0.01.

B. Graphs showing statistically significant GO terms for the immunoprecipitated proteins that were identified by mass spectrometry as binding to the *cheA* RNA in minicells.

Figure EV4. High-throughput proteomics screen to identify *cheA* mRNA-binding proteins (related to Fig. 3).

(**A**) Heat map showing differentially enriched proteins that were pulled down with the *cheA* mRNA and with the no mRNA control (6xbs); *p* value < 0.01. (**B**) Graphs showing statistically significant GO terms for the immunoprecipitated proteins that were identified by mass spectrometry as binding to the *cheA* RNA in minicells.

© The Author(s)

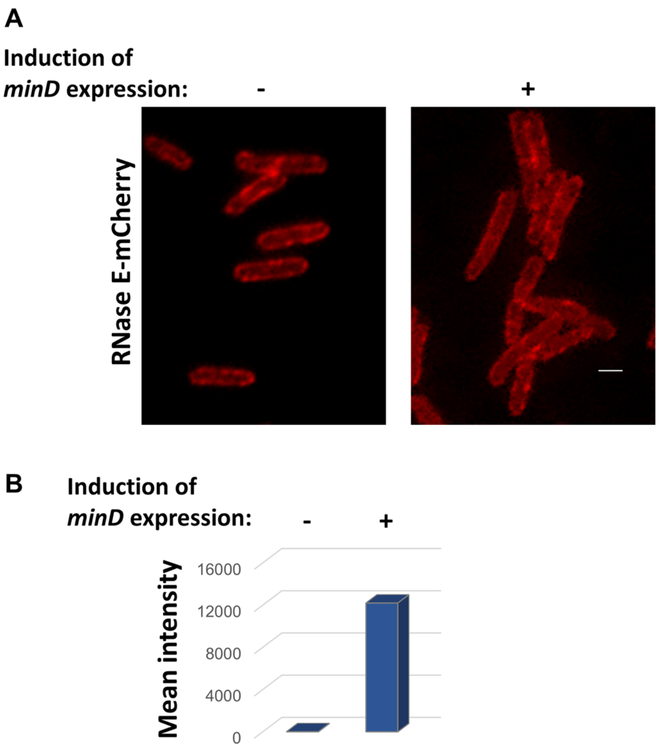

Figure EV5. MinD overexpression does not alter RNase E localization (related to Fig. 4).

(**A**) Images showing live cells expressing RNase E-mCherry from the chromosome and MinD from a plasmid with or without induction. Scale bar corresponds to 2 μm. (**B**) Bar plot showing the mean intensity of MinD-GFP expression with or without induction.

 © The Author(s)
